# Supplementary material for: A draft human pangenome reference
Source: Nature. 2023 May 10;617(7960):312–24. doi: 10.1038/s41586-023-05896-x (PMC10172123; doi:10.1038/s41586-023-05896-x)
Supplement: Supplementary file 1 — Supplementary Figs. 1–49 and their legends. [file 41586_2023_5896_MOESM1_ESM.pdf]

nature portfolio

https://doi.org/10.1038/s41586-023-05896-x

Supplementary information

A draft human pangenome reference

In the format provided by the authors and unedited

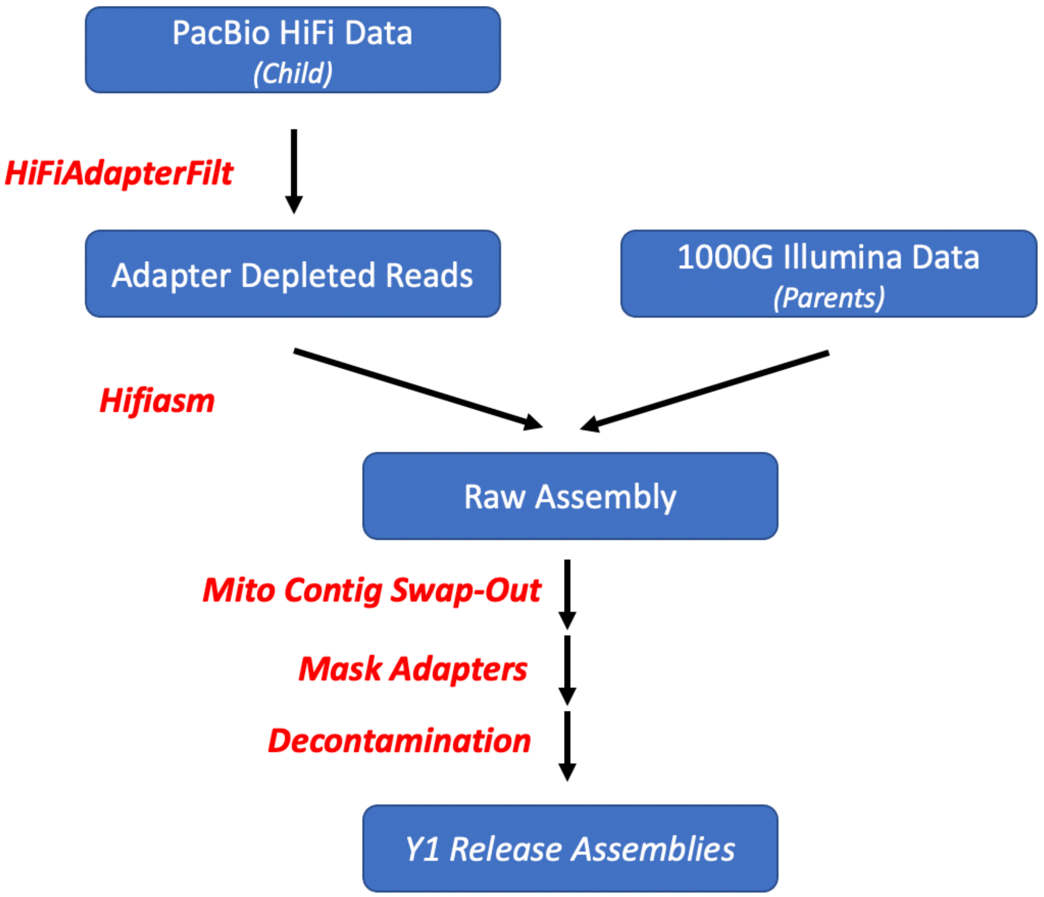

Supplementary Figure 1 | Trio-Hifiasm based assembly pipeline overview.

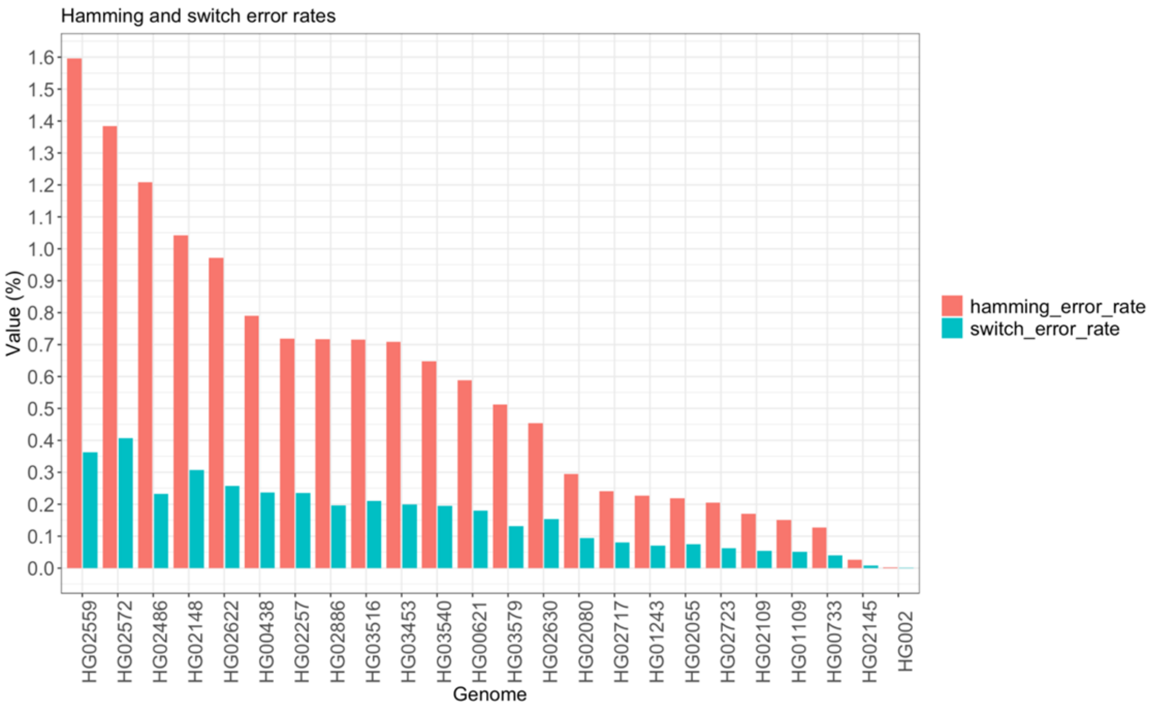

Supplementary Figure 2 | Hi-C based phasing analysis using pstools. The x and y axis represent the samples and error rate values respectively. The red and blue bars show the hamming and switch error rates respectively.

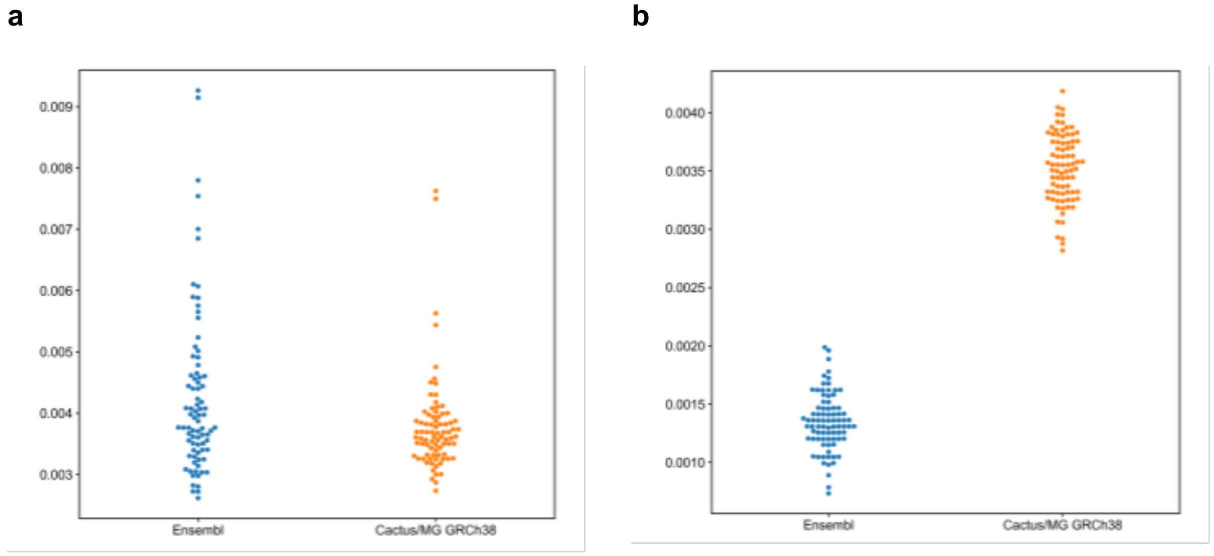

Supplementary Figure 3 | Genes with frameshift mutations or nonsense mutations in the pangenome graphs. a, Fraction of canonical transcripts with a frameshifting indel from GENCODE v38 in the Ensembl annotations and the CAT annotations of the GRCh38-based pangenome graph. **b**, Fraction of canonical transcripts with early in-frame stop codons in the Ensembl annotations and the CAT annotations of the GRCh38-based pangenome graph.

a

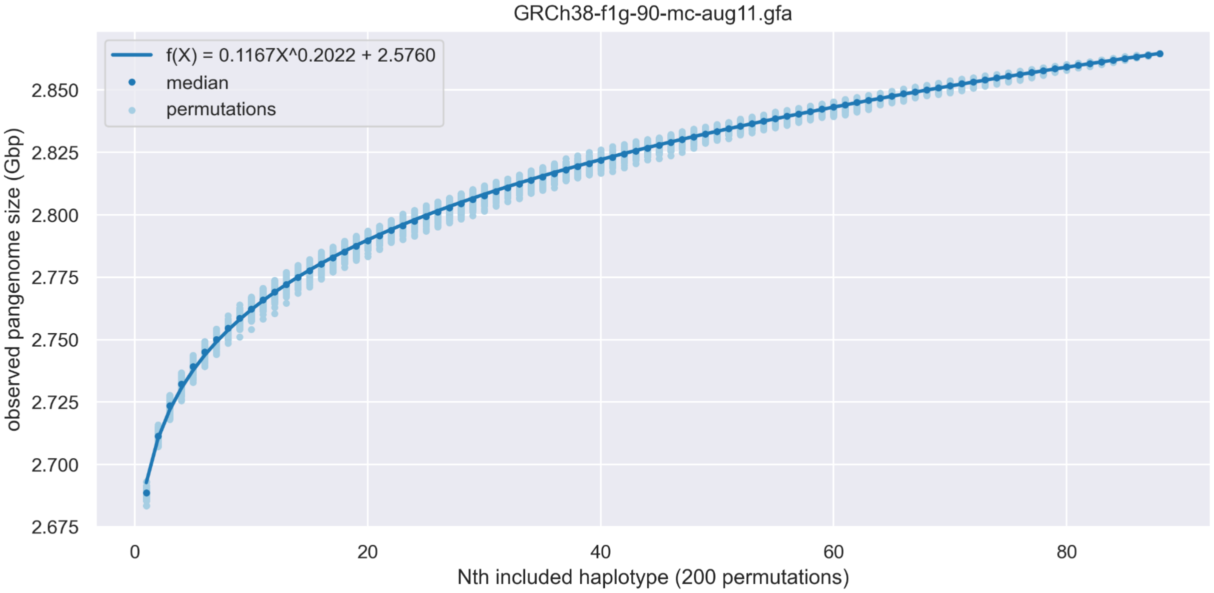

b

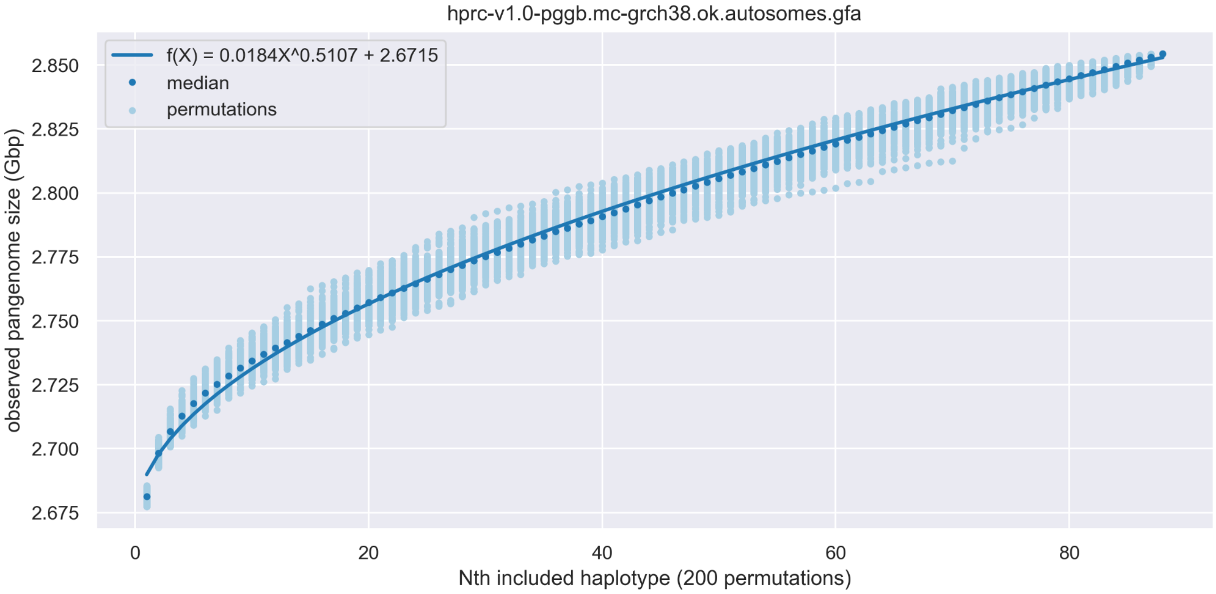

Supplementary Figure 4 | Pangenome saturation curves for the autosomal pangenome of the MC graph (**a**) and the PGGB subset to the segments contained in the MC graph (**b**) . The optimal saturation curve (m=0.1167 γ=0.2022 b=2.576) in the MC graph intercepts N=1 at 2.69Gbp,indicating an open pangenome. For the PGGB graph we observe an even amplified degree of openness, with m=0.0184 γ=0.5107 b=2.6715.

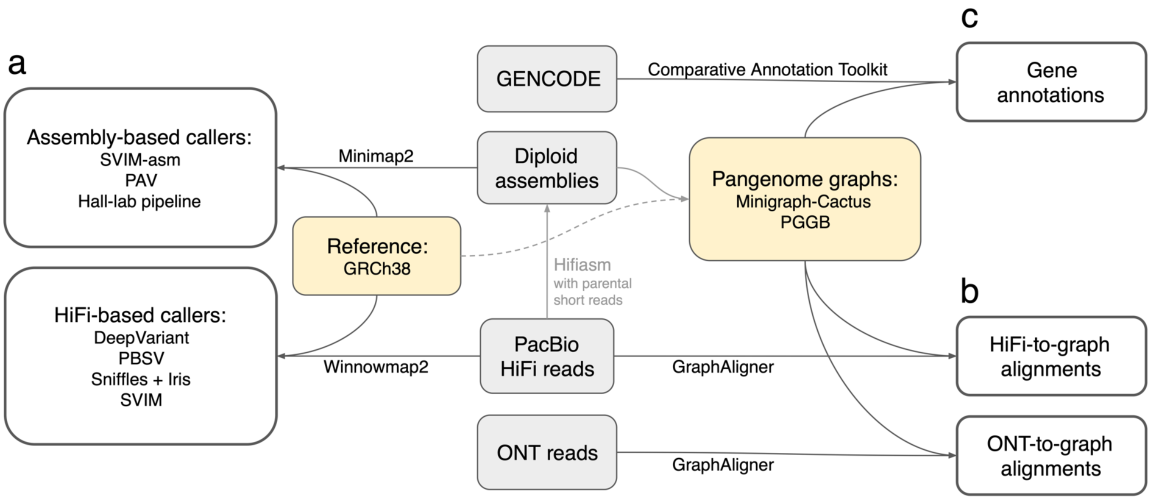

Supplementary Figure 5 | Overview of the evaluation scheme. a, Comparing variants detected from pangenome graphs with truth sets generated by seven discovery methods. **b**, Measuring the number of long reads supporting each node and edge of pangenome graphs. **c**, Projecting the GENCODE annotations onto the assemblies in pangenome graphs.

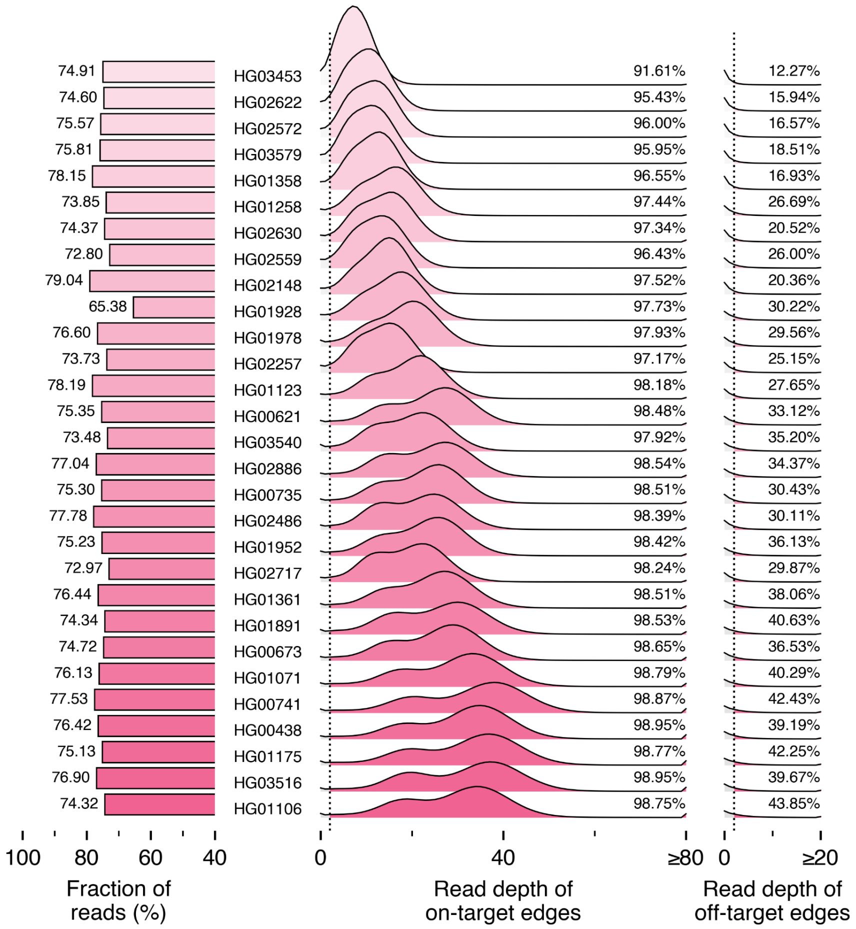

Supplementary Figure 6 | ONT read depth of on- and off-target edges in the MC graph. Left: fraction of reads aligned to the pangenome graph after filtering low-quality alignments. Middle: read depth distribution of on-target edges. Right: read depth distribution of off-target edges. Samples are sorted by sequencing coverage (Supplementary Table 1).

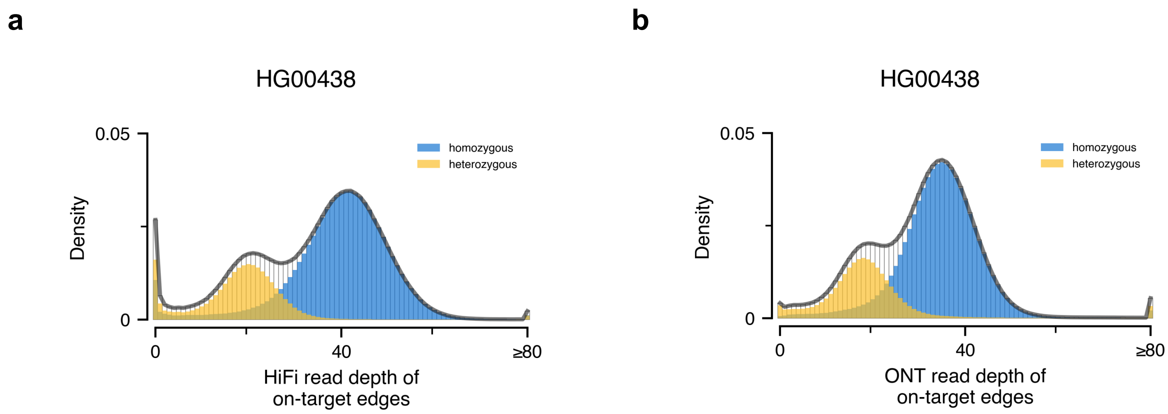

Supplementary Figure 7 | On-target edge coverage. Homozygous and heterozygous edge coverage of sample HG00438 in the MC graph based on (**a**) HiFi reads and (**b**) ONT reads.

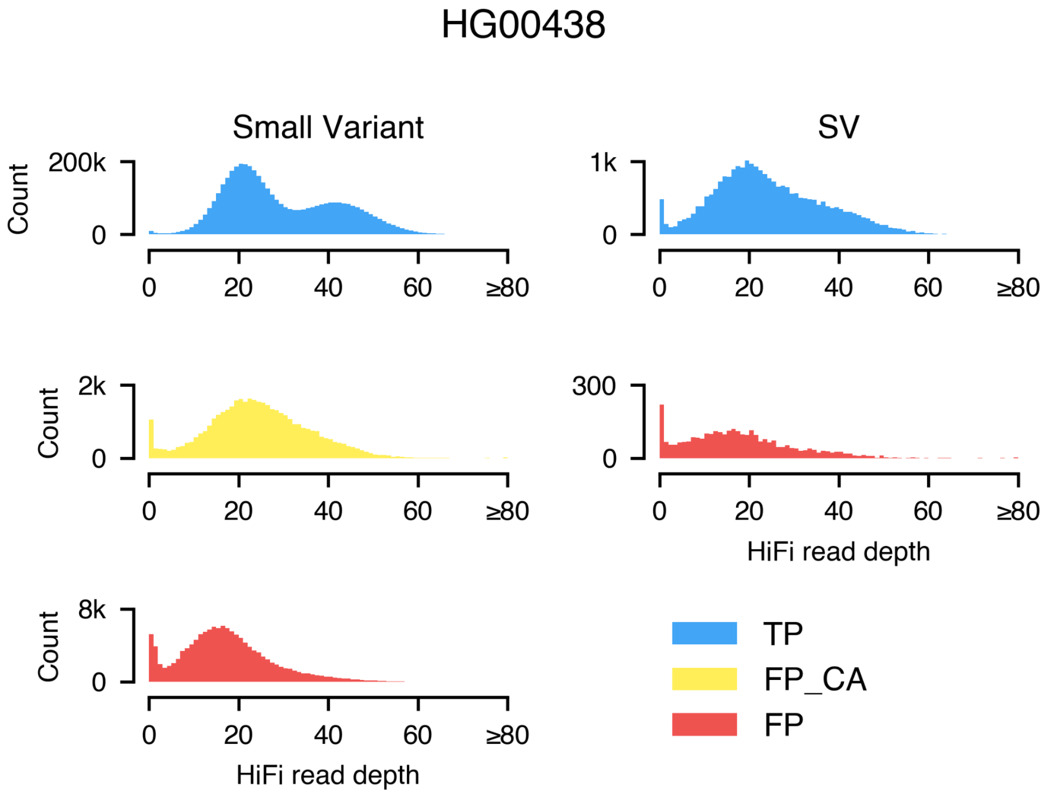

Supplementary Figure 8 | HiFi read depth distribution. The HiFi read depth of HG00438 variants in the MC graph stratified by variant type and by benchmarking classification. TP: true positive. FP_CA: false positive with common allele, i.e. one-sided haplotype match. FP: false positive.

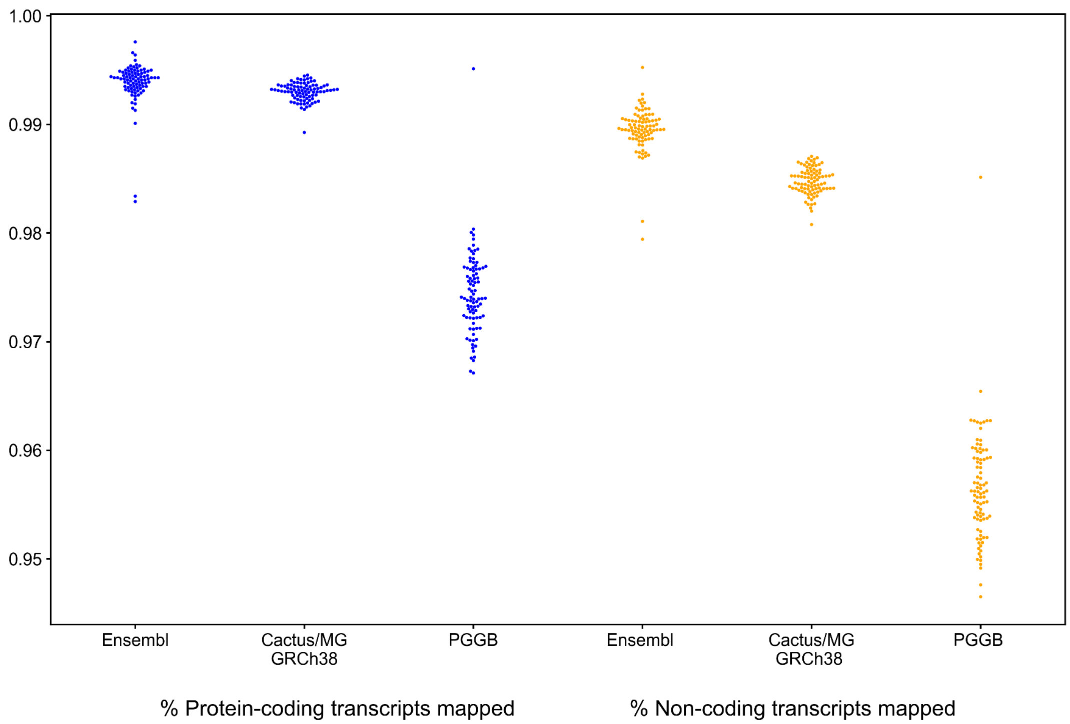

Supplementary Figure 9 | Transcript mapping in the pangenome graphs. The first three show the percentage of protein-coding transcripts from GENCODE v38 able to be mapped in the gene annotation sets from Ensembl, CAT run on the MC graph based on GRCh38, and CAT run on the PGGB graph. The second three show the percentage of non-coding transcripts from GENCODE v38 able to be mapped on the same annotation sets.

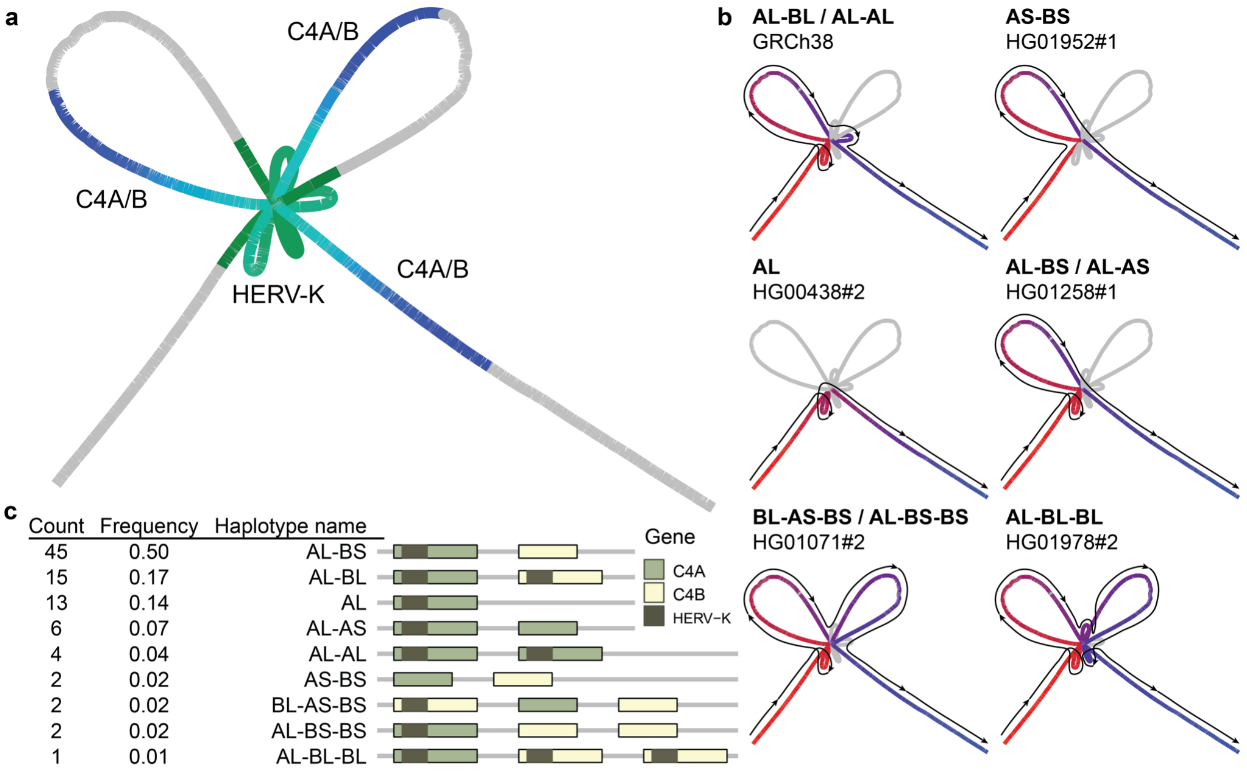

Supplementary Figure 10 | Structural haplotypes of C4 called from the MC graph. a, Location of C4A and/or C4B genes in the MC subgraph. Color gradient is based on the relative position of a gene. Green represents the head of a gene. Blue represents the end of a gene. **b**, Different structural haplotypes take different paths in the graph. Color gradient is based on path position. Red represents the head of a path. Blue represents the end of a path. **c**, Frequency and linear structural visualization of all structural haplotypes called by the MC graph.

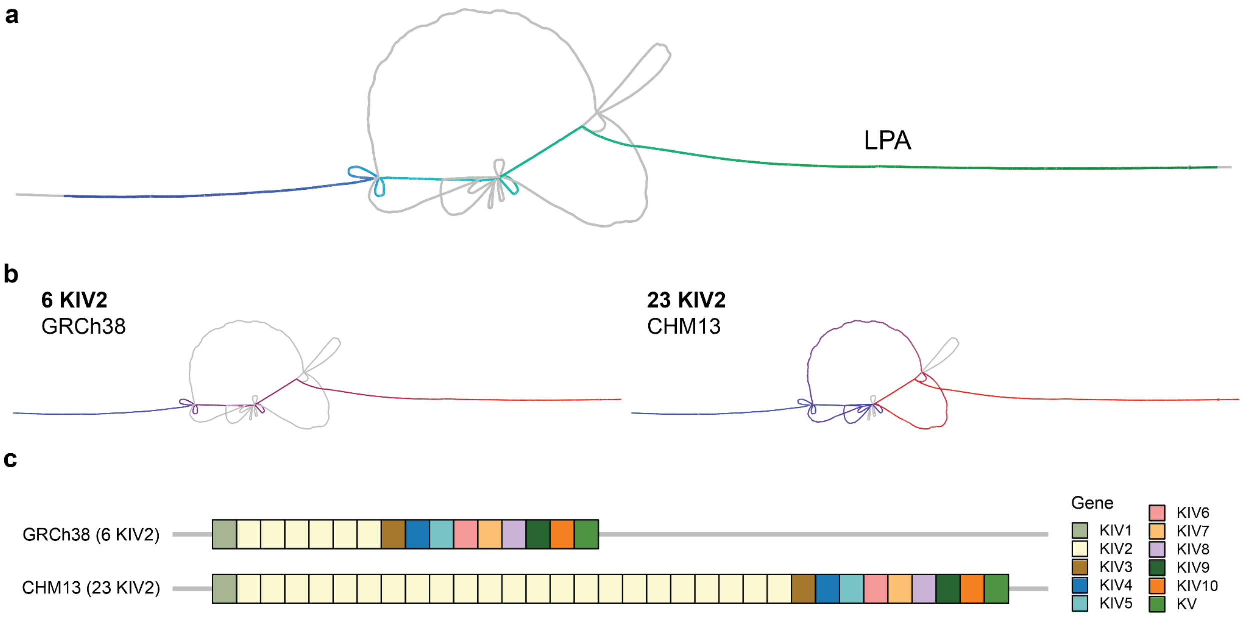

Supplementary Figure 11 | Structural haplotypes of LPA called from the MC graph. a, Location of LPA genes in the MC subgraph. Color gradient is based on the relative position of a gene. Green represents the head of a gene. Blue represents the end of a gene. **b**, Different structural haplotypes take different paths in the graph. Color gradient is based on path position. Red represents the head of a path. Blue represents the end of a path. **c**, Linear structural visualization of two typical structural haplotypes of LPA with different numbers of KIV-2 repeats called by the MC graph.

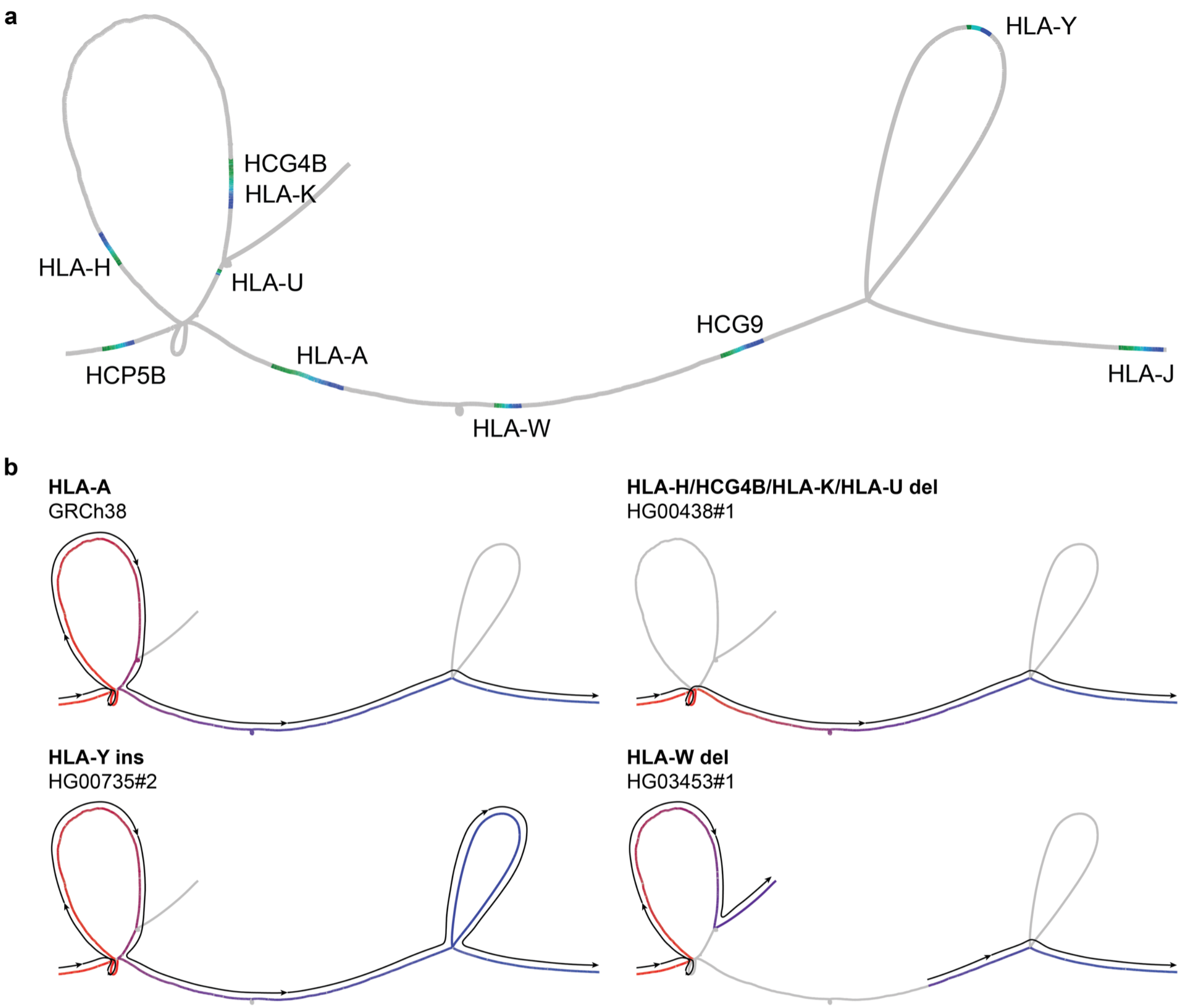

Supplementary Figure 12 | Structural haplotypes of HLA-A called from the MC graph. a, Location of genes within the MC subgraph. Color gradient is based on the relative position of a gene. Green represents the head of a gene. Blue represents the end of a gene. **b**, Different structural haplotypes take different paths through the graph. Color gradient is based on path position. Red represents the head of a path. Blue represents the end of a path.

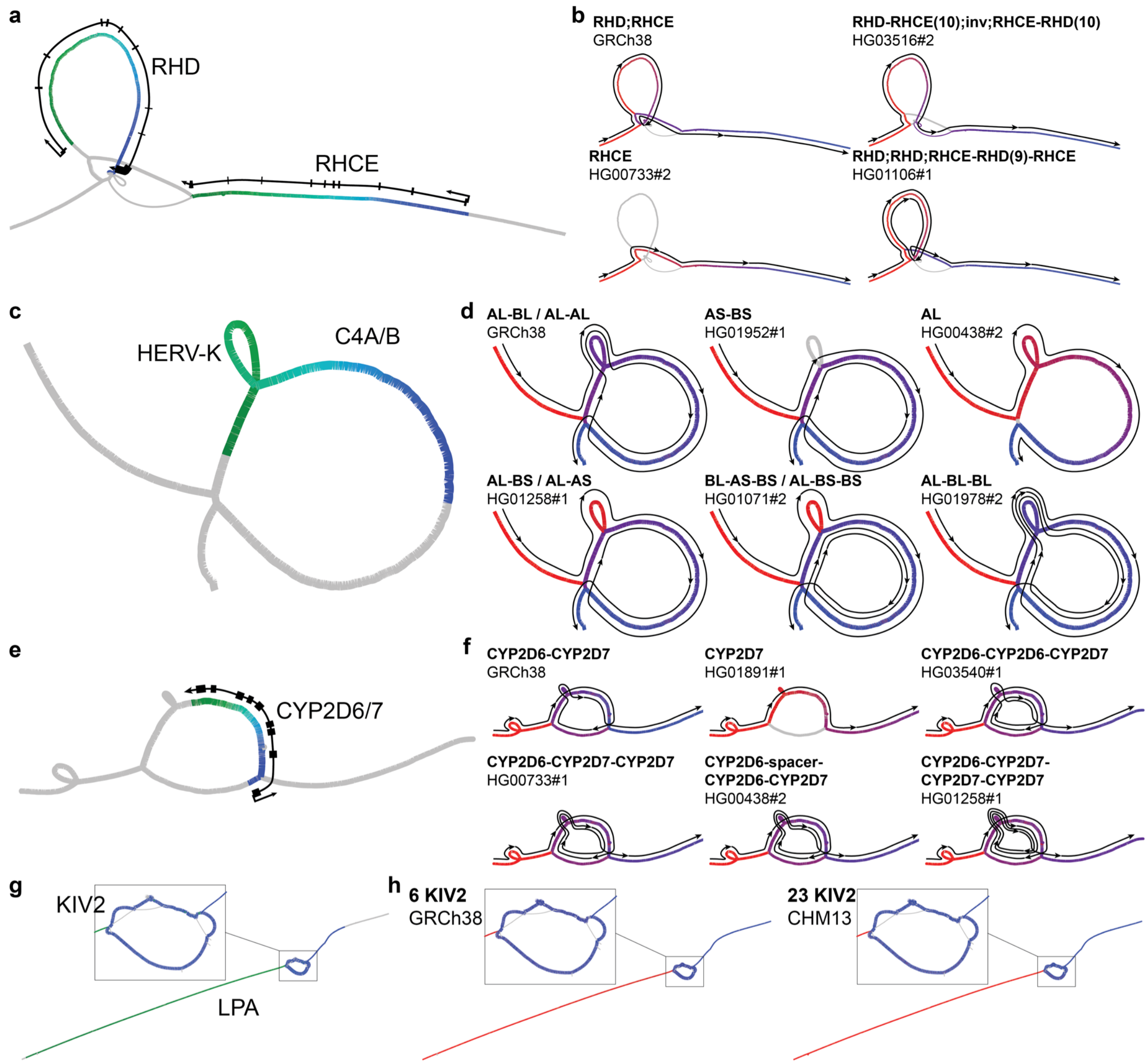

Supplementary Figure 13 | Structural haplotypes of 4 genes called from the PGGB graph. **a**,**c**,**e**,**g**, Location of genes within the RHD, HLA-A, C4, CYP2D6, and LPA loci in the PGGB graph. Color gradient is based on the relative position of a gene. Green represents the head of a gene. Blue represents the end of a gene. **b**,**d**,**f**,**h**, Different structural haplotypes take different paths in the graph. Color gradient is based on path position. Red represents the head of a path. Blue represents the end of a path.

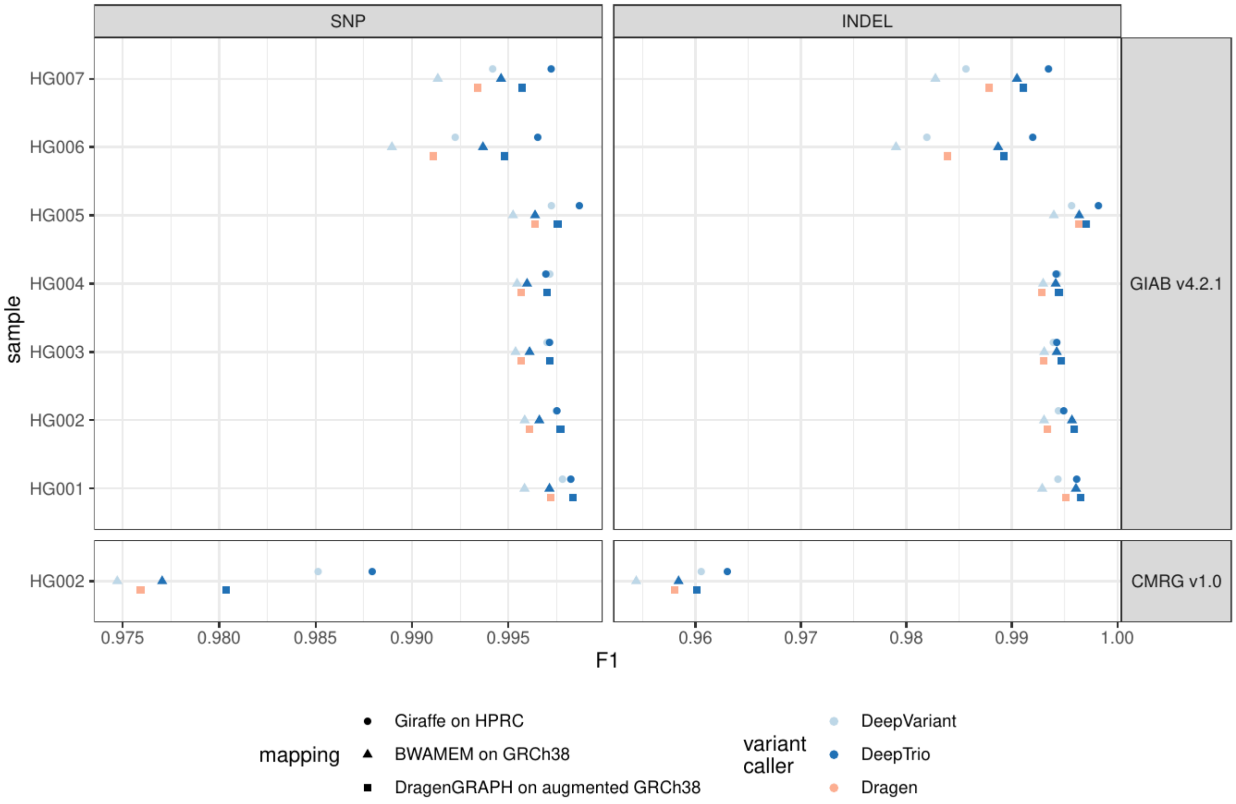

Supplementary Figure 14 | Evaluation of the SNPs (left) and indels (right) calls by different variant callers (colors) from different mapping approaches (shape). The x-axis represents the F1 score when comparing the calls with two truth sets (horizontal panels). The “augmented GRCh38” used by the DragenGRAPH mapper (square points) corresponds to the GRCh38 genome reference plus about 900,000 known population haplotypes blocks.

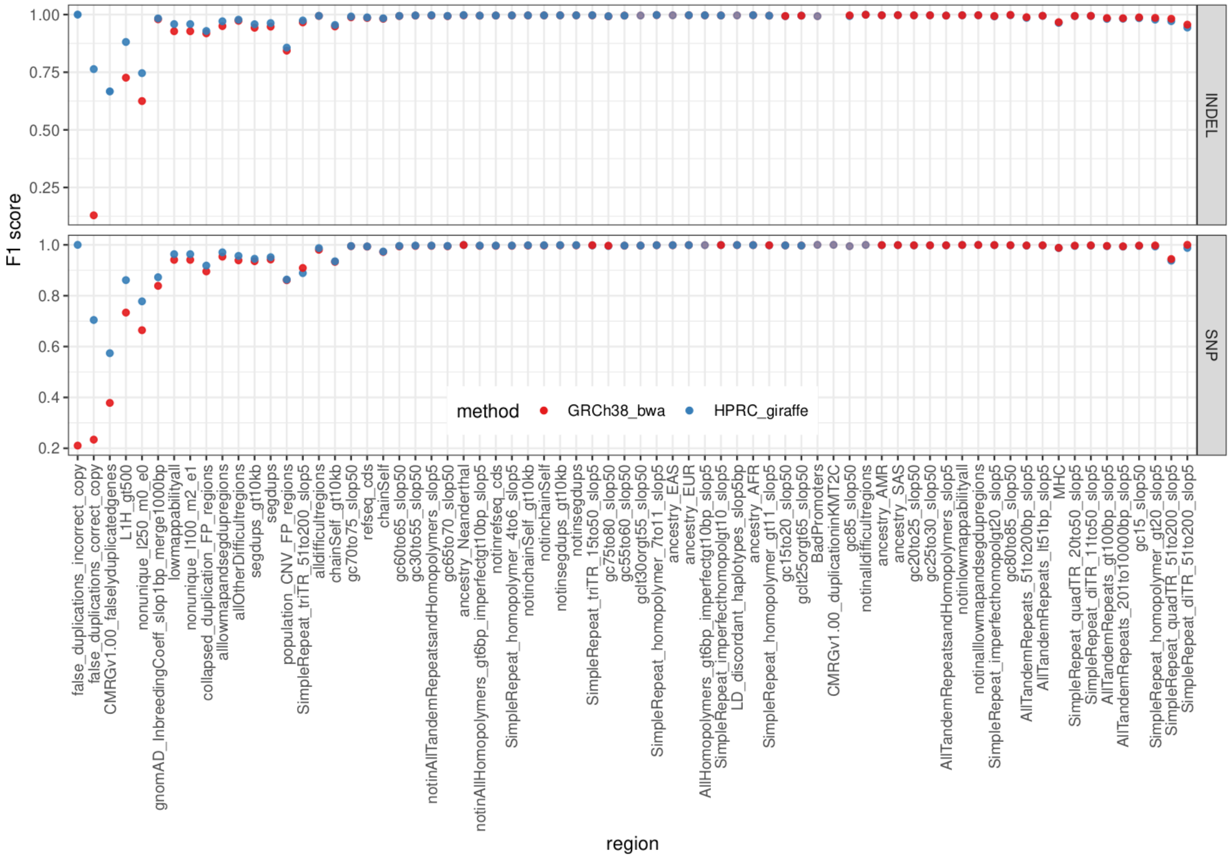

Supplementary Figure 15 | Evaluate the SNPs and indels called on HG003 by Deep Variant from short reads aligned either to GRCh38 with BWA-MEM (red) or the HPRC pangenome with VG Giraffe (blue), stratified by regions (x-axis). The x-axis is ordered to highlight regions with the largest differences in performance on the left. The y-axis represents the F1 score when using the GIAB truth set v4.2.1.

a

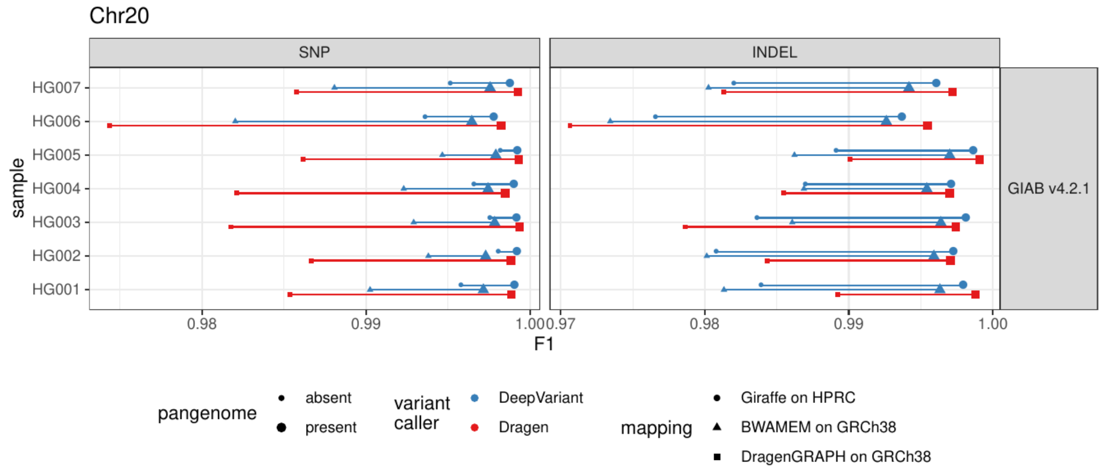

b

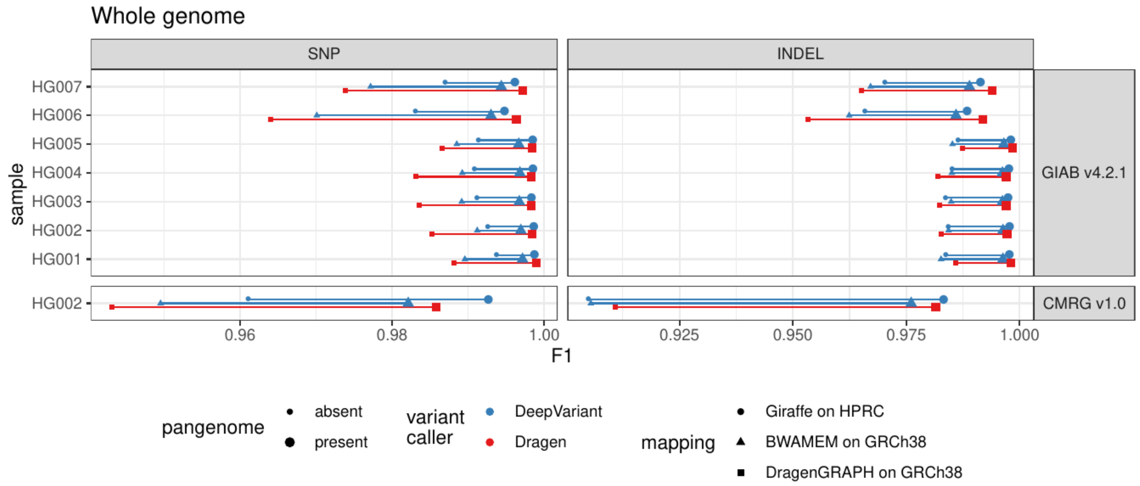

Supplementary Figure 16 | Evaluation of the SNPs (left) and indels (right) calls by different variant callers (colors) from different mapping approaches (shape) stratified by the presence of the variant in the HPRC pangenome (larger point) or not (smaller). The x-axis represents the F1 score when comparing the calls with two truth sets (horizontal panels). The “augmented GRCh38” used by the DragenGRAPH mapper (square points) corresponds to the GRCh38 genome reference plus about 900,000 known population haplotype blocks. The evaluation was performed on variants of chromosome 20 (**a**) or across the whole genome (**b**).

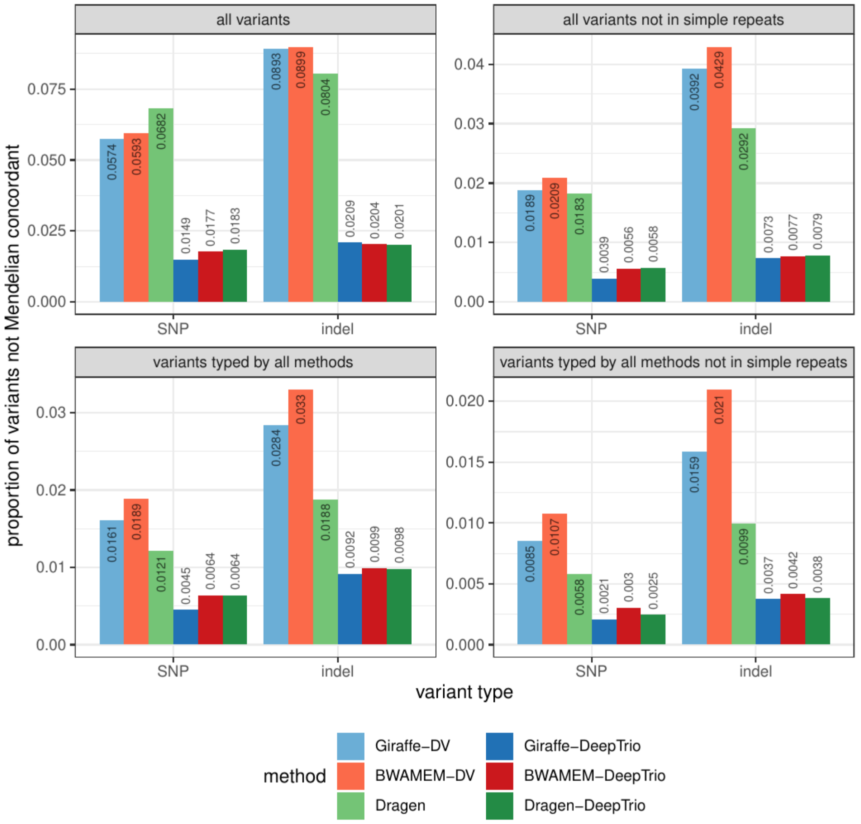

Supplementary Figure 17 | Proportion of SNPs and indels that are not concordant with Mendelian inheritance in the two trios HG002/3/4 and HG005/6/7. Only trios where at least two samples had different genotypes were considered to avoid bias from systematic calls. In the top panels, the analysis was run separately for each method; in the bottom panels the analysis is restricted to sites where all methods called an alternate allele in at least one sample. The left panels consider variants across the whole genome, while variants overlapping simple repeats were excluded from the right panels.

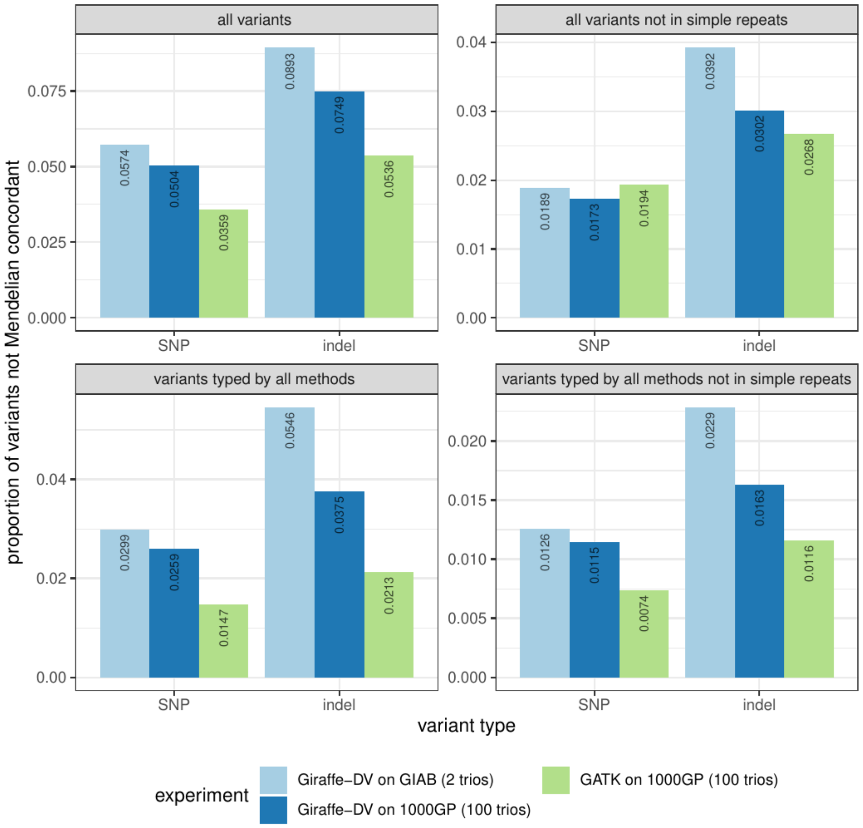

Supplementary Figure 18 | Proportion of SNPs and indels that are not concordant with Mendelian inheritance in the two trios of the GIAB benchmark (HG002/3/4 and HG005/6/7) and 100 trios from the 1000 Genomes Project cohort. Only trios where at least two samples had different genotypes were considered to avoid bias from systematic calls. In the top panels, the analysis was run separately for each method; in the bottom panels the analysis is restricted to sites where all methods called an alternate allele in at least one sample. The left panels consider variants across the whole genome, while variants overlapping simple repeats were excluded from the right panels.

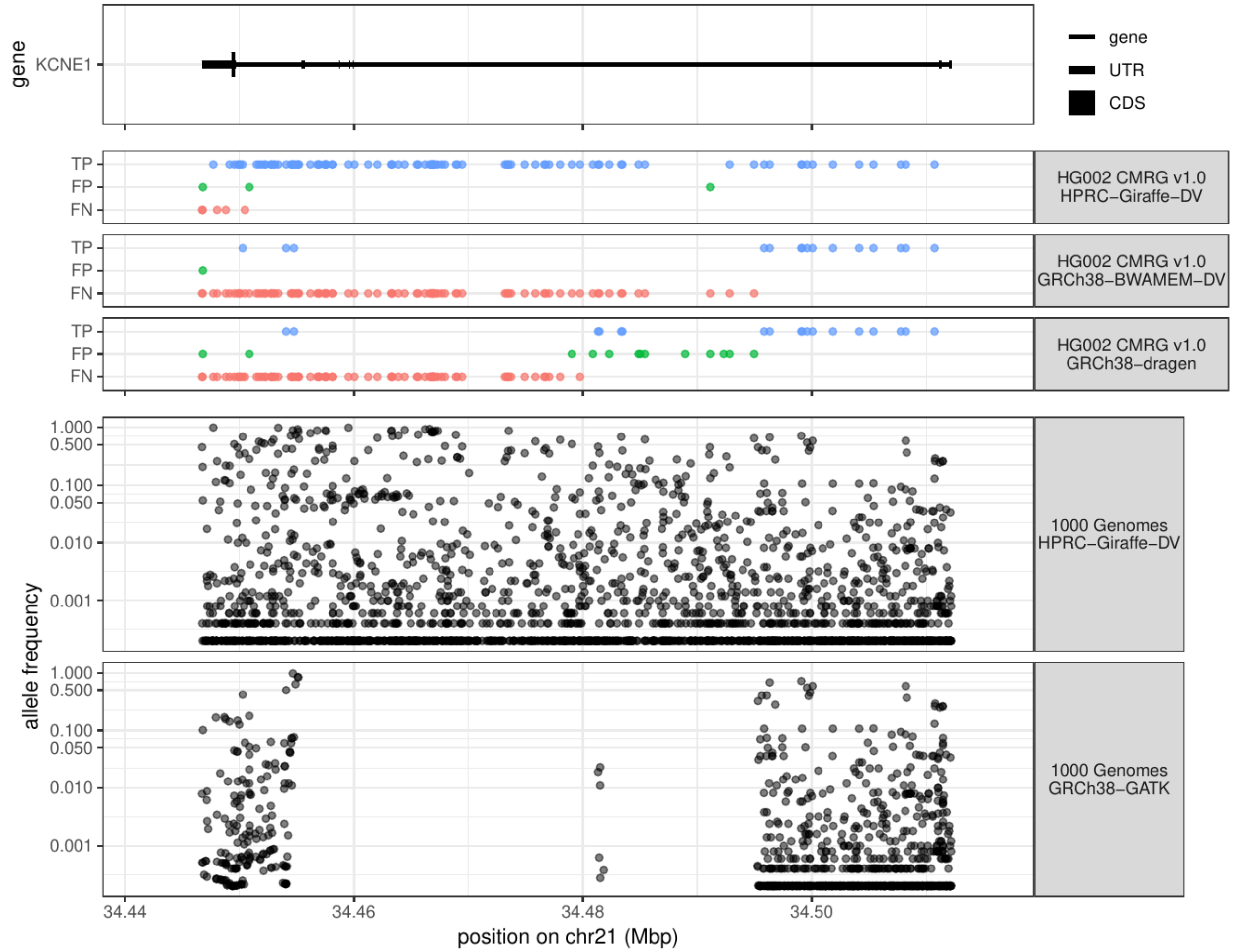

Supplementary Figure 19 | Improved genotyping in the challenging medically-relevant gene KCNE1. a, Gene annotation of the RHCE gene. **b**, Genotyping performance in this region for three approaches (horizontal panels). The top panel, using the HPRC pangenome, shows the best performance with most variants being true positives (TP, blue points) based on the CMRG v1.0 truth set while more other methods have a higher number of false negatives (FN, red points). **c**, Allele frequency across 2,504 unrelated individuals of the 1000 Genomes Project. NOtably, the HPRC-Giraffe-DeepVariant calls (top panel) provide frequencies for the region that is missing with traditional methods due to a false duplication in GRCh38.

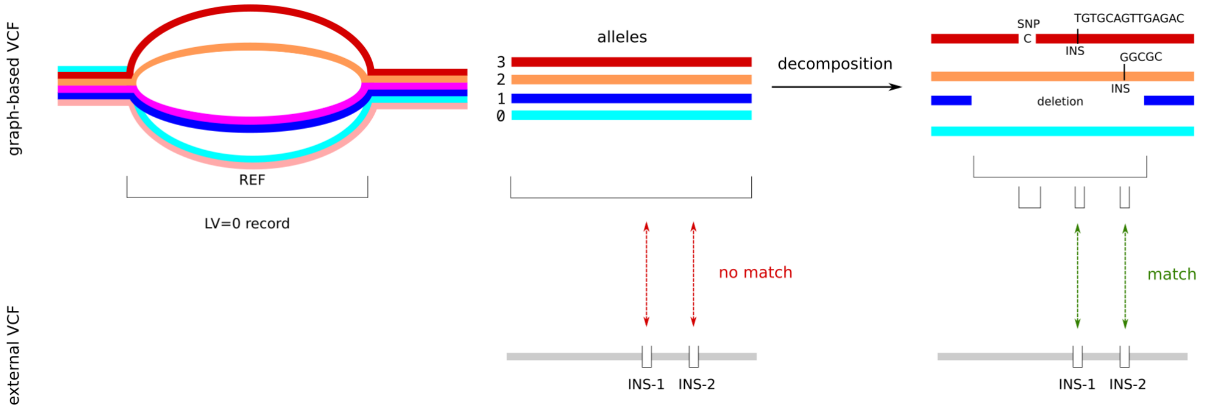

Supplementary Figure 20 | Variant decomposition. Shown is a multi-allelic bubble contained in the snarl-based VCF (LV=0 record). Using the coordinates of the whole bubble when comparing to external callsets leads to errors, since the insertions carried by the second and third haplotypes are not detected. The decomposition aims at identifying which individual variant alleles each haplotype carries inside of the bubble and enables proper comparison to external callsets.

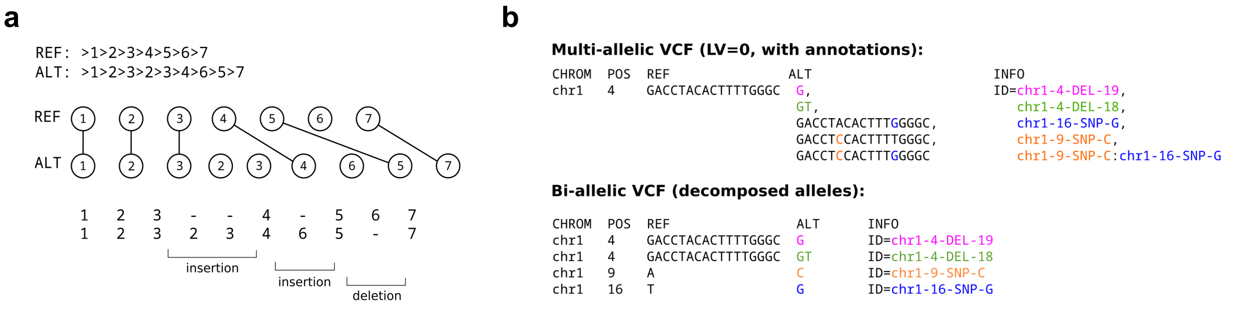

Supplementary Figure 21 | Traversal based decomposition. a, The idea of the decomposition approach is to compare each alternative path in a bubble (that is covered by at least one of the haplotypes present in the graph) to the corresponding reference path. Each node in the reference traversal is matched to its leftmost occurrence in the alternative traversal (if existent), resulting in an alignment of the traversals. The nested alleles can then be determined from the insertions, deletions and mismatches in the alignment. In this example, the alternative allele can be decomposed in two insertions and one deletion. **b**, Two VCF files are produced. The multi-allelic VCF contains the same records as the input VCF, just with annotations for all alternative alleles added to the INFO field. Each ALT allele is annotated by a sequence of IDs encoding the nested alleles, separated by ":". The second VCF is a bi-allelic one, containing a separate record for each nested variant ID, i.e. it contains all alleles after decomposition.

a

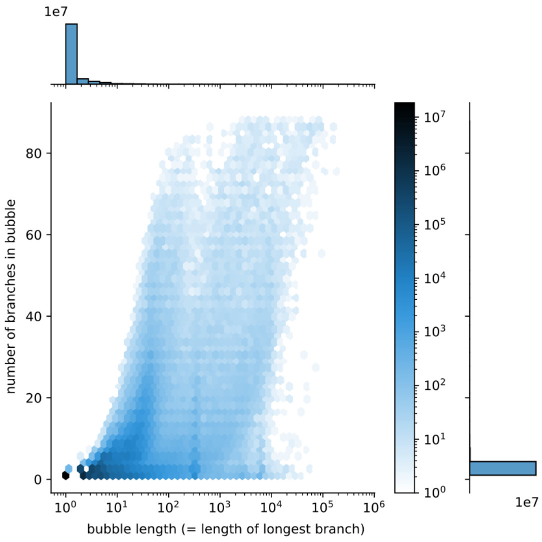

b

Allele type	# alleles in bi-allelic regions	# alleles in multi-allelic regions
**SNPs**	18,392,518	1,801,599
**Indels**	2,250,931	4,597,184
**SV ins.**	12,192	242,420
**SV del.**	4,232	52,969
**SV others**	1,296	100,700

Supplementary Figure 22 | Allele statistics. a, Each of the 88 haplotypes contained in the graph defines a path through each bubble. The plot shows the number of different paths covered by the haplotypes in a bubble as a function of the length of the bubble. Here, the length of a bubble is defined by the sequence of the longest such path. **b**, Number of variant alleles located inside of bi-allelic and multi-allelic regions of the graph. Bi-allelic regions include all bubbles with only two alternative paths, multi-allelic regions include all bubbles in which haplotypes cover more than two alternative paths through the bubble.

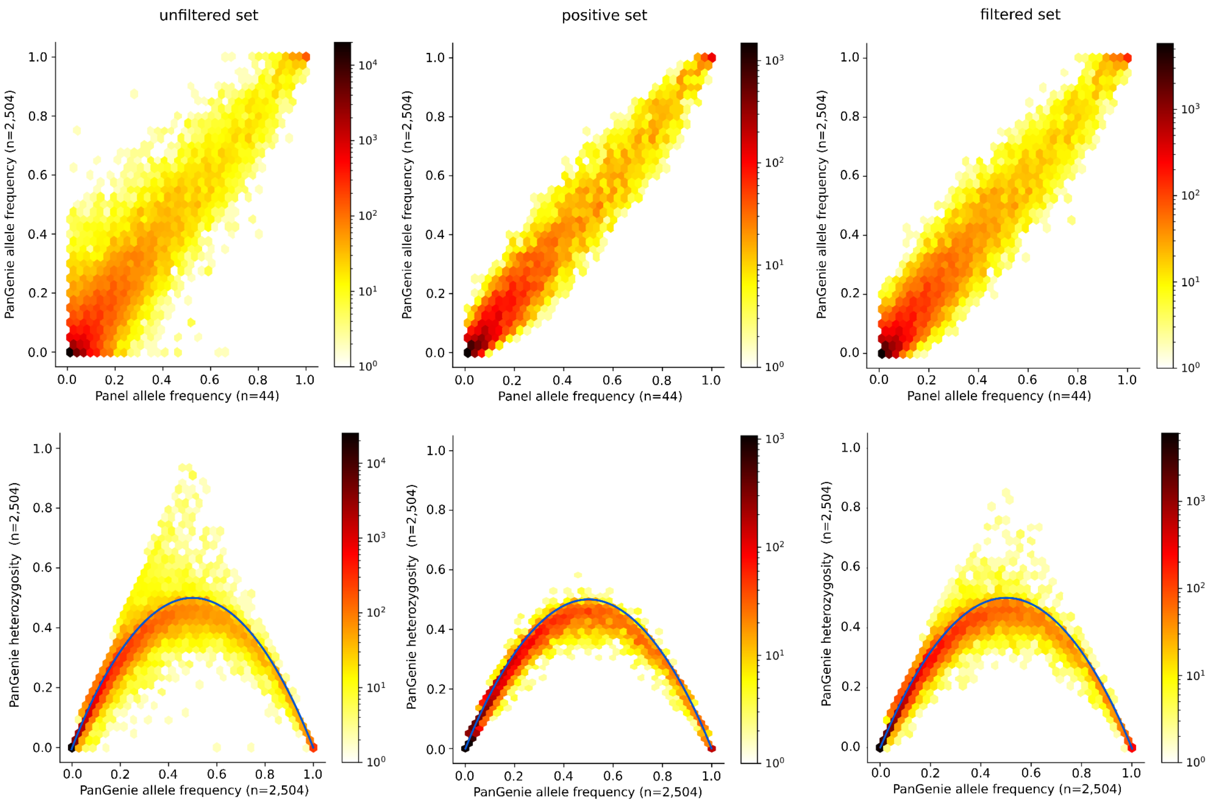

Supplementary Figure 23 | Callset statistics SV deletions. Shown are callset statistics for all SV deletion alleles (>= 50 bp) in the unfiltered set (left, n=57,201), the positive set (middle, n=13,356) and the final filtered set (right, n=28,433). The top panel compares the allele frequencies observed from the PanGenie genotypes for all 2,504 unrelated 1000 Genomes samples to the allele frequencies observed across all 44 assembly samples from the MC-based VCFs. The lower panel compares the heterozygosity across the PanGenie genotypes for all 2,504 unrelated samples to the PanGenie allele frequencies. The blue line indicates the expected relationship based on Hardy-Weinberg equilibrium.

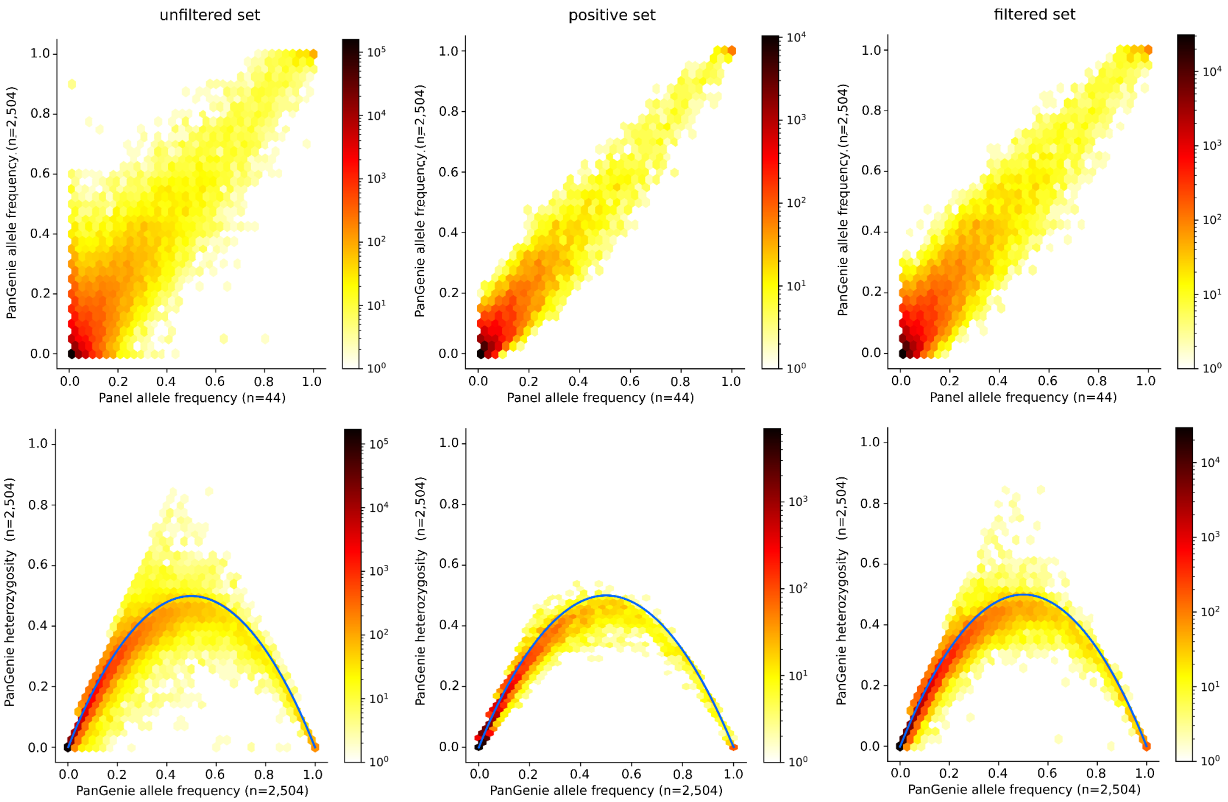

Supplementary Figure 24 | Callset statistics SV insertions. Shown are callset statistics for all SV insertion alleles (>= 50 bp) in the unfiltered set (left, n=254,612), the positive set (middle, n=32,431) and the final filtered set (right, n=84,755). The top panel compares the allele frequencies observed from the PanGenie genotypes for all 2,504 unrelated 1000 Genomes samples to the allele frequencies observed across all 44 assembly samples from the MC-based VCFs. The lower panel compares the heterozygosity across the PanGenie genotypes for all 2,504 unrelated samples to the PanGenie allele frequencies. The blue line indicates the expected relationship based on Hardy-Weinberg equilibrium.

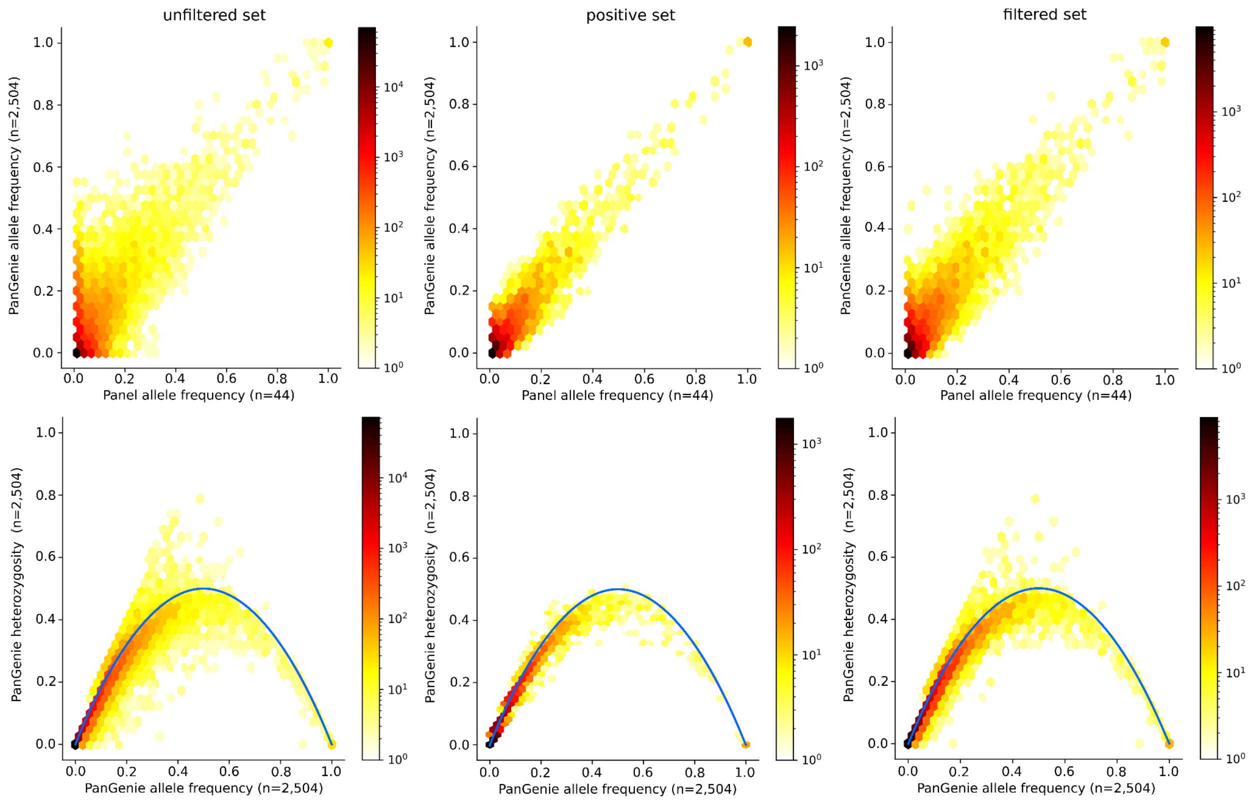

Supplementary Figure 25 | Callset statistics SV others. Shown are callset statistics for all SV alleles that are neither a clean insertion nor a clean deletion (>= 50 bp) in the unfiltered set (left, n=101,996), the positive set (middle, n=8,334) and the final filtered set (right, n=32,431). The top panel compares the allele frequencies observed from the PanGenie genotypes for all 2,504 unrelated 1000 Genomes samples to the allele frequencies observed across all 44 assembly samples from the MC-based VCFs. The lower panel compares the heterozygosity across the PanGenie genotypes for all 2,504 unrelated samples to the PanGenie allele frequencies. The blue line indicates the expected relationship based on Hardy-Weinberg equilibrium.

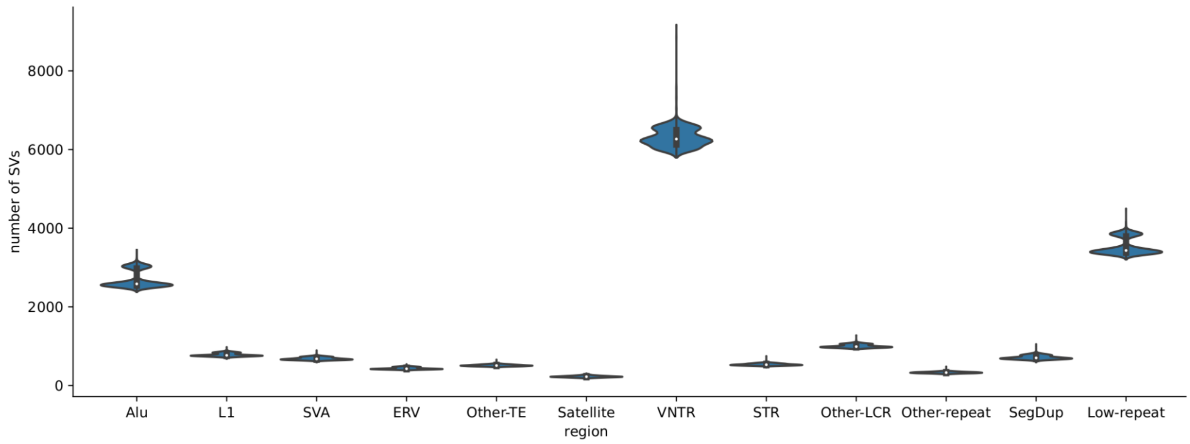

Supplementary Figure 26 | Shown are the number of SVs present (genotype 0/1 or 1/1) in each of the 3,202 1000 Genomes Project samples in the filtered HPRC genotypes (PanGenie) after merging similar alleles (n=100,442 SVs). Repeat annotations are based on the MC graph. In the box plots, lower and upper limits represent the first and third quartiles of the data, the white dots represent the median and the black lines mark minima and maxima of the data points.

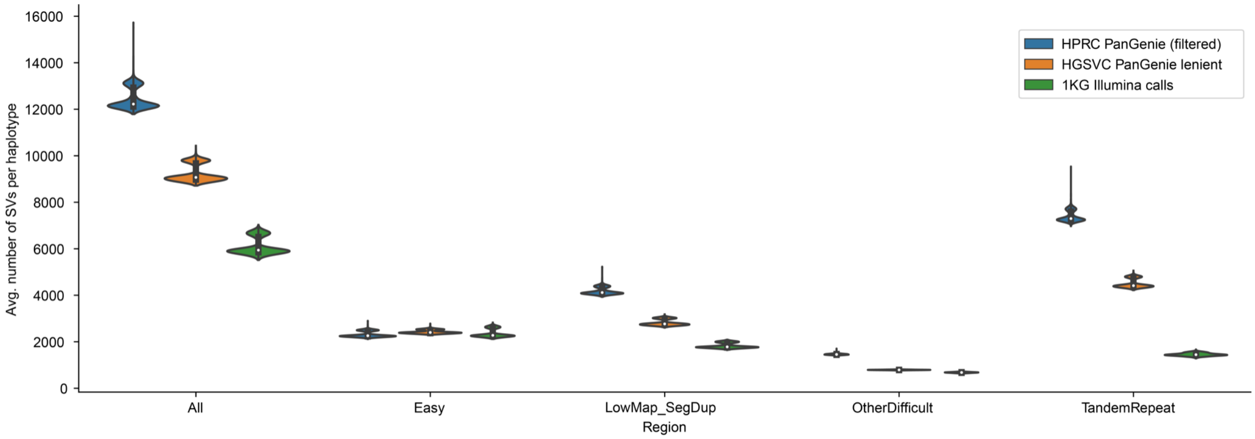

Supplementary Figure 27 | Shown are the average numbers of SVs present on each haplotype in each of the 3,202 1000 Genomes Project samples in the filtered HPRC genotypes (PanGenie) after merging similar alleles (n=100,442 SVs), the HGSVC lenient set (n=52,659 SVs) and the 1KG Illumina calls (n=172,968 SVs) in the GIAB regions. In the box plots, lower and upper limits represent the first and third quartiles of the data, the white dots represent the median and the black lines mark minima and maxima of the data points.

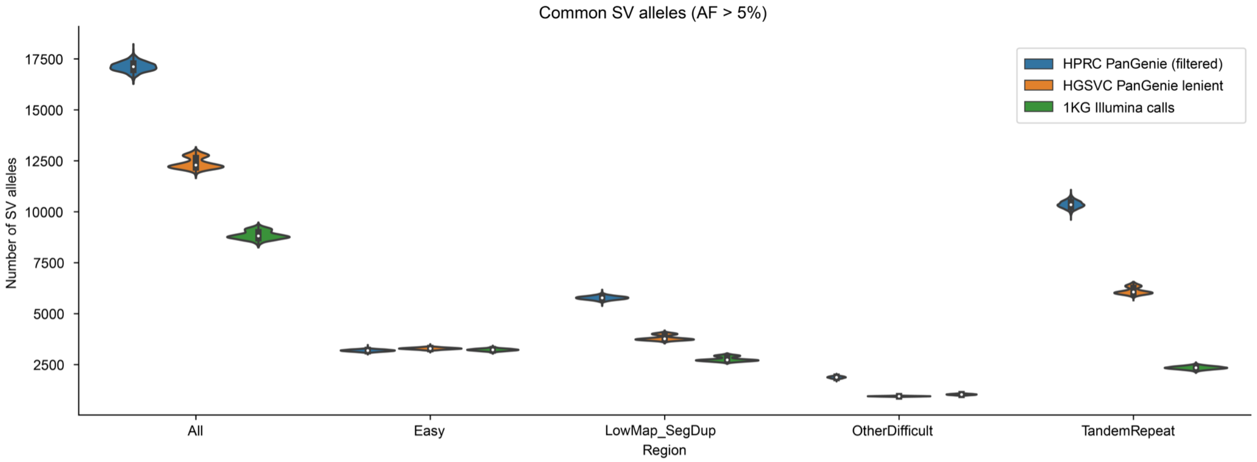

Supplementary Figure 28 | Shown are the number of common SVs (allele frequency above 5%) present (genotype 0/1 or 1/1) in each of the 3,202 1000 Genomes Project samples in the filtered HPRC genotypes (PanGenie) after merging similar alleles (n=44,180 SVs), the HGSVC lenient set (n=26,468 SVs) and the 1KG Illumina calls (n=19,304 SVs) in the GIAB regions. In the box plots, lower and upper limits represent the first and third quartiles of the data, the white dots represent the median and the black lines mark minima and maxima of the data points.

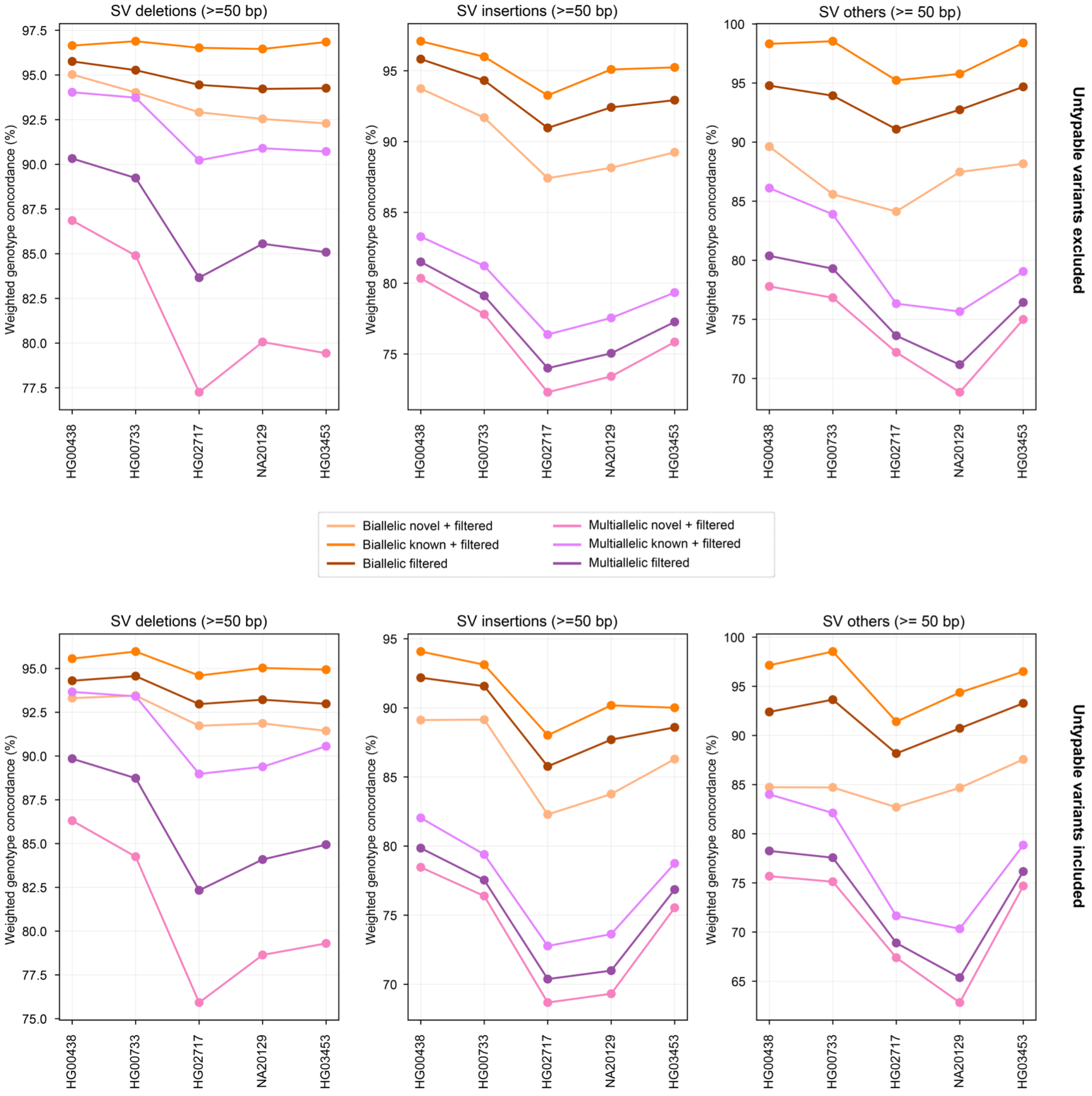

Supplementary Figure 29 | Leave-one-out experiment for novel variants. A leave-one-out experiment was conducted by repeatedly removing one of the assembly-samples from the panel VCF and genotyping it based on the remaining samples. Plots show the resulting weighted genotype concordances for variants in our filtered PanGenie set. The novel variants include only SVs not contained in the 1KG Illumina set, the known variants include only variants contained in these Illumina calls. Weighted genotype concordances are stratified by graph complexity: biallelic regions of the MC graph include only bubbles with two branches, and multiallelic regions include all bubbles with > 2 different alternative paths defined by the 88 haplotypes. The top panel excludes variants that are unique to the left-out sample and thus not typable by any re-genotyping method. Additionally, we plotted the results including untypables (bottom panel).

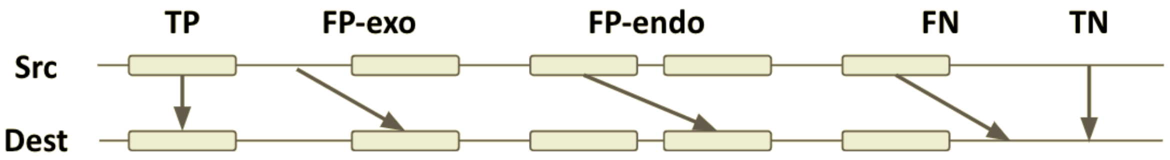

Supplementary Figure 30 | Definition of metrics for evaluating read mapping in VNTR regions. Gray boxes indicate VNTR regions. Src, source; Dest, destination; TP, true positive; FP-exo, exogenous false positive; FP-endo, endogenous false positive; FN, false negative; TN, true negative.

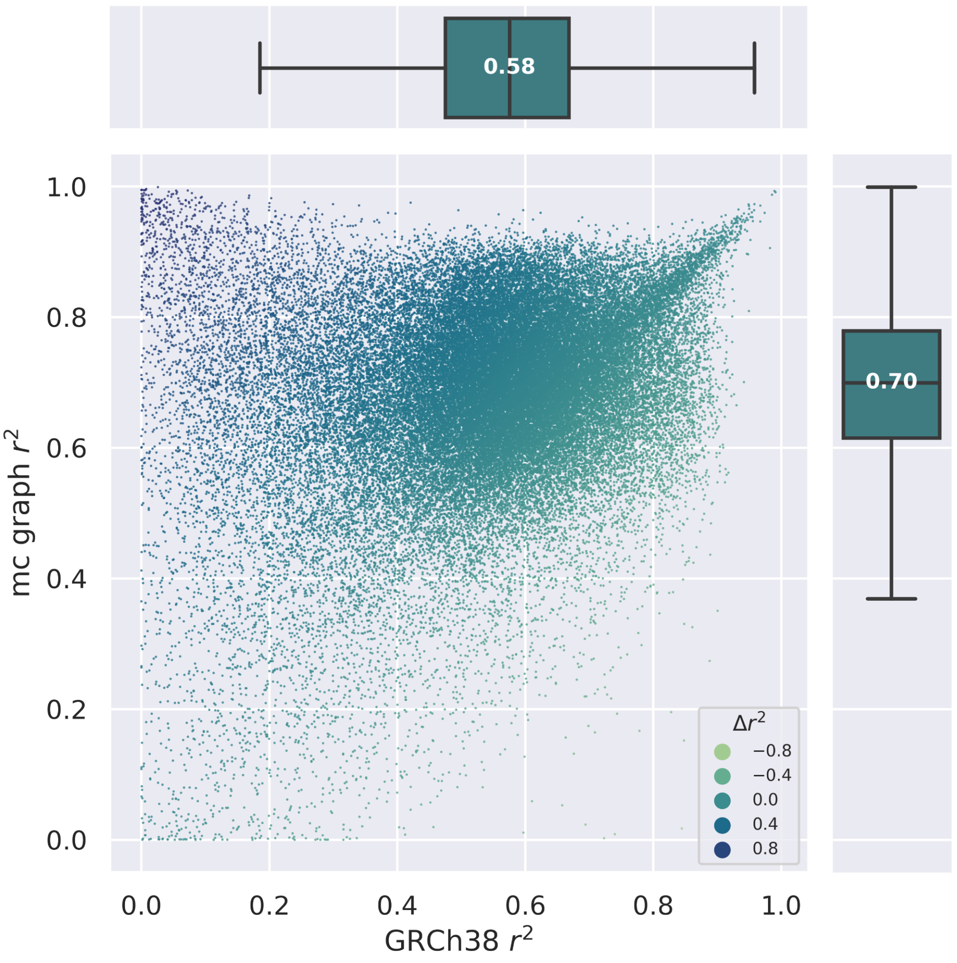

Supplementary Figure 31 | Estimating VNTR length variation from read depths. For each VNTR locus (n=60,386 VNTR loci), an r^2 was computed by regressing the estimated VNTR lengths for the 35 genomes against the ground truths. The x-axis represents the r^2 from read mapping to GRCh38. The y-axis represents the r^2 from read mapping to the MC graph. Δr^2 denotes the increase in the y value relative to the x value. Medians for both marginal distributions were shown. Whiskers in box plots extend 1.5 interquartile range beyond the low and the high quartiles.

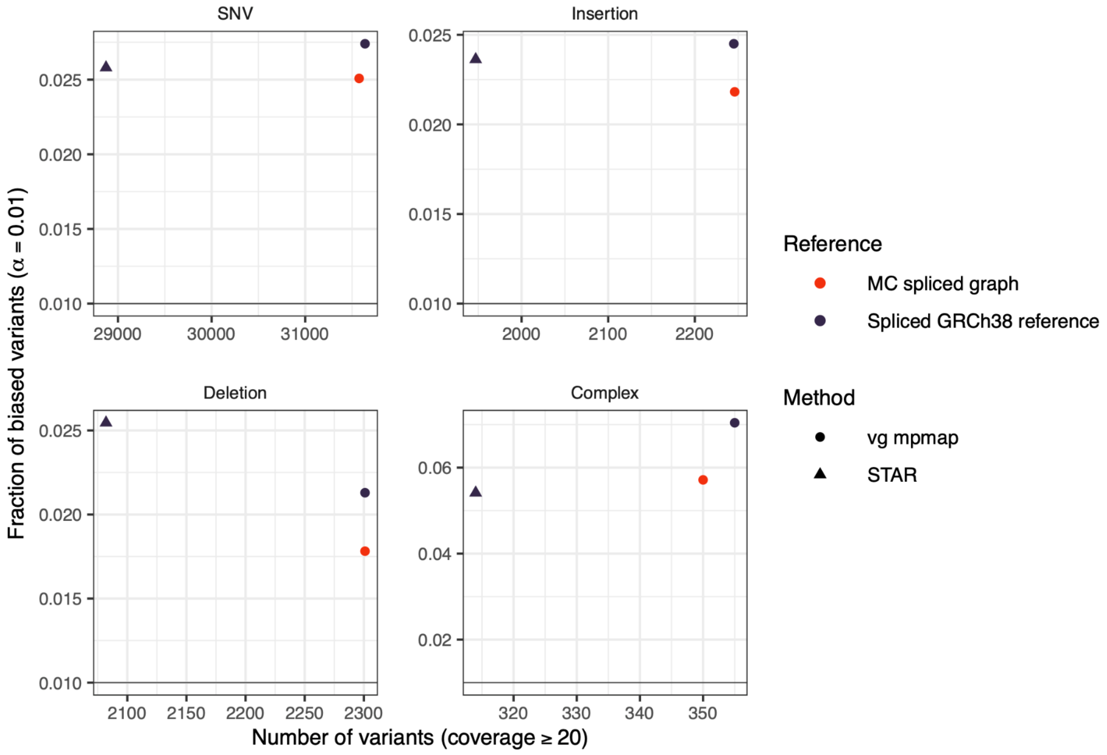

Supplementary Figure 32 | The proportion of heterozygous variants with a read coverage of at least 20 that have biased coverage between the alleles ($p \leq 0.01$, binomial test) plotted against the number of sites. Reads were simulated with no allelic bias. Reads were mapped to the GRCh38 reference with STAR and vg mpmap, and to the HPRC MC graph with only vg mpmap. Variant sites were identified with vg deconstruct and indels larger than 50 bp were excluded.

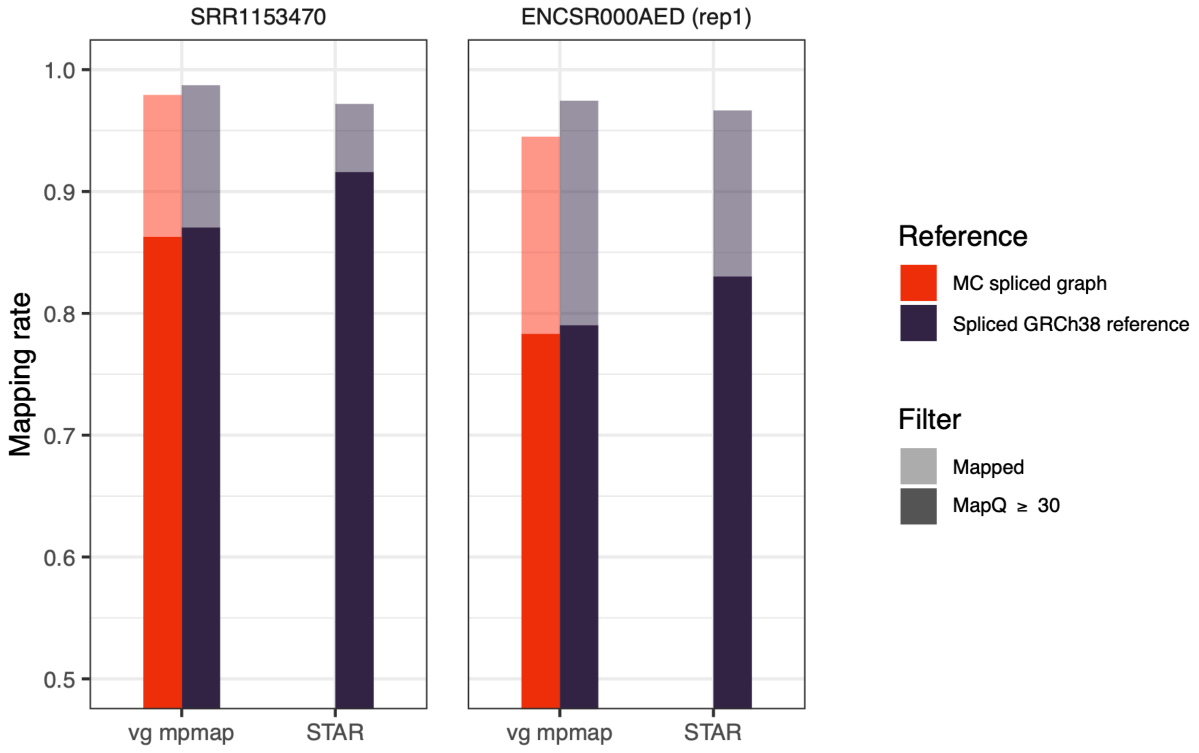

Supplementary Figure 33 | Mapping rate using two different real Illumina RNA-seq datasets (SRR1153470 and ENCSR000AED replicate 1). The solid bars show the mapping rate using a mapping quality threshold of 30. Reads were mapped with vg mpmap to the GRCh38 reference and the HPRC MC graph. Reads were mapped with STAR to only the GRCh38 reference.

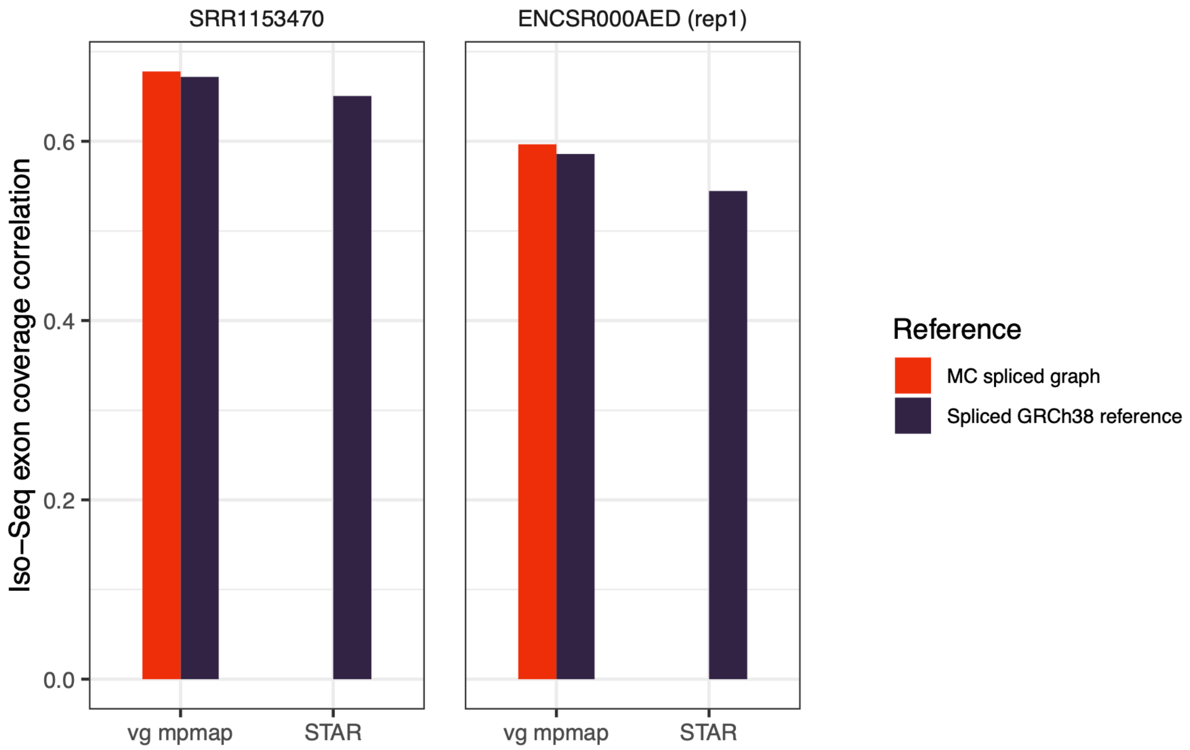

Supplementary Figure 34 | Pearson correlation between Illumina and Iso-Seq exon read coverage using two different real Illumina RNA-seq datasets (SRR1153470 and ENCSR000AED replicate 1). Results are shown for vg mpmap mapping to both the GRCh38 reference and the HPRC MC graph, and for STAR mapping to the GRCh38 reference.

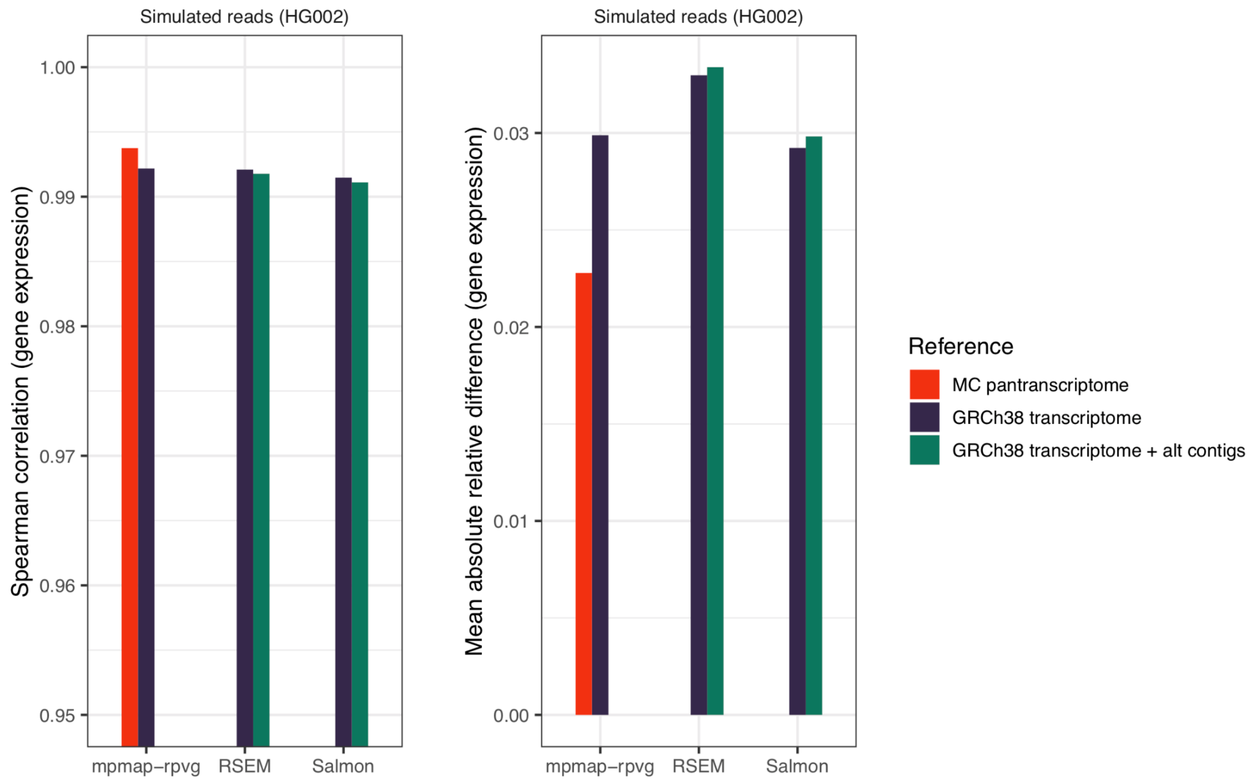

Supplementary Figure 35 | The accuracy of inferred gene expression levels based on different mappings using simulated RNA-seq data. Accuracy is measured with the Spearman correlation to the true simulated values and the mean absolute relative deviation from the true simulated values. Reads were mapped to the HPRC MC spliced graph and to the spliced GRCh38 reference using vg mpmap. Gene expression values were inferred from the vg mpmap mappings using rpvg. The MC pantranscriptome created from the CAT transcript annotations on each assembly was used as a transcript annotation for rpvg when using the MC graph mappings. For the GRCh38 mappings the GENCODE (v38) transcriptome was used. RSEM and Salmon were provided the GENCODE (v38) transcriptome, both with and without transcripts on alternative contigs included. For Salmon the GRCh38 reference was used as a decoy.

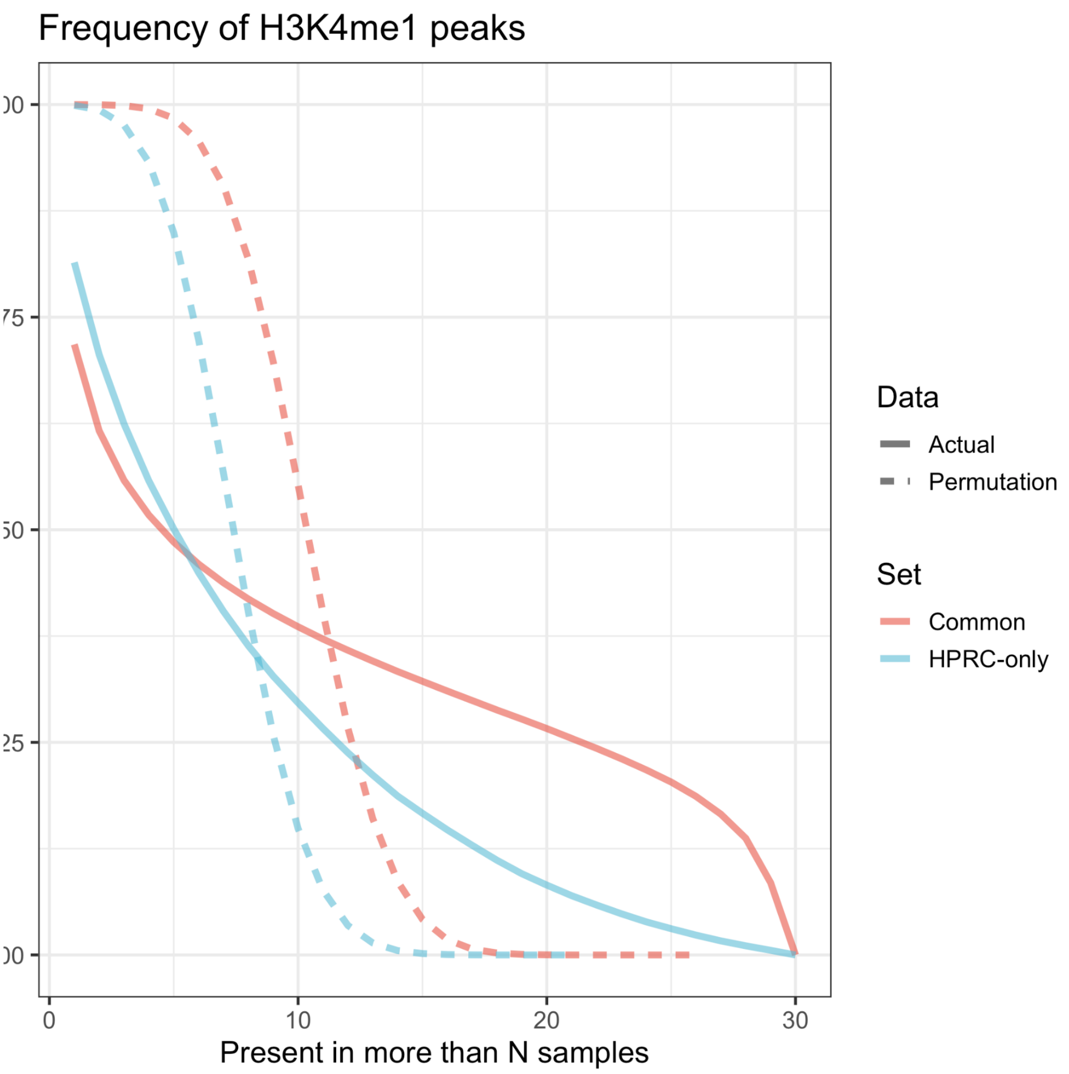

Supplementary Figure 36 | Inverse cumulative distributions describing the frequency of peaks that are found with both HPRC and GRCh38 references (common), peaks that are found only with HPRC (HPRC-only). Dashed lines represent the distributions that are expected by chance.

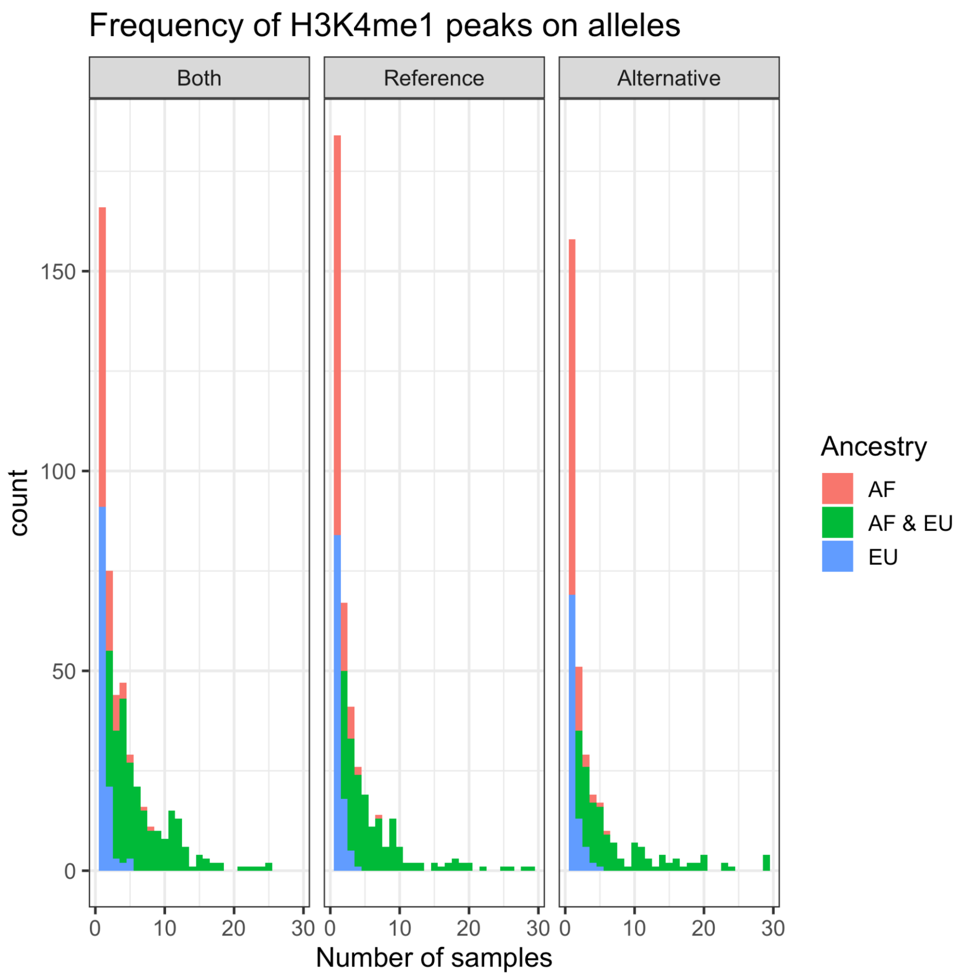

Supplementary Figure 37 | Number of samples in which H3K4me1 peaks were assigned to the SV allele, the reference allele, or both alleles. SVs with peaks are stratified into those that are observed only in African-ancestry genomes, only European-ancestry genomes, or both ancestries.

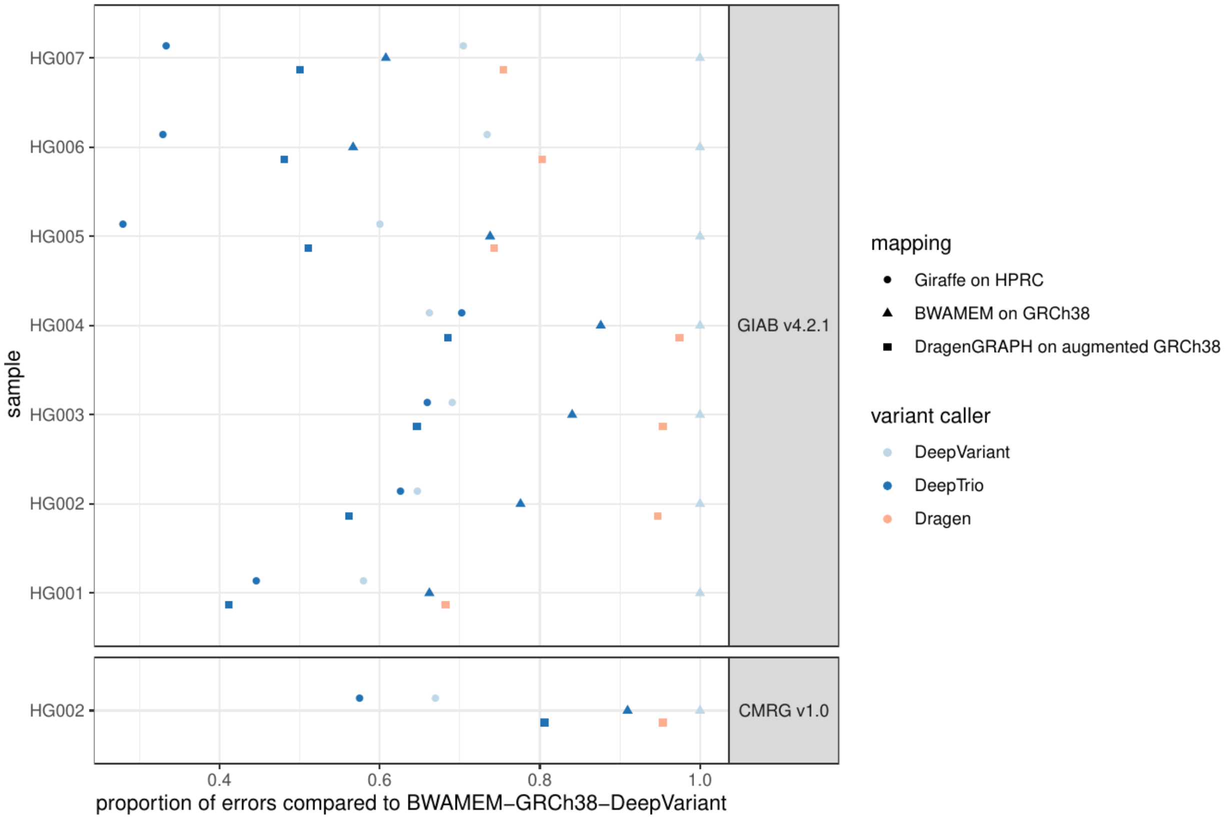

Supplementary Figure 38 | Evaluation of the SNPs and indels calls by different variant callers (colors) from different mapping approaches (shape). The x-axis represents the proportion of false positive and false negative errors compared to the BWAMEM-GRCh38-DeepVariant approach, when comparing the calls with two truth sets (horizontal panels). The “augmented GRCh38” used by the DragenGRAPH mapper (square points) corresponds to the GRCh38 genome reference plus about 900,000 known population haplotype blocks. Mapping to the HPRC pangenome with Giraffe and calling variants with DeepVariant (light blue circle) resulted in a reduction of errors of 34%, on average across samples, compared to mapping reads to the linear reference with BWA-MEM.

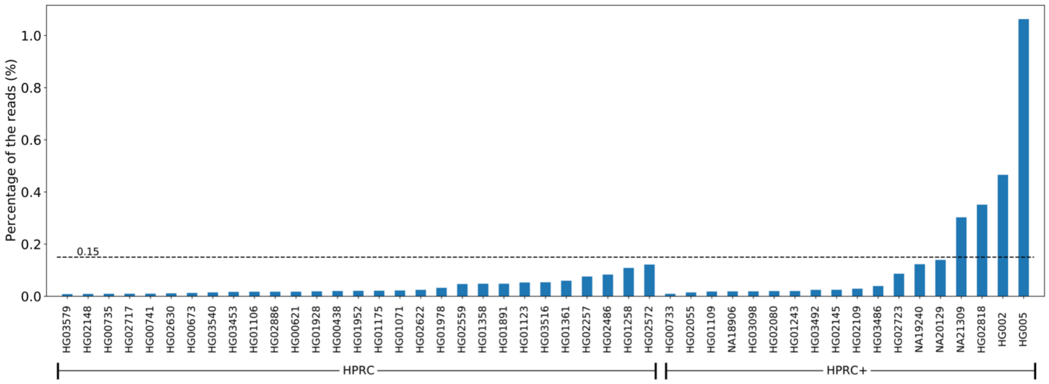

Supplementary Figure 39 | Adapter contamination percentages for 47 HPRC samples. 43 samples (out of 47) had less than 0.15% adapter contamination and HG005 had the highest percentage, about 1%.

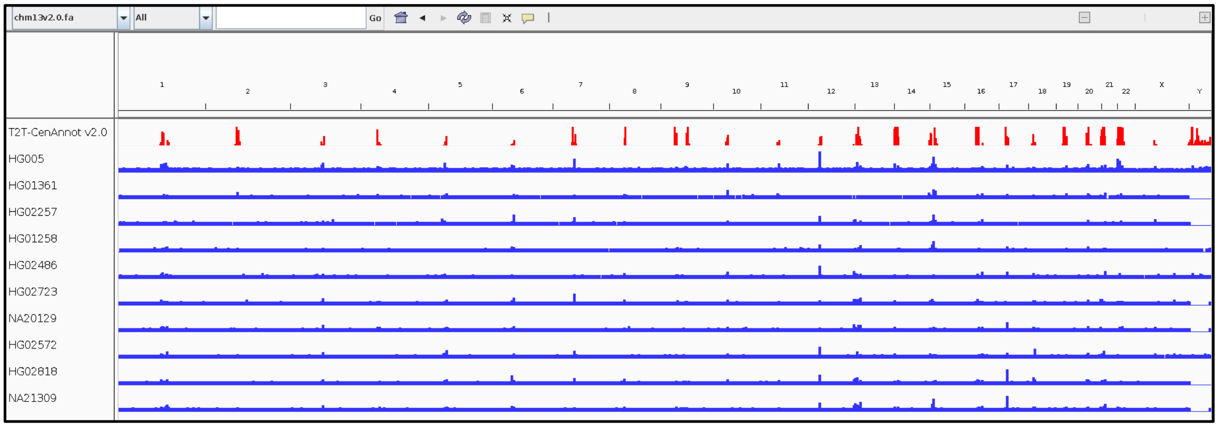

Supplementary Figure 40 | Adapter-contained reads aligned to the T2T-CHM13v2.0 reference. The top red track shows the pri/centromeric regions of the reference. The blue tracks show the coverage of the adapter-contained reads along the whole genome. The 10 samples included here are among the samples with the highest adapter contamination. For most regions the depth of coverage is at most 1 read and the reads are uniformly distributed along the genome. The coverage may rise up to 6 reads for some centromeric regions (e.g. HOR in Chr 12).

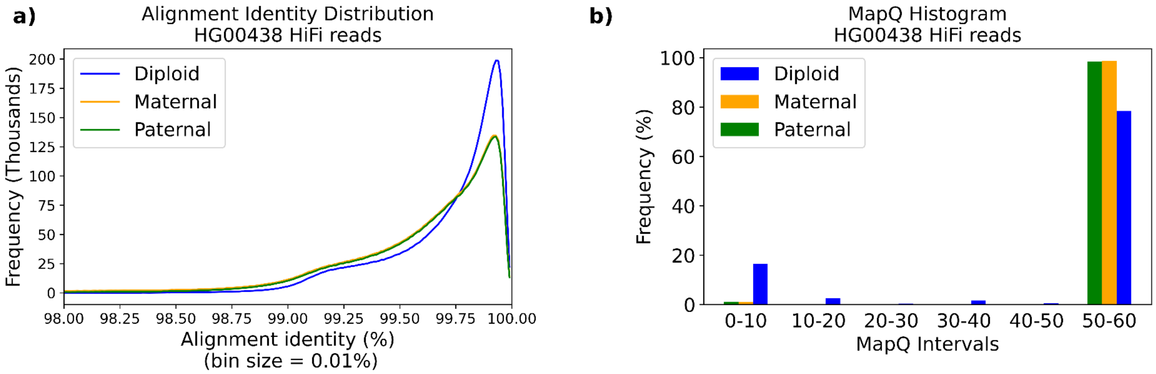

Supplementary Figure 41 | HiFi alignment identities and MapQs. We aligned HiFi reads to each paternal and maternal assemblies separately and also to both haplotypes at the same time. Panel **a** shows how the alignment identities became more skewed toward 100% when we used both haplotypes as the reference. On the other hand, the MAPQs near to zero became more frequent (blue bars in panel **b**), which is expected because of the ambiguity in mapping to the homozygous regions. Therefore, we cannot rely on MAPQ to exclude

unreliable alignments.

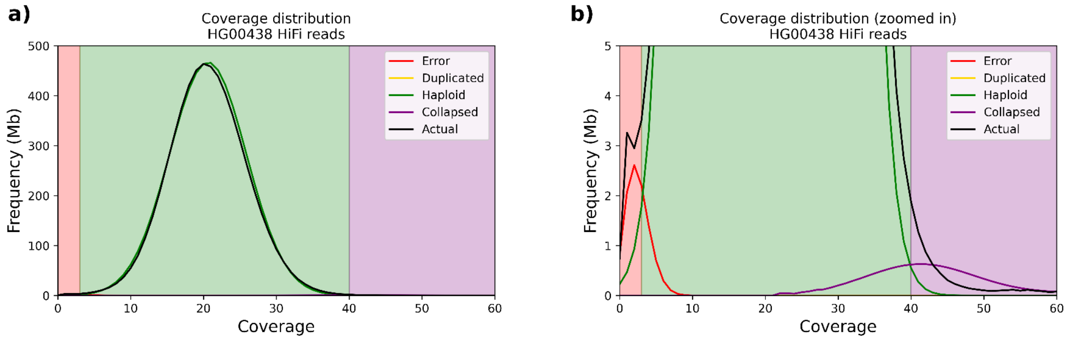

Supplementary Figure 42 | HiFi coverage distribution and fitting mixture model for HG00438. Both panels **a** and **b** show the coverage distribution of the HG00438 diploid assembly (black line). The colored lines are showing the components inferred from the mixture model but only the haploid component is visible in panel **a** since it is the dominant component as expected. To view the lower components (collapsed and erroneous), panel **b** is zoomed on the lower frequencies. Each panel is colored based on the most probable component for each coverage value.

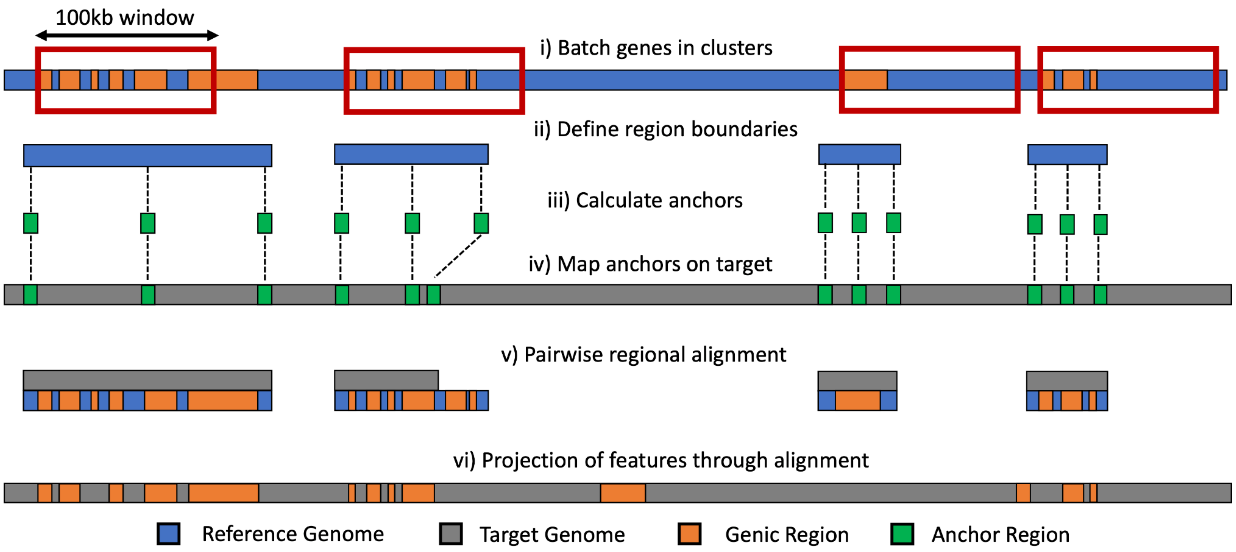

Supplementary Figure 43 | Primary mapping phase of Ensembl annotation pipeline. A sliding 100-kb window is used to find clusters of closely spaced genes. The region to map is then defined by the boundaries of the most 5’ and 3’ genes that overlap the current window, including the 5-kb flanking region on either side. 10-kb anchors are calculated at the edges of

the region and at the midpoint, and then mapped to the target. The most likely region or regions in the target are then identified and a pairwise alignment occurs between source and target regions. Using the alignment, exons are projected through the alignment and transcripts and genes are then reconstructed in the target. Once the projection process is complete, the window moves on to the next gene 3’ that does not overlap the current window position.

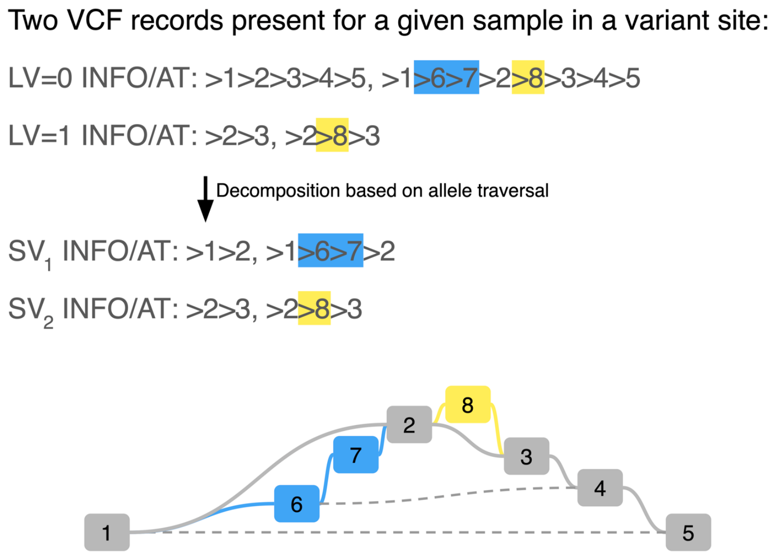

Supplementary Figure 44 | Challenge of variant representation. This example shows two variants for a given sample residing in a site. However, one of the variants (allele traversal: >1>6>7>2) cannot be further decomposed into a separate VCF record using vg deconstruct. To solve this issue, a decomposition method based on allele traversal needs to be applied.

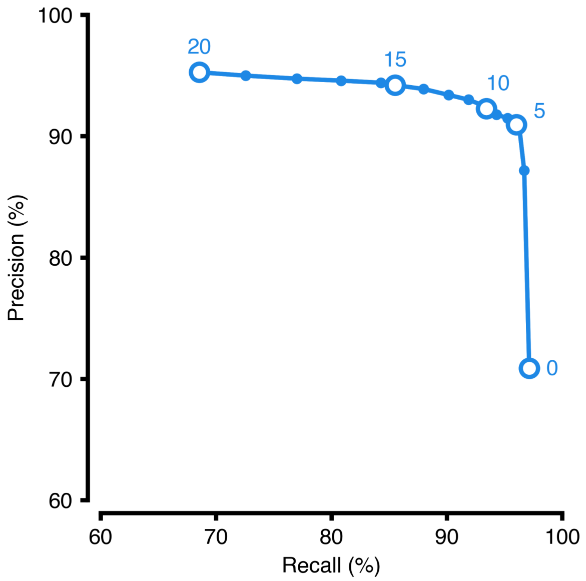

Supplementary Figure 45 | Precision-recall curve across various quality scores. For HG002, SVIM call set was compared with the GIAB v0.6 Tier 1 SV benchmark set to calculate performance metrics at different quality scores. A threshold was determined according to the precision-recall curve.

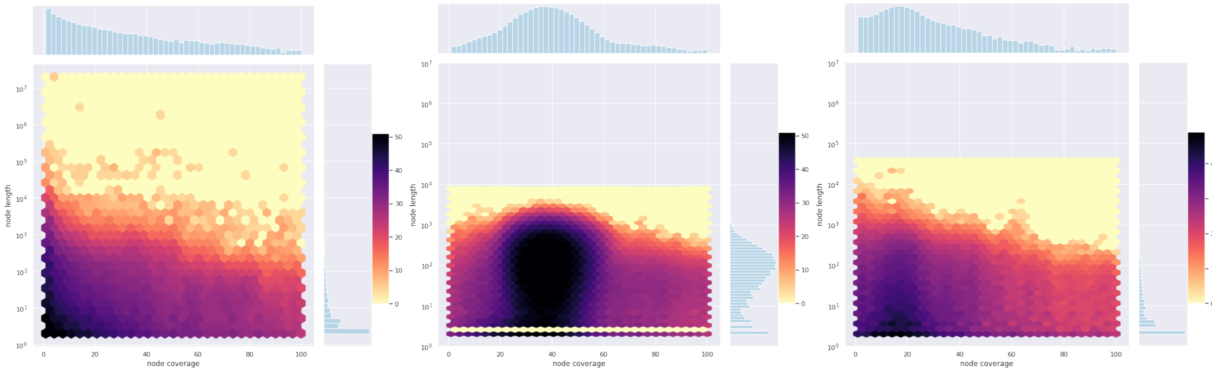

Supplementary Figure 46 | ONT read alignments against the MC graph. Node coverage of aligned reads in the graph illustrated by sample HG00438. Node length as a function of coverage for off-path, homozygous, and heterozygous nodes (left-to-right). The median coverage of homozygous nodes in the MC graph is twice (2.1) as high as of heterozygous nodes and ranges between 9- and 39-fold, whereas off-path nodes received between 1- and 3-fold median coverage. The partitions not only exhibit distinct coverage profiles, but also distinct node length distributions. In this sample, 95% of off-path nodes do not exceed 6 bases as compared to heterozygous nodes (84 bases) and homozygous nodes (299 bases), thus each partition inhabiting almost 3 distinct orders of magnitude.

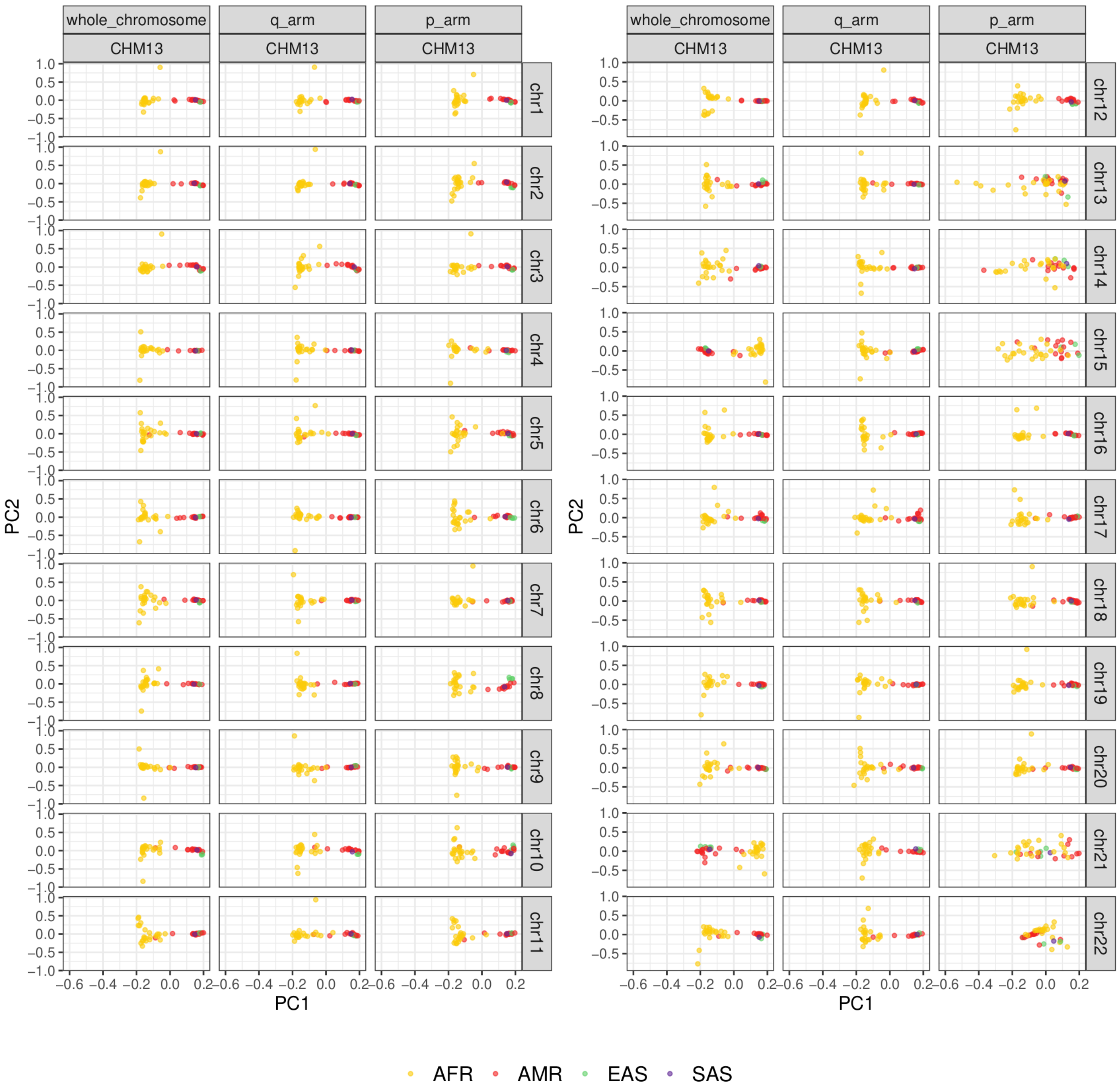

Supplementary Figure 47 | PCAs of SNPs of specified subsets of the PGGB graph. We show the first two components of PCAs derived from whole-chromosomes for CHM13 and GRCh38-based VCFs in column 1 and 2, and q-arm and p-arm specific SNPs relative to CHM13 in columns 3 and 4. We observe the same pattern of samples relative to the first two PCs in each subdivision except for the p-arms of the acrocentrics (rightmost plots for chr13, chr14, chr15, chr21, and chr22).

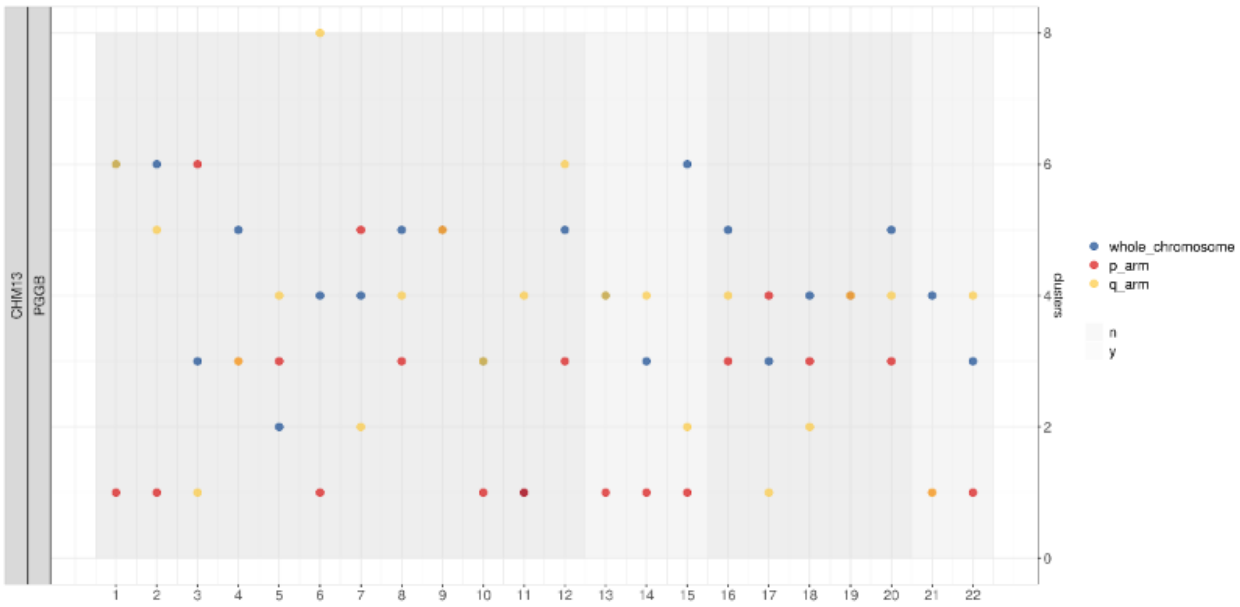

Supplementary Figure 48 | Using VCF files produced from the PGGB graphs relative to CHM13, we establish a number of PCA clusters for SNPs, considering SNPs in the whole chromosome (blue), p-arm (red), or q-arm (yellow). Transparency is used to mitigate overplotting. Acrocentric chromosomes are highlighted with a lighter background color. We observe a reduced number of clusters on the p-arms (red) of the acrocentrics.

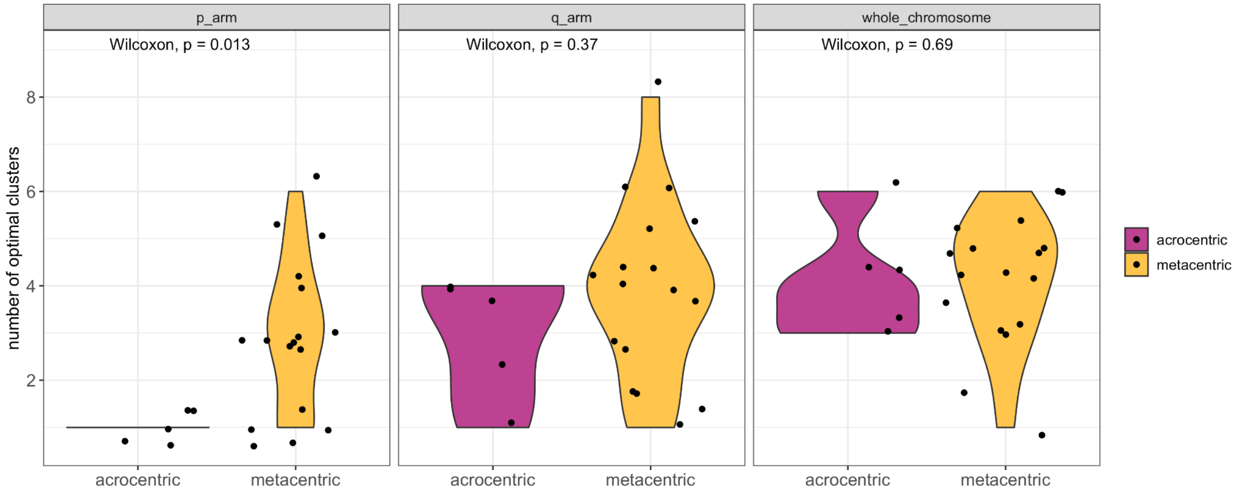

Supplementary Figure 49 | We measure the number of clusters per chromosome arms, comparing the distributions for acrocentric and metacentric whole, q-arm, and p-arms using a two-sided Wilcoxon rank-sum test. We find insignificant differences between the distributions between acrocentric and metacentric chromosomes at a chromosome scale, and in the q-arms, but a significant difference (Wilcoxon p = 0.013) in the case of acrocentric p-arms.
