# Supplementary material for: A draft human pangenome reference
Source: Nature. 2023 May 10;617(7960):312–24. doi: 10.1038/s41586-023-05896-x (PMC10172123; doi:10.1038/s41586-023-05896-x)
Supplement: Supplementary file 2 — Reporting Summary [file 41586_2023_5896_MOESM2_ESM.pdf]

nature portfolio

Corresponding author(s): Benedict Paten

Last updated by author(s): Feb 13,2023

Reporting Summary

Nature Portfolio wishes to improve the reproducibility of the work that we publish. This form provides structure for consistency and transparency in reporting. For further information on Nature Portfolio policies, see our Editorial Policies and the Editorial Policy Checklist.

Statistics

For all statistical analyses, confirm that the following items are present in the figure legend, table legend, main text, or Methods section.

n/a	Confirmed	
☐	☒	The exact sample size (n) for each experimental group/condition, given as a discrete number and unit of measurement
☐	☒	A statement on whether measurements were taken from distinct samples or whether the same sample was measured repeatedly
☐	☒	The statistical test(s) used AND whether they are one- or two-sided<br>*Only common tests should be described solely by name; describe more complex techniques in the Methods section.*
☐	☒	A description of all covariates tested
☐	☒	A description of any assumptions or corrections, such as tests of normality and adjustment for multiple comparisons
☐	☒	A full description of the statistical parameters including central tendency (e.g. means) or other basic estimates (e.g. regression coefficient) AND variation (e.g. standard deviation) or associated estimates of uncertainty (e.g. confidence intervals)
☐	☒	For null hypothesis testing, the test statistic (e.g. F, t, r) with confidence intervals, effect sizes, degrees of freedom and P value noted<br>*Give P values as exact values whenever suitable.*
☒	☐	For Bayesian analysis, information on the choice of priors and Markov chain Monte Carlo settings
☒	☐	For hierarchical and complex designs, identification of the appropriate level for tests and full reporting of outcomes
☐	☒	Estimates of effect sizes (e.g. Cohen's d, Pearson's r), indicating how they were calculated

Our web collection on statistics for biologists contains articles on many of the points above.

Software and code

Policy information about availability of computer code

Data collection: No software was used

Data analysis:
abPOA https://github.com/yangao07/abPOA
ABRA v2.23 https://github.com/mozack/abra
Bandage v0.8.1 https://rrwick.github.io/Bandage/
bcftools v1.16 https://samtools.github.io/bcftools/
BEDTools v2.28.0, v2.30.0 https://bedtools.readthedocs.io/en/latest/
Bowtie2 v2.4.5 https://github.com/BenLangmead/bowtie2
BWA-MEMv0.7.17-r1188 https://github.com/lh3/bwa
Cactus 6cd9a42 https://github.com/ComparativeGenomicsToolkit/cactus
Comparative Annotation Toolkit (CAT) eb2fc87 https://github.com/ComparativeGenomicsToolkit/Comparative-Annotation-Toolkit
danbing-tk v1.3 https://github.com/ChaissonLab/danbing-tk
DeepVariant v1.3.0 https://github.com/google/deepvariant
Dfam v3.3 https://www.dfam.org/home
Dipcall v0.3 https://github.com/lh3/dipcall
dna-brnn v0.1 https://github.com/lh3/dna-nn
Exact Tandem Repeat Finder (ETRF) fc059d5 https://github.com/lh3/etrf
Exonerate https://www.ebi.ac.uk/about/vertebrate-genomics/software/exonerate
Flagger v0.1 https://github.com/mobinasri/flagger
FreeBayes v1.2.0 https://github.com/freebayes/freebayes
GATK v3.8.1 https://gatk.broadinstitute.org/hc/en-us

gfabase v0.6.0 https://github.com/mlin/gfabase
GFAffix v0.1.3 https://github.com/marschall-lab/GFAffix
GFAtools v0.5 https://github.com/lh3/gfatools
GffRead v0.12.7 https://github.com/gpertea/gffread
gggenes v0.4.1 https://github.com/wilkox/gggenes
Graph Peak Caller v1.2.3 https://github.com/uio-bmi/graph_peak_caller
GraphAligner v1.0.13 https://github.com/maickrau/GraphAligner
hal2vg https://github.com/ComparativeGenomicsToolkit/hal2vg
Hall-lab pipeline 830260a https://github.com/hall-lab/competitive-alignment/blob/master/call_assembly_variants.wdl
halLiftover https://github.com/ComparativeGenomicsToolkit/hal
hap.py v3.15 https://github.com/Illumina/hap.py
HiFiAdapterFilt 64d1c7b https://github.com/sheinasim/HiFiAdapterFilt
hifiasm v0.14, v0.14.1 https://github.com/chhylp123/hifiasm
Illumina's Dragen platform v3.7.5 https://support.illumina.com/content/dam/illumina-support/help/
Illumina_DRAGEN_Bio_IT_Platform_v3_7_1000000141465/Content/In/Informatics/DRAGEN/GraphMapper_fDG.htm
Integrative Genome Browser (IGV) https://software.broadinstitute.org/software/igv/
Iris v1.0.4 https://github.com/mkirsche/Iris
Liftoff v1.6.3 https://github.com/agshumate/Liftoff
MAFFT https://mafft.cbrc.jp/alignment/software/
Merqury 261b085 https://github.com/marbl/merqury
meryl 6d396a0 https://github.com/marbl/meryl
Minigraph v0.14, v0.18 https://github.com/lh3/minigraph
minimap2 v2.21, v2.1, v2.2.4 https://github.com/lh3/minimap2
NCBI/RMBLAST v2.10.0 http://www.repeatmasker.org/rmblast/
odgi pangenome analysis toolkit https://github.com/pangenome/odgi
panacus 1eeb6d0 https://github.com/marschall-lab/panacus
PanGenie v1.0.0 https://github.com/eblerjana/pangenie
PanGenome Graph Builder (PGGB) v0.2.0+531f85f https://github.com/pangenome/pggb
PAV v0.9.1 https://github.com/EichlerLab/pav
PBSV v2.6.2 https://github.com/PacificBiosciences/pbsv
Pstools v0.1 https://github.com/shilpagarg/pstools
QUAST v5.0.2 https://quast.sourceforge.net/
RepeatMasker v4.1.2-p1 https://www.repeatmasker.org/
RPVG 1d91a9e https://github.com/jonassibbesen/rpvg
RSEM v1.3.3 https://github.com/deweylab/RSEM
RTG Tools v3.12.1 https://github.com/RealTimeGenomics/rtg-tools
Salmon v1.9.0 https://github.com/COMBINE-lab/salmon
Samtools v1.3.1, v1.15 https://github.com/samtools/samtools
SDUST v0.1 https://github.com/lh3/sdust
Secphase v0.1 https://github.com/mobinasri/secphase
sedef https://github.com/vpc-ccg/sedef
seqtk v1.3 https://github.com/lh3/seqtk
seqwish https://github.com/ekg/seqwish
slivar v0.2.7 https://github.com/brentp/slivar
smoothxg https://github.com/pangenome/smoothxg
Sniffles v1.0.12b https://github.com/fritzsedlazeck/Sniffles
STAR v2.7.10a https://github.com/alexdobin/STAR
SVIM v2.0.0 https://github.com/eldariont/svim
SVIM-asm v1.0.2 https://github.com/eldariont/svim-asm
svtools https://github.com/hall-lab/svtools
Tandem Repeat Finder (TRF) v4.09 https://tandem.bu.edu/trf/trfdesc.html
truvari v3.2.0, v3.1.0 https://github.com/ACEnglish/truvari
vcfbub v0.1.0 https://github.com/pangenome/vcfbub
vcflib https://github.com/vcflib/vcflib
vg, vg giraffe v1.33.0, v1.36.0, v1.37.0, v1.38.0, v1.39.0, v1.43.0, 2cea1e2, c0c4816 https://github.com/vgteam/vg
wfmash https://github.com/waveygang/wfmash
WhatsHap v1.1 https://whatshap.readthedocs.io/en/latest/
winnowmap v2.03 https://github.com/marbl/Winnowmap
Yak v0.1 https://github.com/lh3/yak

For manuscripts utilizing custom algorithms or software that are central to the research but not yet described in published literature, software must be made available to editors and reviewers. We strongly encourage code deposition in a community repository (e.g. GitHub). See the Nature Portfolio guidelines for submitting code & software for further information.

Data

Policy information about availability of data

All manuscripts must include a data availability statement. This statement should provide the following information, where applicable:

- Accession codes, unique identifiers, or web links for publicly available datasets
- A description of any restrictions on data availability
- For clinical datasets or third party data, please ensure that the statement adheres to our policy

Sequencing data, assemblies, and pangenomes produced by the HPRC are available in the AnVIL (https://anvilproject.org/) in the AnVIL_HPRC workspace. Data is also available as part of the AWS Open Data Program (https://registry.opendata.aws/) in the human-pangenomics S3 bucket (https://s3-us-west-2.amazonaws.com/human-pangenomics/index.html). In addition, data is uploaded to INSDC for long term storage and availability. Supporting information about the data (including

index files with S3 and GCP file locations) can be found in our GitHub repositories (see below).

Sequencing data for 29 selected HPRC samples from the 1KG cohort (Results) are uploaded to BioProject PRJNA701308 (https://www.ncbi.nlm.nih.gov/bioproject/PRJNA701308/). Sequencing data created by the HPRC for samples in the cohort of 18 additional samples are uploaded to BioProject PRJNA731524 (https://www.ncbi.nlm.nih.gov/bioproject/PRJNA731524). Both sets of assemblies are grouped by an Umbrella BioProject PRJNA730822. Data used in this paper has additional information available in AnVIL and in our GitHub (https://github.com/human-pangenomics/HPP_Year1_Data_Freeze_v1.0).

Assemblies along with assembly annotations, such as repeat masker and Ensembl gene annotations, can be viewed in an assembly hub in the UCSC Genome Browser (http://hprc-browser.ucsc.edu/). The data and annotations can also be accessed through the Ensembl Rapid Release Genome Browser (https://rapid.ensembl.org) and a dedicated Ensembl project page for centralized access to HPRC data (https://projects.ensembl.org/hprc/), which includes links to download the data locally. File locations for the assemblies (and select annotation files) that are stored in AWS and GCP can also be found in AnVIL or in GitHub (https://github.com/human-pangenomics/HPP_Year1_Assemblies).

Pangenomes were uploaded to the European Nucleotide Archive as analysis objects and are organized under Umbrella BioProject PRJNA850430 (https://www.ncbi.nlm.nih.gov/bioproject/PRJNA850430). File locations for pangenomes, indexes, and variant calls derived from the pangenomes can be found in AnVIL or in our GitHub (https://github.com/human-pangenomics/hpp_pangenome_resources).

Variant calls produced by the analysis performed in this paper are available in Amazon S3 (https://s3-us-west-2.amazonaws.com/human-pangenomics/index.html?prefix=publications/PANGENOME_2022/) or where indicated in the relevant sections below.

PanGenie genotypes produced for the 1KG samples based on the MC graph are available at: https://doi.org/10.5281/zenodo.6797328.

Human research participants

Policy information about studies involving human research participants and Sex and Gender in Research.

Reporting on sex and gender	This study used cell lines derived from both male (19) and female (28) donors (47 in total). For 31 samples, we performed whole genome sequencing in this study and for all other samples, sequencing data was taken from other studies referenced in this manuscript. Lymphoblastoid cell lines (LCLs) used for sequencing from the 1000 Genomes Project collection were obtained from the NHGRI Sample Repository for Human Genetic Research and HG002 (GM24385) and HG005 (GM24631) LCLs were obtained from the NIGMS Human Genetic Cell Repository at the Coriell Institute for Medical Research. Therefore, this study is exempt from human subjects research since the proposed work involves the collection or study of data or specimens that are already publicly available.
Population characteristics	See above
Recruitment	See above
Ethics oversight	*Identify the organization(s) that approved the study protocol.*

Note that full information on the approval of the study protocol must also be provided in the manuscript.

Field-specific reporting

Please select the one below that is the best fit for your research. If you are not sure, read the appropriate sections before making your selection.

☒ Life sciences ☐ Behavioural & social sciences ☐ Ecological, evolutionary & environmental sciences

For a reference copy of the document with all sections, see nature.com/documents/nr-reporting-summary-flat.pdf

Life sciences study design

All studies must disclose on these points even when the disclosure is negative.

Sample size	Decided with respect to HPRC's year1-2 funding
Data exclusions	Candidates without both parental data, or optimal passage biosamples, were excluded.
Replication	Experiments were computational so replication is not applicable. For reproducibility all of the data sets and codes/workflows are publicly available. The corresponding links are mentioned in data availability and code availability statements.
Randomization	The samples were not allocated into different experimental groups.
Blinding	Blinding is not relevant to this study since the samples in this study were selected solely based on the genetic diversity. Comparison between two or more experimental groups was not the purpose of the study so blinding was not necessary.

Behavioural & social sciences study design

All studies must disclose on these points even when the disclosure is negative.

Study description	*Briefly describe the study type including whether data are quantitative, qualitative, or mixed-methods (e.g. qualitative cross-sectional, quantitative experimental, mixed-methods case study).*
Research sample	*State the research sample (e.g. Harvard university undergraduates, villagers in rural India) and provide relevant demographic information (e.g. age, sex) and indicate whether the sample is representative. Provide a rationale for the study sample chosen. For studies involving existing datasets, please describe the dataset and source.*
Sampling strategy	*Describe the sampling procedure (e.g. random, snowball, stratified, convenience). Describe the statistical methods that were used to predetermine sample size OR if no sample-size calculation was performed, describe how sample sizes were chosen and provide a rationale for why these sample sizes are sufficient. For qualitative data, please indicate whether data saturation was considered, and what criteria were used to decide that no further sampling was needed.*
Data collection	*Provide details about the data collection procedure, including the instruments or devices used to record the data (e.g. pen and paper, computer, eye tracker, video or audio equipment) whether anyone was present besides the participant(s) and the researcher, and whether the researcher was blind to experimental condition and/or the study hypothesis during data collection.*
Timing	*Indicate the start and stop dates of data collection. If there is a gap between collection periods, state the dates for each sample cohort.*
Data exclusions	*If no data were excluded from the analyses, state so OR if data were excluded, provide the exact number of exclusions and the rationale behind them, indicating whether exclusion criteria were pre-established.*
Non-participation	*State how many participants dropped out/declined participation and the reason(s) given OR provide response rate OR state that no participants dropped out/declined participation.*
Randomization	*If participants were not allocated into experimental groups, state so OR describe how participants were allocated to groups, and if allocation was not random, describe how covariates were controlled.*

Ecological, evolutionary & environmental sciences study design

All studies must disclose on these points even when the disclosure is negative.

Study description	*Briefly describe the study. For quantitative data include treatment factors and interactions, design structure (e.g. factorial, nested, hierarchical), nature and number of experimental units and replicates.*
Research sample	*Describe the research sample (e.g. a group of tagged Passer domesticus, all Stenocereus thurberi within Organ Pipe Cactus National Monument), and provide a rationale for the sample choice. When relevant, describe the organism taxa, source, sex, age range and any manipulations. State what population the sample is meant to represent when applicable. For studies involving existing datasets, describe the data and its source.*
Sampling strategy	*Note the sampling procedure. Describe the statistical methods that were used to predetermine sample size OR if no sample-size calculation was performed, describe how sample sizes were chosen and provide a rationale for why these sample sizes are sufficient.*
Data collection	*Describe the data collection procedure, including who recorded the data and how.*
Timing and spatial scale	*Indicate the start and stop dates of data collection, noting the frequency and periodicity of sampling and providing a rationale for these choices. If there is a gap between collection periods, state the dates for each sample cohort. Specify the spatial scale from which the data are taken*
Data exclusions	*If no data were excluded from the analyses, state so OR if data were excluded, describe the exclusions and the rationale behind them, indicating whether exclusion criteria were pre-established.*
Reproducibility	*Describe the measures taken to verify the reproducibility of experimental findings. For each experiment, note whether any attempts to repeat the experiment failed OR state that all attempts to repeat the experiment were successful.*
Randomization	*Describe how samples/organisms/participants were allocated into groups. If allocation was not random, describe how covariates were controlled. If this is not relevant to your study, explain why.*
Blinding	*Describe the extent of blinding used during data acquisition and analysis. If blinding was not possible, describe why OR explain why blinding was not relevant to your study.*

Did the study involve field work? ☐ Yes ☐ No

Field work, collection and transport

Field conditions	*Describe the study conditions for field work, providing relevant parameters (e.g. temperature, rainfall).*
Location	*State the location of the sampling or experiment, providing relevant parameters (e.g. latitude and longitude, elevation, water depth).*
Access & import/export	*Describe the efforts you have made to access habitats and to collect and import/export your samples in a responsible manner and in compliance with local, national and international laws, noting any permits that were obtained (give the name of the issuing authority, the date of issue, and any identifying information).*
Disturbance	*Describe any disturbance caused by the study and how it was minimized.*

Reporting for specific materials, systems and methods

We require information from authors about some types of materials, experimental systems and methods used in many studies. Here, indicate whether each material, system or method listed is relevant to your study. If you are not sure if a list item applies to your research, read the appropriate section before selecting a response.

Materials & experimental systems

n/a	Involved in the study	
☒	☐	Antibodies
☐	☒	Eukaryotic cell lines
☒	☐	Palaeontology and archaeology
☒	☐	Animals and other organisms
☒	☐	Clinical data
☒	☐	Dual use research of concern

Methods

n/a	Involved in the study	
☐	☒	ChIP-seq
☒	☐	Flow cytometry
☒	☐	MRI-based neuroimaging

Antibodies

Antibodies used	*Describe all antibodies used in the study; as applicable, provide supplier name, catalog number, clone name, and lot number.*
Validation	*Describe the validation of each primary antibody for the species and application, noting any validation statements on the manufacturer's website, relevant citations, antibody profiles in online databases, or data provided in the manuscript.*

Eukaryotic cell lines

Policy information about cell lines and Sex and Gender in Research

Cell line source(s)

Lymphoblastoid cell lines used for sequencing from the 1KG collection were obtained from the NHGRI Sample Repository for Human Genetic Research at the Coriell Institute for Medical Research.
HG002 (GM24385) and HG005 (GM24631) lymphoblastoid cell lines were obtained from the NIGMS Human Genetic Cell Repository at the Coriell Institute for Medical Research.

Sample Cohort Sex

HG01123 HPRC female
HG01258 HPRC male
HG01358 HPRC male
HG01361 HPRC female
HG01891 HPRC female
HG02257 HPRC female
HG02486 HPRC male
HG02559 HPRC female
HG02572 HPRC male
HG03516 HPRC female
HG00438 HPRC female
HG00621 HPRC male
HG00673 HPRC male
HG00735 HPRC female
HG00741 HPRC female
HG01071 HPRC female
HG01106 HPRC male
HG01175 HPRC female
HG01928 HPRC male

HG01952 HPRC male
HG01978 HPRC female
HG02148 HPRC female
HG02622 HPRC female
HG02630 HPRC female
HG02717 HPRC male
HG02886 HPRC female
HG03453 HPRC female
HG03540 HPRC female
HG03579 HPRC male
HG002 HPRC_PLUS male
HG005 HPRC_PLUS male

Authentication

This study involved the use of authenticated cell lines that are publicly available from the NHGRI Sample Repository for Human Genetic Research and the NIGMS Human Genetic Cell Repository at the Coriell Institute for Medical Research. Cell line identity was confirmed using a multiplex PCR assay for six autosomal microsatellite markers.

Mycoplasma contamination

All cell lines tested negative for mycoplasma contamination.

Commonly misidentified lines (See ICLAC register)

No misidentified cell line was used in this study.

Palaeontology and Archaeology

Specimen provenance

Provide provenance information for specimens and describe permits that were obtained for the work (including the name of the issuing authority, the date of issue, and any identifying information). Permits should encompass collection and, where applicable, export.

Specimen deposition

Indicate where the specimens have been deposited to permit free access by other researchers.

Dating methods

If new dates are provided, describe how they were obtained (e.g. collection, storage, sample pretreatment and measurement), where they were obtained (i.e. lab name), the calibration program and the protocol for quality assurance OR state that no new dates are provided.

☐ Tick this box to confirm that the raw and calibrated dates are available in the paper or in Supplementary Information.

Ethics oversight

Identify the organization(s) that approved or provided guidance on the study protocol, OR state that no ethical approval or guidance was required and explain why not.

Note that full information on the approval of the study protocol must also be provided in the manuscript.

Animals and other research organisms

Policy information about studies involving animals; ARRIVE guidelines recommended for reporting animal research, and Sex and Gender in Research

Laboratory animals

For laboratory animals, report species, strain and age OR state that the study did not involve laboratory animals.

Wild animals

Provide details on animals observed in or captured in the field; report species and age where possible. Describe how animals were caught and transported and what happened to captive animals after the study (if killed, explain why and describe method; if released, say where and when) OR state that the study did not involve wild animals.

Reporting on sex

Indicate if findings apply to only one sex; describe whether sex was considered in study design, methods used for assigning sex. Provide data disaggregated for sex where this information has been collected in the source data as appropriate; provide overall numbers in this Reporting Summary. Please state if this information has not been collected. Report sex-based analyses where performed, justify reasons for lack of sex-based analysis.

Field-collected samples

For laboratory work with field-collected samples, describe all relevant parameters such as housing, maintenance, temperature, photoperiod and end-of-experiment protocol OR state that the study did not involve samples collected from the field.

Ethics oversight

Identify the organization(s) that approved or provided guidance on the study protocol, OR state that no ethical approval or guidance was required and explain why not.

Note that full information on the approval of the study protocol must also be provided in the manuscript.

Clinical data

Policy information about clinical studies

All manuscripts should comply with the ICMJE guidelines for publication of clinical research and a completed CONSORT checklist must be included with all submissions.

Clinical trial registration

Provide the trial registration number from ClinicalTrials.gov or an equivalent agency.

Study protocol	*Note where the full trial protocol can be accessed OR if not available, explain why.*
Data collection	*Describe the settings and locales of data collection, noting the time periods of recruitment and data collection.*
Outcomes	*Describe how you pre-defined primary and secondary outcome measures and how you assessed these measures.*

Dual use research of concern

Policy information about dual use research of concern

Hazards

Could the accidental, deliberate or reckless misuse of agents or technologies generated in the work, or the application of information presented in the manuscript, pose a threat to:

No	Yes	
☒	☐	Public health
☒	☐	National security
☒	☐	Crops and/or livestock
☒	☐	Ecosystems
☒	☐	Any other significant area

Experiments of concern

Does the work involve any of these experiments of concern:

No	Yes	
☒	☐	Demonstrate how to render a vaccine ineffective
☒	☐	Confer resistance to therapeutically useful antibiotics or antiviral agents
☒	☐	Enhance the virulence of a pathogen or render a nonpathogen virulent
☒	☐	Increase transmissibility of a pathogen
☒	☐	Alter the host range of a pathogen
☒	☐	Enable evasion of diagnostic/detection modalities
☒	☐	Enable the weaponization of a biological agent or toxin
☒	☐	Any other potentially harmful combination of experiments and agents

ChIP-seq

Data deposition

☒ Confirm that both raw and final processed data have been deposited in a public database such as GEO.

☒ Confirm that you have deposited or provided access to graph files (e.g. BED files) for the called peaks.

Data access links
May remain private before publication.

https://doi.org/10.5281/ZENODO.6564396

Files in database submission

Rscripts/
Rscripts/allele_support.R
Rscripts/peak_replication.R
peaks/
peaks/h3k4me1_ref_peaks/
peaks/atac_overlaps/
peaks/do.sh
peaks/h3k4me1_hprc_peaks/
peaks/counts.csv
peaks/atac_hprc_peaks/
peaks/subtract_peaks.sh
peaks/atac_ref_peaks/
peaks/h3k27ac_ref_peaks/
peaks/h3k27ac_hprc_peaks/
peaks/h3k4me1_overlaps/
peaks/h3k27ac_overlaps/
peaks/h3k27ac_overlaps/EU21_Flu_2-571406_ChIPmentation-H3K27ac_A00266_0263_3_S24_L003_I_peaks.narrowPeak_ref-only.bed
peaks/h3k27ac_overlaps/AF34_Flu_2-571349_ChIPmentation-H3K27ac_A00266_0263_3_S17_L003_I_peaks.narrowPeak_pers-only.bed

```
peaks/h3k27ac_overlaps/AF20_Flu_2-571333_ChIPmentation-H3K27ac_A00266_0263_3_S1_L003_I_peaks.narrowPeak_ref-only.bed
peaks/h3k27ac_overlaps/AF20_Flu_2-571333_ChIPmentation-H3K27ac_A00266_0263_3_S1_L003_I_peaks.narrowPeak_pers-only.bed
peaks/h3k27ac_overlaps/EU05_Flu_2-571412_ChIPmentation-H3K27ac_A00266_0265_2_S6_L002_I_peaks.narrowPeak_pers-only.bed
peaks/h3k27ac_overlaps/EU13_Flu_2-571348_ChIPmentation-H3K27ac_A00266_0263_3_S16_L003_I_peaks.narrowPeak_ref-only.bed
peaks/h3k27ac_overlaps/AF08_Flu_2-571405_ChIPmentation-H3K27ac_A00266_0263_3_S23_L003_I_peaks.narrowPeak_intersect.bed
peaks/h3k27ac_overlaps/EU19_Flu_2-571338_ChIPmentation-H3K27ac_A00266_0263_3_S6_L003_I_peaks.narrowPeak_pers-only.bed
peaks/h3k27ac_overlaps/AF04_Flu_2-642070_ChIPmentation-H3K27ac_A00266_0265_2_S14_L002_I_peaks.narrowPeak_ref-only.bed
peaks/h3k27ac_overlaps/EU27_Flu_2-642077_ChIPmentation-H3K27ac_A00266_0265_2_S21_L002_I_peaks.narrowPeak_intersect.bed
peaks/h3k27ac_overlaps/EU39_Flu_2-571401_ChIPmentation-H3K27ac_A00266_0263_3_S19_L003_I_peaks.narrowPeak_ref-only.bed
peaks/h3k27ac_overlaps/AF30_Flu_2-669476_ChIPmentation-H3K27ac_A00266_0289_1_S12_L001_I_peaks.narrowPeak_intersect.bed
peaks/h3k27ac_overlaps/EU07_Flu_2-642067_ChIPmentation-H3K27ac_A00266_0265_2_S11_L002_I_peaks.narrowPeak_ref-only.bed
peaks/h3k27ac_overlaps/EU15_Flu_2-571342_ChIPmentation-H3K27ac_A00266_0263_3_S10_L003_I_peaks.narrowPeak_intersect.bed
peaks/h3k27ac_overlaps/EU13_Flu_2-571348_ChIPmentation-H3K27ac_A00266_0263_3_S16_L003_I_peaks.narrowPeak_intersect.bed
peaks/h3k27ac_overlaps/AF12_Flu_2-571340_ChIPmentation-H3K27ac_A00266_0263_3_S8_L003_I_peaks.narrowPeak_pers-only.bed
peaks/h3k27ac_overlaps/AF36_Flu_2-571346_ChIPmentation-H3K27ac_A00266_0263_3_S14_L003_I_peaks.narrowPeak_intersect.bed
peaks/h3k27ac_overlaps/AF04_Flu_2-642070_ChIPmentation-H3K27ac_A00266_0265_2_S14_L002_I_peaks.narrowPeak_pers-only.bed
peaks/h3k27ac_overlaps/AF22_Flu_2-669473_ChIPmentation-H3K27ac_A00266_0289_1_S9_L001_I_peaks.narrowPeak_pers-only.bed
peaks/h3k27ac_overlaps/EU47_Flu_2-669480_ChIPmentation-H3K27ac_A00266_0289_1_S16_L001_I_peaks.narrowPeak_pers-only.bed
peaks/h3k27ac_overlaps/AF34_Flu_2-571349_ChIPmentation-H3K27ac_A00266_0263_3_S17_L003_I_peaks.narrowPeak_ref-only.bed
peaks/h3k27ac_overlaps/EU03_Flu_2-571411_ChIPmentation-H3K27ac_A00266_0265_2_S5_L002_I_peaks.narrowPeak_ref-only.bed
peaks/h3k27ac_overlaps/EU21_Flu_2-571406_ChIPmentation-H3K27ac_A00266_0263_3_S24_L003_I_peaks.narrowPeak_intersect.bed
peaks/h3k27ac_overlaps/EU21_Flu_2-571406_ChIPmentation-H3K27ac_A00266_0263_3_S24_L003_I_peaks.narrowPeak_pers-only.bed
peaks/h3k27ac_overlaps/AF22_Flu_2-669473_ChIPmentation-H3K27ac_A00266_0289_1_S9_L001_I_peaks.narrowPeak_intersect.bed
peaks/h3k27ac_overlaps/EU47_Flu_2-669480_ChIPmentation-H3K27ac_A00266_0289_1_S16_L001_I_peaks.narrowPeak_intersect.bed
peaks/h3k27ac_overlaps/AF04_Flu_2-642070_ChIPmentation-H3K27ac_A00266_0265_2_S14_L002_I_peaks.narrowPeak_intersect.bed
peaks/h3k27ac_overlaps/AF36_Flu_2-571346_ChIPmentation-H3K27ac_A00266_0263_3_S14_L003_I_peaks.narrowPeak_pers-only.bed
peaks/h3k27ac_overlaps/AF12_Flu_2-571340_ChIPmentation-H3K27ac_A00266_0263_3_S8_L003_I_peaks.narrowPeak_intersect.bed
peaks/h3k27ac_overlaps/AF18_Flu_2-642068_ChIPmentation-H3K27ac_A00266_0265_2_S12_L002_I_peaks.narrowPeak_ref-only.bed
peaks/h3k27ac_overlaps/EU19_Flu_2-571338_ChIPmentation-H3K27ac_A00266_0263_3_S6_L003_I_peaks.narrowPeak_ref-only.bed
peaks/h3k27ac_overlaps/EU13_Flu_2-571348_ChIPmentation-H3K27ac_A00266_0263_3_S16_L003_I_peaks.narrowPeak_pers-only.bed
peaks/h3k27ac_overlaps/EU15_Flu_2-571342_ChIPmentation-H3K27ac_A00266_0263_3_S10_L003_I_peaks.narrowPeak_pers-only.bed
peaks/h3k27ac_overlaps/AF30_Flu_2-669476_ChIPmentation-H3K27ac_A00266_0289_1_S12_L001_I_peaks.narrowPeak_pers-only.bed
peaks/h3k27ac_overlaps/EU27_Flu_2-642077_ChIPmentation-H3K27ac_A00266_0265_2_S21_L002_I_peaks.narrowPeak_pers-only.bed
peaks/h3k27ac_overlaps/EU19_Flu_2-571338_ChIPmentation-H3K27ac_A00266_0263_3_S6_L003_I_peaks.narrowPeak_intersect.bed
peaks/h3k27ac_overlaps/AF08_Flu_2-571405_ChIPmentation-H3K27ac_A00266_0263_3_S23_L003_I_peaks.narrowPeak_pers-only.bed
peaks/h3k27ac_overlaps/EU33_Flu_2-669466_ChIPmentation-H3K27ac_A00266_0289_1_S2_L001_I_peaks.narrowPeak_ref-only.bed
peaks/h3k27ac_overlaps/AF30_Flu_2-669476_ChIPmentation-H3K27ac_A00266_0289_1_S12_L001_I_peaks.narrowPeak_ref-only.bed
peaks/h3k27ac_overlaps/EU05_Flu_2-571412_ChIPmentation-H3K27ac_A00266_0265_2_S6_L002_I_peaks.narrowPeak_intersect.bed
```

peaks/h3k27ac_overlaps/AF20_Flu_2-571333_ChIPmentation-H3K27ac_A00266_0263_3_S1_L003_I_peaks.narrowPeak_intersect.bed
peaks/h3k27ac_overlaps/AF34_Flu_2-571349_ChIPmentation-H3K27ac_A00266_0263_3_S17_L003_I_peaks.narrowPeak_intersect.bed
peaks/h3k27ac_overlaps/EU39_Flu_2-571401_ChIPmentation-H3K27ac_A00266_0263_3_S19_L003_I_peaks.narrowPeak_intersect.bed
peaks/h3k27ac_overlaps/EU03_Flu_2-571411_ChIPmentation-H3K27ac_A00266_0265_2_S5_L002_I_peaks.narrowPeak_pers-only.bed
peaks/h3k27ac_overlaps/AF24_Flu_2-642069_ChIPmentation-H3K27ac_A00266_0265_2_S13_L002_I_peaks.narrowPeak_intersect.bed
peaks/h3k27ac_overlaps/EU15_Flu_2-571342_ChIPmentation-H3K27ac_A00266_0263_3_S10_L003_I_peaks.narrowPeak_ref-only.bed
peaks/h3k27ac_overlaps/AF12_Flu_2-571340_ChIPmentation-H3K27ac_A00266_0263_3_S8_L003_I_peaks.narrowPeak_ref-only.bed
peaks/h3k27ac_overlaps/AF26_Flu_2-571344_ChIPmentation-H3K27ac_A00266_0263_3_S12_L003_I_peaks.narrowPeak_intersect.bed
peaks/h3k27ac_overlaps/EU33_Flu_2-669466_ChIPmentation-H3K27ac_A00266_0289_1_S2_L001_I_peaks.narrowPeak_pers-only.bed
peaks/h3k27ac_overlaps/EU07_Flu_2-642067_ChIPmentation-H3K27ac_A00266_0265_2_S11_L002_I_peaks.narrowPeak_pers-only.bed
peaks/h3k27ac_overlaps/EU25_Flu_2-571343_ChIPmentation-H3K27ac_A00266_0263_3_S11_L003_I_peaks.narrowPeak_pers-only.bed
peaks/h3k27ac_overlaps/EU41_Flu_2-669478_ChIPmentation-H3K27ac_A00266_0289_1_S14_L001_I_peaks.narrowPeak_intersect.bed
peaks/h3k27ac_overlaps/AF38_Flu_2-669483_ChIPmentation-H3K27ac_A00266_0289_1_S19_L001_I_peaks.narrowPeak_intersect.bed
peaks/h3k27ac_overlaps/AF18_Flu_2-642068_ChIPmentation-H3K27ac_A00266_0265_2_S12_L002_I_peaks.narrowPeak_intersect.bed
peaks/h3k27ac_overlaps/AF08_Flu_2-571405_ChIPmentation-H3K27ac_A00266_0263_3_S23_L003_I_peaks.narrowPeak_ref-only.bed
peaks/h3k27ac_overlaps/EU09_Flu_2-571335_ChIPmentation-H3K27ac_A00266_0263_3_S3_L003_I_peaks.narrowPeak_intersect.bed
peaks/h3k27ac_overlaps/EU29_Flu_2-669467_ChIPmentation-H3K27ac_A00266_0289_1_S3_L001_I_peaks.narrowPeak_ref-only.bed
peaks/h3k27ac_overlaps/AF06_Flu_2-642071_ChIPmentation-H3K27ac_A00266_0265_2_S15_L002_I_peaks.narrowPeak_intersect.bed
peaks/h3k27ac_overlaps/EU29_Flu_2-669467_ChIPmentation-H3K27ac_A00266_0289_1_S3_L001_I_peaks.narrowPeak_intersect.bed
peaks/h3k27ac_overlaps/AF16_Flu_2-571407_ChIPmentation-H3K27ac_A00266_0265_2_S1_L002_I_peaks.narrowPeak_intersect.bed
peaks/h3k27ac_overlaps/EU41_Flu_2-669478_ChIPmentation-H3K27ac_A00266_0289_1_S14_L001_I_peaks.narrowPeak_ref-only.bed
peaks/h3k27ac_overlaps/AF28_Flu_2-571345_ChIPmentation-H3K27ac_A00266_0263_3_S13_L003_I_peaks.narrowPeak_intersect.bed
peaks/h3k27ac_overlaps/AF28_Flu_2-571345_ChIPmentation-H3K27ac_A00266_0263_3_S13_L003_I_peaks.narrowPeak_pers-only.bed
peaks/h3k27ac_overlaps/EU09_Flu_2-571335_ChIPmentation-H3K27ac_A00266_0263_3_S3_L003_I_peaks.narrowPeak_ref-only.bed
peaks/h3k27ac_overlaps/AF16_Flu_2-571407_ChIPmentation-H3K27ac_A00266_0265_2_S1_L002_I_peaks.narrowPeak_pers-only.bed
peaks/h3k27ac_overlaps/EU27_Flu_2-642077_ChIPmentation-H3K27ac_A00266_0265_2_S21_L002_I_peaks.narrowPeak_ref-only.bed
peaks/h3k27ac_overlaps/EU25_Flu_2-571343_ChIPmentation-H3K27ac_A00266_0263_3_S11_L003_I_peaks.narrowPeak_ref-only.bed
peaks/h3k27ac_overlaps/AF22_Flu_2-669473_ChIPmentation-H3K27ac_A00266_0289_1_S9_L001_I_peaks.narrowPeak_ref-only.bed
peaks/h3k27ac_overlaps/AF24_Flu_2-642069_ChIPmentation-H3K27ac_A00266_0265_2_S13_L002_I_peaks.narrowPeak_ref-only.bed
peaks/h3k27ac_overlaps/EU05_Flu_2-571412_ChIPmentation-H3K27ac_A00266_0265_2_S6_L002_I_peaks.narrowPeak_ref-only.bed
peaks/h3k27ac_overlaps/AF36_Flu_2-571346_ChIPmentation-H3K27ac_A00266_0263_3_S14_L003_I_peaks.narrowPeak_ref-only.bed
peaks/h3k27ac_overlaps/EU29_Flu_2-669467_ChIPmentation-H3K27ac_A00266_0289_1_S3_L001_I_peaks.narrowPeak_pers-only.bed
peaks/h3k27ac_overlaps/AF06_Flu_2-642071_ChIPmentation-H3K27ac_A00266_0265_2_S15_L002_I_peaks.narrowPeak_ref-only.bed
peaks/h3k27ac_overlaps/EU09_Flu_2-571335_ChIPmentation-H3K27ac_A00266_0263_3_S3_L003_I_peaks.narrowPeak_pers-only.bed
peaks/h3k27ac_overlaps/AF06_Flu_2-642071_ChIPmentation-H3K27ac_A00266_0265_2_S15_L002_I_peaks.narrowPeak_pers-only.bed
peaks/h3k27ac_overlaps/EU47_Flu_2-669480_ChIPmentation-H3K27ac_A00266_0289_1_S16_L001_I_peaks.narrowPeak_ref-only.bed
peaks/h3k27ac_overlaps/AF38_Flu_2-669483_ChIPmentation-H3K27ac_A00266_0289_1_S19_L001_I_peaks.narrowPeak_ref-only.bed
peaks/h3k27ac_overlaps/AF18_Flu_2-642068_ChIPmentation-H3K27ac_A00266_0265_2_S12_L002_I_peaks.narrowPeak_pers-only.bed

peaks/h3k27ac_overlaps/AF26_Flu_2-571344_ChIPmentation-H3K27ac_A00266_0263_3_S12_L003_I_peaks.narrowPeak_ref-only.bed
peaks/h3k27ac_overlaps/AF38_Flu_2-669483_ChIPmentation-H3K27ac_A00266_0289_1_S19_L001_I_peaks.narrowPeak_pers-only.bed
peaks/h3k27ac_overlaps/EU25_Flu_2-571343_ChIPmentation-H3K27ac_A00266_0263_3_S11_L003_I_peaks.narrowPeak_intersect.bed
peaks/h3k27ac_overlaps/EU41_Flu_2-669478_ChIPmentation-H3K27ac_A00266_0289_1_S14_L001_I_peaks.narrowPeak_pers-only.bed
peaks/h3k27ac_overlaps/EU33_Flu_2-669466_ChIPmentation-H3K27ac_A00266_0289_1_S2_L001_I_peaks.narrowPeak_intersect.bed
peaks/h3k27ac_overlaps/EU07_Flu_2-642067_ChIPmentation-H3K27ac_A00266_0265_2_S11_L002_I_peaks.narrowPeak_intersect.bed
peaks/h3k27ac_overlaps/AF16_Flu_2-571407_ChIPmentation-H3K27ac_A00266_0265_2_S1_L002_I_peaks.narrowPeak_ref-only.bed
peaks/h3k27ac_overlaps/AF28_Flu_2-571345_ChIPmentation-H3K27ac_A00266_0263_3_S13_L003_I_peaks.narrowPeak_ref-only.bed
peaks/h3k27ac_overlaps/AF26_Flu_2-571344_ChIPmentation-H3K27ac_A00266_0263_3_S12_L003_I_peaks.narrowPeak_pers-only.bed
peaks/h3k27ac_overlaps/EU03_Flu_2-571411_ChIPmentation-H3K27ac_A00266_0265_2_S5_L002_I_peaks.narrowPeak_intersect.bed
peaks/h3k27ac_overlaps/AF24_Flu_2-642069_ChIPmentation-H3K27ac_A00266_0265_2_S13_L002_I_peaks.narrowPeak_pers-only.bed
peaks/h3k27ac_overlaps/EU39_Flu_2-571401_ChIPmentation-H3K27ac_A00266_0263_3_S19_L003_I_peaks.narrowPeak_pers-only.bed
peaks/h3k4me1_overlaps/AF38_Flu_2-674837_ChIPmentation-H3K4me1_A00266_0301_2_S14_L002_I_peaks.narrowPeak_ref-only.bed
peaks/h3k4me1_overlaps/AF36_Flu_2-669593_ChIPmentation-H3K4me1_A00266_0289_2_S10_L002_I_peaks.narrowPeak_intersect.bed
peaks/h3k4me1_overlaps/AF24_Flu_2-674804_ChIPmentation-H3K4me1_A00266_0301_1_S5_L001_I_peaks.narrowPeak_intersect.bed
peaks/h3k4me1_overlaps/AF20_Flu_2-669580_ChIPmentation-H3K4me1_A00266_0289_1_S21_L001_I_peaks.narrowPeak_ref-only.bed
peaks/h3k4me1_overlaps/AF18_Flu_2-674803_ChIPmentation-H3K4me1_A00266_0301_1_S4_L001_I_peaks.narrowPeak_ref-only.bed
peaks/h3k4me1_overlaps/AF30_Flu_2-674830_ChIPmentation-H3K4me1_A00266_0301_2_S7_L002_I_peaks.narrowPeak_pers-only.bed
peaks/h3k4me1_overlaps/AF06_Flu_2-674806_ChIPmentation-H3K4me1_A00266_0301_1_S7_L001_I_peaks.narrowPeak_ref-only.bed
peaks/h3k4me1_overlaps/AF16_Flu_2-669654_ChIPmentation-H3K4me1_A00266_0289_2_S21_L002_I_peaks.narrowPeak_pers-only.bed
peaks/h3k4me1_overlaps/EU13_Flu_2-669595_ChIPmentation-H3K4me1A00266_0289_2_S12_L002_I_peaks.narrowPeak_pers-only.bed
peaks/h3k4me1_overlaps/EU29_Flu_2-674821_ChIPmentation-H3K4me1A00266_0301_1_S22_L001_I_peaks.narrowPeak_intersect.bed
peaks/h3k4me1_overlaps/EU27_Flu_2-674815_ChIPmentation-H3K4me1A00266_0301_1_S16_L001_I_peaks.narrowPeak_ref-only.bed
peaks/h3k4me1_overlaps/EU09_Flu_2-669582_ChIPmentation-H3K4me1A00266_0289_1_S23_L001_I_peaks.narrowPeak_intersect.bed
peaks/h3k4me1_overlaps/AF28_Flu_2-669592_ChIPmentation-H3K4me1_A00266_0289_2_S9_L002_I_peaks.narrowPeak_pers-only.bed
peaks/h3k4me1_overlaps/AF12_Flu_2-669587_ChIPmentation-H3K4me1_A00266_0289_2_S4_L002_I_peaks.narrowPeak_intersect.bed
peaks/h3k4me1_overlaps/EU25_Flu_2-669590_ChIPmentation-H3K4me1A00266_0289_2_S7_L002_I_peaks.narrowPeak_pers-only.bed
peaks/h3k4me1_overlaps/AF18_Flu_2-674803_ChIPmentation-H3K4me1_A00266_0301_1_S4_L001_I_peaks.narrowPeak_pers-only.bed
peaks/h3k4me1_overlaps/AF22_Flu_2-674827_ChIPmentation-H3K4me1_A00266_0301_2_S4_L002_I_peaks.narrowPeak_ref-only.bed
peaks/h3k4me1_overlaps/EU13_Flu_2-669595_ChIPmentation-H3K4me1A00266_0289_2_S12_L002_I_peaks.narrowPeak_ref-only.bed
peaks/h3k4me1_overlaps/EU33_Flu_2-674820_ChIPmentation-H3K4me1A00266_0301_1_S21_L001_I_peaks.narrowPeak_pers-only.bed
peaks/h3k4me1_overlaps/AF26_Flu_2-669591_ChIPmentation-H3K4me1_A00266_0289_2_S8_L002_I_peaks.narrowPeak_ref-only.bed
peaks/h3k4me1_overlaps/EU27_Flu_2-674815_ChIPmentation-H3K4me1A00266_0301_1_S16_L001_I_peaks.narrowPeak_intersect.bed
peaks/h3k4me1_overlaps/EU07_Flu_2-674802_ChIPmentation-H3K4me1A00266_0301_1_S3_L001_I_peaks.narrowPeak_pers-only.bed
peaks/h3k4me1_overlaps/AF34_Flu_2-669596_ChIPmentation-H3K4me1_A00266_0289_2_S13_L002_I_peaks.narrowPeak_intersect.bed
peaks/h3k4me1_overlaps/AF04_Flu_2-674805_ChIPmentation-H3K4me1_A00266_0301_1_S6_L001_I_peaks.narrowPeak_ref-only.bed
peaks/h3k4me1_overlaps/AF38_Flu_2-674837_ChIPmentation-H3K4me1_A00266_0301_2_S14_L002_I_peaks.narrowPeak_intersect.bed
peaks/h3k4me1_overlaps/EU05_Flu_2-674801_ChIPmentation-H3K4me1A00266_0301_1_S2_L001_I_peaks.narrowPeak_pers-only.bed

peaks/h3k4me1_overlaps/EU41_Flu_2-674832_ChIPmentation-
H3K4me1A00266_0301_2_S9_L002_I_peaks.narrowPeak_pers-only.bed
peaks/h3k4me1_overlaps/AF36_Flu_2-669593_ChIPmentation-
H3K4me1_A00266_0289_2_S10_L002_I_peaks.narrowPeak_ref-only.bed
peaks/h3k4me1_overlaps/EU39_Flu_2-669598_ChIPmentation-
H3K4me1A00266_0289_2_S15_L002_I_peaks.narrowPeak_intersect.bed
peaks/h3k4me1_overlaps/EU39_Flu_2-669598_ChIPmentation-
H3K4me1A00266_0289_2_S15_L002_I_peaks.narrowPeak_pers-only.bed
peaks/h3k4me1_overlaps/EU03_Flu_2-674750_ChIPmentation-
H3K4me1A00266_0301_1_S1_L001_I_peaks.narrowPeak_ref-only.bed
peaks/h3k4me1_overlaps/EU29_Flu_2-674821_ChIPmentation-
H3K4me1A00266_0301_1_S22_L001_I_peaks.narrowPeak_ref-only.bed
peaks/h3k4me1_overlaps/EU41_Flu_2-674832_ChIPmentation-
H3K4me1A00266_0301_2_S9_L002_I_peaks.narrowPeak_intersect.bed
peaks/h3k4me1_overlaps/EU05_Flu_2-674801_ChIPmentation-
H3K4me1A00266_0301_1_S2_L001_I_peaks.narrowPeak_intersect.bed
peaks/h3k4me1_overlaps/AF38_Flu_2-674837_ChIPmentation-
H3K4me1_A00266_0301_2_S14_L002_I_peaks.narrowPeak_pers-only.bed
peaks/h3k4me1_overlaps/AF08_Flu_2-669652_ChIPmentation-
H3K4me1_A00266_0289_2_S19_L002_I_peaks.narrowPeak_ref-only.bed
peaks/h3k4me1_overlaps/AF34_Flu_2-669596_ChIPmentation-
H3K4me1_A00266_0289_2_S13_L002_I_peaks.narrowPeak_pers-only.bed
peaks/h3k4me1_overlaps/EU15_Flu_2-669589_ChIPmentation-
H3K4me1A00266_0289_2_S6_L002_I_peaks.narrowPeak_ref-only.bed
peaks/h3k4me1_overlaps/EU27_Flu_2-674815_ChIPmentation-
H3K4me1A00266_0301_1_S16_L001_I_peaks.narrowPeak_pers-only.bed
peaks/h3k4me1_overlaps/EU07_Flu_2-674802_ChIPmentation-
H3K4me1A00266_0301_1_S3_L001_I_peaks.narrowPeak_intersect.bed
peaks/h3k4me1_overlaps/EU47_Flu_2-674834_ChIPmentation-
H3K4me1A00266_0301_2_S11_L002_I_peaks.narrowPeak_ref-only.bed
peaks/h3k4me1_overlaps/AF34_Flu_2-669596_ChIPmentation-
H3K4me1_A00266_0289_2_S13_L002_I_peaks.narrowPeak_ref-only.bed
peaks/h3k4me1_overlaps/EU33_Flu_2-674820_ChIPmentation-
H3K4me1A00266_0301_1_S21_L001_I_peaks.narrowPeak_intersect.bed
peaks/h3k4me1_overlaps/AF18_Flu_2-674803_ChIPmentation-
H3K4me1_A00266_0301_1_S4_L001_I_peaks.narrowPeak_intersect.bed
peaks/h3k4me1_overlaps/EU25_Flu_2-669590_ChIPmentation-
H3K4me1A00266_0289_2_S7_L002_I_peaks.narrowPeak_intersect.bed
peaks/h3k4me1_overlaps/AF12_Flu_2-669587_ChIPmentation-
H3K4me1_A00266_0289_2_S4_L002_I_peaks.narrowPeak_pers-only.bed
peaks/h3k4me1_overlaps/AF28_Flu_2-669592_ChIPmentation-
H3K4me1_A00266_0289_2_S9_L002_I_peaks.narrowPeak_intersect.bed
peaks/h3k4me1_overlaps/EU33_Flu_2-674820_ChIPmentation-
H3K4me1A00266_0301_1_S21_L001_I_peaks.narrowPeak_ref-only.bed
peaks/h3k4me1_overlaps/EU09_Flu_2-669582_ChIPmentation-
H3K4me1A00266_0289_1_S23_L001_I_peaks.narrowPeak_pers-only.bed
peaks/h3k4me1_overlaps/AF16_Flu_2-669654_ChIPmentation-
H3K4me1_A00266_0289_2_S21_L002_I_peaks.narrowPeak_intersect.bed
peaks/h3k4me1_overlaps/EU13_Flu_2-669595_ChIPmentation-
H3K4me1A00266_0289_2_S12_L002_I_peaks.narrowPeak_intersect.bed
peaks/h3k4me1_overlaps/EU29_Flu_2-674821_ChIPmentation-
H3K4me1A00266_0301_1_S22_L001_I_peaks.narrowPeak_pers-only.bed
peaks/h3k4me1_overlaps/EU19_Flu_2-669585_ChIPmentation-
H3K4me1A00266_0289_2_S2_L002_I_peaks.narrowPeak_ref-only.bed
peaks/h3k4me1_overlaps/AF30_Flu_2-674830_ChIPmentation-
H3K4me1_A00266_0301_2_S7_L002_I_peaks.narrowPeak_intersect.bed
peaks/h3k4me1_overlaps/AF24_Flu_2-674804_ChIPmentation-
H3K4me1_A00266_0301_1_S5_L001_I_peaks.narrowPeak_pers-only.bed
peaks/h3k4me1_overlaps/AF36_Flu_2-669593_ChIPmentation-
H3K4me1_A00266_0289_2_S10_L002_I_peaks.narrowPeak_pers-only.bed
peaks/h3k4me1_overlaps/AF08_Flu_2-669652_ChIPmentation-
H3K4me1_A00266_0289_2_S19_L002_I_peaks.narrowPeak_intersect.bed
peaks/h3k4me1_overlaps/AF26_Flu_2-669591_ChIPmentation-
H3K4me1_A00266_0289_2_S8_L002_I_peaks.narrowPeak_pers-only.bed
peaks/h3k4me1_overlaps/AF22_Flu_2-674827_ChIPmentation-
H3K4me1_A00266_0301_2_S4_L002_I_peaks.narrowPeak_pers-only.bed
peaks/h3k4me1_overlaps/EU15_Flu_2-669589_ChIPmentation-
H3K4me1A00266_0289_2_S6_L002_I_peaks.narrowPeak_pers-only.bed
peaks/h3k4me1_overlaps/EU19_Flu_2-669585_ChIPmentation-
H3K4me1A00266_0289_2_S2_L002_I_peaks.narrowPeak_intersect.bed
peaks/h3k4me1_overlaps/EU39_Flu_2-669598_ChIPmentation-
H3K4me1A00266_0289_2_S15_L002_I_peaks.narrowPeak_ref-only.bed
peaks/h3k4me1_overlaps/EU21_Flu_2-669653_ChIPmentation-
H3K4me1A00266_0289_2_S20_L002_I_peaks.narrowPeak_intersect.bed
peaks/h3k4me1_overlaps/AF12_Flu_2-669587_ChIPmentation-
H3K4me1_A00266_0289_2_S4_L002_I_peaks.narrowPeak_ref-only.bed

peaks/h3k4me1_overlaps/AF06_Flu_2-674806_ChIPmentation-H3K4me1_A00266_0301_1_S7_L001_I_peaks.narrowPeak_intersect.bed
peaks/h3k4me1_overlaps/AF20_Flu_2-669580_ChIPmentation-H3K4me1_A00266_0289_1_S21_L001_I_peaks.narrowPeak_pers-only.bed
peaks/h3k4me1_overlaps/AF04_Flu_2-674805_ChIPmentation-H3K4me1_A00266_0301_1_S6_L001_I_peaks.narrowPeak_pers-only.bed
peaks/h3k4me1_overlaps/EU05_Flu_2-674801_ChIPmentation-H3K4me1A00266_0301_1_S2_L001_I_peaks.narrowPeak_ref-only.bed
peaks/h3k4me1_overlaps/AF16_Flu_2-669654_ChIPmentation-H3K4me1_A00266_0289_2_S21_L002_I_peaks.narrowPeak_ref-only.bed
peaks/h3k4me1_overlaps/EU03_Flu_2-674750_ChIPmentation-H3K4me1A00266_0301_1_S1_L001_I_peaks.narrowPeak_intersect.bed
peaks/h3k4me1_overlaps/EU47_Flu_2-674834_ChIPmentation-H3K4me1A00266_0301_2_S11_L002_I_peaks.narrowPeak_pers-only.bed
peaks/h3k4me1_overlaps/EU41_Flu_2-674832_ChIPmentation-H3K4me1A00266_0301_2_S9_L002_I_peaks.narrowPeak_ref-only.bed
peaks/h3k4me1_overlaps/AF30_Flu_2-674830_ChIPmentation-H3K4me1_A00266_0301_2_S7_L002_I_peaks.narrowPeak_ref-only.bed
peaks/h3k4me1_overlaps/EU47_Flu_2-674834_ChIPmentation-H3K4me1A00266_0301_2_S11_L002_I_peaks.narrowPeak_intersect.bed
peaks/h3k4me1_overlaps/EU25_Flu_2-669590_ChIPmentation-H3K4me1A00266_0289_2_S7_L002_I_peaks.narrowPeak_ref-only.bed
peaks/h3k4me1_overlaps/EU03_Flu_2-674750_ChIPmentation-H3K4me1A00266_0301_1_S1_L001_I_peaks.narrowPeak_pers-only.bed
peaks/h3k4me1_overlaps/AF24_Flu_2-674804_ChIPmentation-H3K4me1_A00266_0301_1_S5_L001_I_peaks.narrowPeak_ref-only.bed
peaks/h3k4me1_overlaps/AF04_Flu_2-674805_ChIPmentation-H3K4me1_A00266_0301_1_S6_L001_I_peaks.narrowPeak_intersect.bed
peaks/h3k4me1_overlaps/AF20_Flu_2-669580_ChIPmentation-H3K4me1_A00266_0289_1_S21_L001_I_peaks.narrowPeak_intersect.bed
peaks/h3k4me1_overlaps/EU07_Flu_2-674802_ChIPmentation-H3K4me1A00266_0301_1_S3_L001_I_peaks.narrowPeak_ref-only.bed
peaks/h3k4me1_overlaps/AF06_Flu_2-674806_ChIPmentation-H3K4me1_A00266_0301_1_S7_L001_I_peaks.narrowPeak_pers-only.bed
peaks/h3k4me1_overlaps/EU21_Flu_2-669653_ChIPmentation-H3K4me1A00266_0289_2_S20_L002_I_peaks.narrowPeak_pers-only.bed
peaks/h3k4me1_overlaps/EU19_Flu_2-669585_ChIPmentation-H3K4me1A00266_0289_2_S2_L002_I_peaks.narrowPeak_pers-only.bed
peaks/h3k4me1_overlaps/EU21_Flu_2-669653_ChIPmentation-H3K4me1A00266_0289_2_S20_L002_I_peaks.narrowPeak_ref-only.bed
peaks/h3k4me1_overlaps/EU15_Flu_2-669589_ChIPmentation-H3K4me1A00266_0289_2_S6_L002_I_peaks.narrowPeak_intersect.bed
peaks/h3k4me1_overlaps/AF28_Flu_2-669592_ChIPmentation-H3K4me1_A00266_0289_2_S9_L002_I_peaks.narrowPeak_ref-only.bed
peaks/h3k4me1_overlaps/AF22_Flu_2-674827_ChIPmentation-H3K4me1_A00266_0301_2_S4_L002_I_peaks.narrowPeak_intersect.bed
peaks/h3k4me1_overlaps/EU09_Flu_2-669582_ChIPmentation-H3K4me1A00266_0289_1_S23_L001_I_peaks.narrowPeak_ref-only.bed
peaks/h3k4me1_overlaps/AF26_Flu_2-669591_ChIPmentation-H3K4me1_A00266_0289_2_S8_L002_I_peaks.narrowPeak_intersect.bed
peaks/h3k4me1_overlaps/AF08_Flu_2-669652_ChIPmentation-H3K4me1_A00266_0289_2_S19_L002_I_peaks.narrowPeak_pers-only.bed
peaks/h3k27ac_hprc_peaks/AF24_Flu_2-642069_ChIPmentation-H3K27ac_A00266_0265_2_S13_L002_I_peaks.narrowPeak
peaks/h3k27ac_hprc_peaks/AF18_Flu_2-642068_ChIPmentation-H3K27ac_A00266_0265_2_S12_L002_I_peaks.narrowPeak
peaks/h3k27ac_hprc_peaks/EU05_Flu_2-571412_ChIPmentation-H3K27ac_A00266_0265_2_S6_L002_I_peaks.narrowPeak
peaks/h3k27ac_hprc_peaks/AF22_Flu_2-669473_ChIPmentation-H3K27ac_A00266_0289_1_S9_L001_I_peaks.narrowPeak
peaks/h3k27ac_hprc_peaks/EU47_Flu_2-669480_ChIPmentation-H3K27ac_A00266_0289_1_S16_L001_I_peaks.narrowPeak
peaks/h3k27ac_hprc_peaks/EU19_Flu_2-571338_ChIPmentation-H3K27ac_A00266_0263_3_S6_L003_I_peaks.narrowPeak
peaks/h3k27ac_hprc_peaks/AF30_Flu_2-669476_ChIPmentation-H3K27ac_A00266_0289_1_S12_L001_I_peaks.narrowPeak
peaks/h3k27ac_hprc_peaks/EU09_Flu_2-571335_ChIPmentation-H3K27ac_A00266_0263_3_S3_L003_I_peaks.narrowPeak
peaks/h3k27ac_hprc_peaks/AF26_Flu_2-571344_ChIPmentation-H3K27ac_A00266_0263_3_S12_L003_I_peaks.narrowPeak
peaks/h3k27ac_hprc_peaks/EU33_Flu_2-669466_ChIPmentation-H3K27ac_A00266_0289_1_S2_L001_I_peaks.narrowPeak
peaks/h3k27ac_hprc_peaks/EU25_Flu_2-571343_ChIPmentation-H3K27ac_A00266_0263_3_S11_L003_I_peaks.narrowPeak
peaks/h3k27ac_hprc_peaks/AF38_Flu_2-669483_ChIPmentation-H3K27ac_A00266_0289_1_S19_L001_I_peaks.narrowPeak
peaks/h3k27ac_hprc_peaks/EU21_Flu_2-571406_ChIPmentation-H3K27ac_A00266_0263_3_S24_L003_I_peaks.narrowPeak
peaks/h3k27ac_hprc_peaks/AF06_Flu_2-642071_ChIPmentation-H3K27ac_A00266_0265_2_S15_L002_I_peaks.narrowPeak
peaks/h3k27ac_hprc_peaks/EU15_Flu_2-571342_ChIPmentation-H3K27ac_A00266_0263_3_S10_L003_I_peaks.narrowPeak
peaks/h3k27ac_hprc_peaks/AF16_Flu_2-571407_ChIPmentation-H3K27ac_A00266_0265_2_S1_L002_I_peaks.narrowPeak
peaks/h3k27ac_hprc_peaks/AF08_Flu_2-571405_ChIPmentation-H3K27ac_A00266_0263_3_S23_L003_I_peaks.narrowPeak
peaks/h3k27ac_hprc_peaks/EU13_Flu_2-571348_ChIPmentation-H3K27ac_A00266_0263_3_S16_L003_I_peaks.narrowPeak
peaks/h3k27ac_hprc_peaks/AF36_Flu_2-571346_ChIPmentation-H3K27ac_A00266_0263_3_S14_L003_I_peaks.narrowPeak
peaks/h3k27ac_hprc_peaks/AF34_Flu_2-571349_ChIPmentation-H3K27ac_A00266_0263_3_S17_L003_I_peaks.narrowPeak
peaks/h3k27ac_hprc_peaks/AF12_Flu_2-571340_ChIPmentation-H3K27ac_A00266_0263_3_S8_L003_I_peaks.narrowPeak
peaks/h3k27ac_hprc_peaks/EU03_Flu_2-571411_ChIPmentation-H3K27ac_A00266_0265_2_S5_L002_I_peaks.narrowPeak
peaks/h3k27ac_hprc_peaks/EU41_Flu_2-669478_ChIPmentation-H3K27ac_A00266_0289_1_S14_L001_I_peaks.narrowPeak
peaks/h3k27ac_hprc_peaks/AF20_Flu_2-571333_ChIPmentation-H3K27ac_A00266_0263_3_S1_L003_I_peaks.narrowPeak

peaks/h3k27ac_hprc_peaks/EU29_Flu_2-669467_ChIPmentation-H3K27ac_A00266_0289_1_S3_L001_I_peaks.narrowPeak
peaks/h3k27ac_hprc_peaks/AF04_Flu_2-642070_ChIPmentation-H3K27ac_A00266_0265_2_S14_L002_I_peaks.narrowPeak
peaks/h3k27ac_hprc_peaks/EU39_Flu_2-571401_ChIPmentation-H3K27ac_A00266_0263_3_S19_L003_I_peaks.narrowPeak
peaks/h3k27ac_hprc_peaks/AF28_Flu_2-571345_ChIPmentation-H3K27ac_A00266_0263_3_S13_L003_I_peaks.narrowPeak
peaks/h3k27ac_hprc_peaks/EU07_Flu_2-642067_ChIPmentation-H3K27ac_A00266_0265_2_S11_L002_I_peaks.narrowPeak
peaks/h3k27ac_hprc_peaks/EU27_Flu_2-642077_ChIPmentation-H3K27ac_A00266_0265_2_S21_L002_I_peaks.narrowPeak
peaks/h3k27ac_ref_peaks/AF24_Flu_2-642069_ChIPmentation-H3K27ac_A00266_0265_2_S13_L002_I_peaks.narrowPeak
peaks/h3k27ac_ref_peaks/AF18_Flu_2-642068_ChIPmentation-H3K27ac_A00266_0265_2_S12_L002_I_peaks.narrowPeak
peaks/h3k27ac_ref_peaks/EU05_Flu_2-571412_ChIPmentation-H3K27ac_A00266_0265_2_S6_L002_I_peaks.narrowPeak
peaks/h3k27ac_ref_peaks/AF22_Flu_2-669473_ChIPmentation-H3K27ac_A00266_0289_1_S9_L001_I_peaks.narrowPeak
peaks/h3k27ac_ref_peaks/EU47_Flu_2-669480_ChIPmentation-H3K27ac_A00266_0289_1_S16_L001_I_peaks.narrowPeak
peaks/h3k27ac_ref_peaks/EU19_Flu_2-571338_ChIPmentation-H3K27ac_A00266_0263_3_S6_L003_I_peaks.narrowPeak
peaks/h3k27ac_ref_peaks/AF30_Flu_2-669476_ChIPmentation-H3K27ac_A00266_0289_1_S12_L001_I_peaks.narrowPeak
peaks/h3k27ac_ref_peaks/EU09_Flu_2-571335_ChIPmentation-H3K27ac_A00266_0263_3_S3_L003_I_peaks.narrowPeak
peaks/h3k27ac_ref_peaks/AF26_Flu_2-571344_ChIPmentation-H3K27ac_A00266_0263_3_S12_L003_I_peaks.narrowPeak
peaks/h3k27ac_ref_peaks/EU33_Flu_2-669466_ChIPmentation-H3K27ac_A00266_0289_1_S2_L001_I_peaks.narrowPeak
peaks/h3k27ac_ref_peaks/EU25_Flu_2-571343_ChIPmentation-H3K27ac_A00266_0263_3_S11_L003_I_peaks.narrowPeak
peaks/h3k27ac_ref_peaks/AF38_Flu_2-669483_ChIPmentation-H3K27ac_A00266_0289_1_S19_L001_I_peaks.narrowPeak
peaks/h3k27ac_ref_peaks/EU21_Flu_2-571406_ChIPmentation-H3K27ac_A00266_0263_3_S24_L003_I_peaks.narrowPeak
peaks/h3k27ac_ref_peaks/AF06_Flu_2-642071_ChIPmentation-H3K27ac_A00266_0265_2_S15_L002_I_peaks.narrowPeak
peaks/h3k27ac_ref_peaks/EU15_Flu_2-571342_ChIPmentation-H3K27ac_A00266_0263_3_S10_L003_I_peaks.narrowPeak
peaks/h3k27ac_ref_peaks/AF16_Flu_2-571407_ChIPmentation-H3K27ac_A00266_0265_2_S1_L002_I_peaks.narrowPeak
peaks/h3k27ac_ref_peaks/AF08_Flu_2-571405_ChIPmentation-H3K27ac_A00266_0263_3_S23_L003_I_peaks.narrowPeak
peaks/h3k27ac_ref_peaks/EU13_Flu_2-571348_ChIPmentation-H3K27ac_A00266_0263_3_S16_L003_I_peaks.narrowPeak
peaks/h3k27ac_ref_peaks/AF36_Flu_2-571346_ChIPmentation-H3K27ac_A00266_0263_3_S14_L003_I_peaks.narrowPeak
peaks/h3k27ac_ref_peaks/AF34_Flu_2-571349_ChIPmentation-H3K27ac_A00266_0263_3_S17_L003_I_peaks.narrowPeak
peaks/h3k27ac_ref_peaks/AF12_Flu_2-571340_ChIPmentation-H3K27ac_A00266_0263_3_S8_L003_I_peaks.narrowPeak
peaks/h3k27ac_ref_peaks/EU03_Flu_2-571411_ChIPmentation-H3K27ac_A00266_0265_2_S5_L002_I_peaks.narrowPeak
peaks/h3k27ac_ref_peaks/EU41_Flu_2-669478_ChIPmentation-H3K27ac_A00266_0289_1_S14_L001_I_peaks.narrowPeak
peaks/h3k27ac_ref_peaks/AF20_Flu_2-571333_ChIPmentation-H3K27ac_A00266_0263_3_S1_L003_I_peaks.narrowPeak
peaks/h3k27ac_ref_peaks/EU29_Flu_2-669467_ChIPmentation-H3K27ac_A00266_0289_1_S3_L001_I_peaks.narrowPeak
peaks/h3k27ac_ref_peaks/AF04_Flu_2-642070_ChIPmentation-H3K27ac_A00266_0265_2_S14_L002_I_peaks.narrowPeak
peaks/h3k27ac_ref_peaks/EU39_Flu_2-571401_ChIPmentation-H3K27ac_A00266_0263_3_S19_L003_I_peaks.narrowPeak
peaks/h3k27ac_ref_peaks/AF28_Flu_2-571345_ChIPmentation-H3K27ac_A00266_0263_3_S13_L003_I_peaks.narrowPeak
peaks/h3k27ac_ref_peaks/EU07_Flu_2-642067_ChIPmentation-H3K27ac_A00266_0265_2_S11_L002_I_peaks.narrowPeak
peaks/h3k27ac_ref_peaks/EU27_Flu_2-642077_ChIPmentation-H3K27ac_A00266_0265_2_S21_L002_I_peaks.narrowPeak
peaks/atac_ref_peaks/EU29_Flu_ATACSeq_1_2-268563_S10_L001_I_peaks.narrowPeak
peaks/atac_ref_peaks/EU09_Flu_ATACSeq_1_2-259471_S11_L001_I_peaks.narrowPeak
peaks/atac_ref_peaks/EU13_Flu_ATACSeq_1_2-259477_S1_L002_I_peaks.narrowPeak
peaks/atac_ref_peaks/EU05_Flu_ATACSeq_1_2-259464_S4_L001_I_peaks.narrowPeak
peaks/atac_ref_peaks/AF10_Flu_ATACSeq_1_2-259491_S15_L002_I_peaks.narrowPeak
peaks/atac_ref_peaks/EU19_Flu_ATACSeq_1_2-259485_S9_L002_I_peaks.narrowPeak
peaks/atac_ref_peaks/AF22_Flu_ATACSeq_1_2-308160_S3_L001_I_peaks.narrowPeak
peaks/atac_ref_peaks/AF14_Flu_ATACSeq_1_2-268556_S3_L001_I_peaks.narrowPeak
peaks/atac_ref_peaks/EU33_Flu_ATACSeq_1_2-308170_S13_L001_I_peaks.narrowPeak
peaks/atac_ref_peaks/EU37_Flu_ATACSeq_1_2-308172_S15_L001_I_peaks.narrowPeak
peaks/atac_ref_peaks/AF18_Flu_ATACSeq_1_2-268567_S14_L001_I_peaks.narrowPeak
peaks/atac_ref_peaks/EU47_Flu_ATACSeq_1_2-308183_S10_L002_I_peaks.narrowPeak
peaks/atac_ref_peaks/EU27_Flu_ATACSeq_1_2-268561_S8_L001_I_peaks.narrowPeak
peaks/atac_ref_peaks/AF26_Flu_ATACSeq_1_2-308164_S7_L001_I_peaks.narrowPeak
peaks/atac_ref_peaks/EU43_Flu_ATACSeq_1_2-308181_S8_L002_I_peaks.narrowPeak
peaks/atac_ref_peaks/EU21_Flu_ATACSeq_1_2-259488_S12_L002_I_peaks.narrowPeak
peaks/atac_ref_peaks/AF34_Flu_ATACSeq_1_2-308174_S17_L001_I_peaks.narrowPeak
peaks/atac_ref_peaks/AF28_Flu_ATACSeq_1_2-308166_S9_L001_I_peaks.narrowPeak
peaks/atac_ref_peaks/EU07_Flu_ATACSeq_1_2-259468_S8_L001_I_peaks.narrowPeak
peaks/atac_ref_peaks/EU41_Flu_ATACSeq_1_2-308179_S6_L002_I_peaks.narrowPeak
peaks/atac_ref_peaks/EU03_Flu_ATACSeq_1_2-259462_S2_L001_I_peaks.narrowPeak
peaks/atac_ref_peaks/EU17_Flu_ATACSeq_1_2-308175_S2_L002_I_peaks.narrowPeak
peaks/atac_ref_peaks/AF16_Flu_ATACSeq_1_2-268565_S12_L001_I_peaks.narrowPeak
peaks/atac_ref_peaks/AF20_Flu_ATACSeq_1_2-308158_S1_L001_I_peaks.narrowPeak
peaks/atac_ref_peaks/EU25_Flu_ATACSeq_1_2-268559_S6_L001_I_peaks.narrowPeak
peaks/atac_ref_peaks/EU15_Flu_ATACSeq_1_2-259481_S5_L002_I_peaks.narrowPeak
peaks/atac_ref_peaks/EU39_Flu_ATACSeq_1_2-308177_S4_L002_I_peaks.narrowPeak
peaks/atac_ref_peaks/AF04_Flu_ATACSeq_1_2-259466_S6_L001_I_peaks.narrowPeak
peaks/atac_ref_peaks/AF08_Flu_ATACSeq_1_2-259483_S7_L002_I_peaks.narrowPeak
peaks/atac_ref_peaks/AF12_Flu_ATACSeq_1_2-268554_S1_L001_I_peaks.narrowPeak
peaks/atac_ref_peaks/AF06_Flu_ATACSeq_1_2-259479_S3_L002_I_peaks.narrowPeak
peaks/atac_ref_peaks/AF38_Flu_ATACSeq_1_2-308187_S14_L002_I_peaks.narrowPeak
peaks/atac_ref_peaks/AF30_Flu_ATACSeq_1_2-308168_S11_L001_I_peaks.narrowPeak
peaks/atac_ref_peaks/AF24_Flu_ATACSeq_1_2-308162_S5_L001_I_peaks.narrowPeak
peaks/atac_hprc_peaks/EU29_Flu_ATACSeq_1_2-268563_S10_L001_I_peaks.narrowPeak
peaks/atac_hprc_peaks/EU09_Flu_ATACSeq_1_2-259471_S11_L001_I_peaks.narrowPeak
peaks/atac_hprc_peaks/EU13_Flu_ATACSeq_1_2-259477_S1_L002_I_peaks.narrowPeak
peaks/atac_hprc_peaks/EU05_Flu_ATACSeq_1_2-259464_S4_L001_I_peaks.narrowPeak
peaks/atac_hprc_peaks/AF10_Flu_ATACSeq_1_2-259491_S15_L002_I_peaks.narrowPeak
peaks/atac_hprc_peaks/EU19_Flu_ATACSeq_1_2-259485_S9_L002_I_peaks.narrowPeak

peaks/atac_hprc_peaks/AF22_Flu_ATACSeq_1_2-308160_S3_L001_I_peaks.narrowPeak
peaks/atac_hprc_peaks/AF14_Flu_ATACSeq_1_2-268556_S3_L001_I_peaks.narrowPeak
peaks/atac_hprc_peaks/EU33_Flu_ATACSeq_1_2-308170_S13_L001_I_peaks.narrowPeak
peaks/atac_hprc_peaks/EU37_Flu_ATACSeq_1_2-308172_S15_L001_I_peaks.narrowPeak
peaks/atac_hprc_peaks/AF18_Flu_ATACSeq_1_2-268567_S14_L001_I_peaks.narrowPeak
peaks/atac_hprc_peaks/EU47_Flu_ATACSeq_1_2-308183_S10_L002_I_peaks.narrowPeak
peaks/atac_hprc_peaks/EU27_Flu_ATACSeq_1_2-268561_S8_L001_I_peaks.narrowPeak
peaks/atac_hprc_peaks/AF26_Flu_ATACSeq_1_2-308164_S7_L001_I_peaks.narrowPeak
peaks/atac_hprc_peaks/EU43_Flu_ATACSeq_1_2-308181_S8_L002_I_peaks.narrowPeak
peaks/atac_hprc_peaks/EU21_Flu_ATACSeq_1_2-259488_S12_L002_I_peaks.narrowPeak
peaks/atac_hprc_peaks/AF34_Flu_ATACSeq_1_2-308174_S17_L001_I_peaks.narrowPeak
peaks/atac_hprc_peaks/AF28_Flu_ATACSeq_1_2-308166_S9_L001_I_peaks.narrowPeak
peaks/atac_hprc_peaks/EU07_Flu_ATACSeq_1_2-259468_S8_L001_I_peaks.narrowPeak
peaks/atac_hprc_peaks/EU41_Flu_ATACSeq_1_2-308179_S6_L002_I_peaks.narrowPeak
peaks/atac_hprc_peaks/EU03_Flu_ATACSeq_1_2-259462_S2_L001_I_peaks.narrowPeak
peaks/atac_hprc_peaks/EU17_Flu_ATACSeq_1_2-308175_S2_L002_I_peaks.narrowPeak
peaks/atac_hprc_peaks/AF16_Flu_ATACSeq_1_2-268565_S12_L001_I_peaks.narrowPeak
peaks/atac_hprc_peaks/AF20_Flu_ATACSeq_1_2-308158_S1_L001_I_peaks.narrowPeak
peaks/atac_hprc_peaks/EU25_Flu_ATACSeq_1_2-268559_S6_L001_I_peaks.narrowPeak
peaks/atac_hprc_peaks/EU15_Flu_ATACSeq_1_2-259481_S5_L002_I_peaks.narrowPeak
peaks/atac_hprc_peaks/EU39_Flu_ATACSeq_1_2-308177_S4_L002_I_peaks.narrowPeak
peaks/atac_hprc_peaks/AF04_Flu_ATACSeq_1_2-259466_S6_L001_I_peaks.narrowPeak
peaks/atac_hprc_peaks/AF36_Flu_ATACSeq_1_2-308185_S12_L002_I_peaks.narrowPeak
peaks/atac_hprc_peaks/AF08_Flu_ATACSeq_1_2-259483_S7_L002_I_peaks.narrowPeak
peaks/atac_hprc_peaks/AF12_Flu_ATACSeq_1_2-268554_S1_L001_I_peaks.narrowPeak
peaks/atac_hprc_peaks/AF06_Flu_ATACSeq_1_2-259479_S3_L002_I_peaks.narrowPeak
peaks/atac_hprc_peaks/AF38_Flu_ATACSeq_1_2-308187_S14_L002_I_peaks.narrowPeak
peaks/atac_hprc_peaks/AF30_Flu_ATACSeq_1_2-308168_S11_L001_I_peaks.narrowPeak
peaks/atac_hprc_peaks/AF24_Flu_ATACSeq_1_2-308162_S5_L001_I_peaks.narrowPeak
peaks/h3k4me1_hprc_peaks/AF28_Flu_2-669592_ChIPmentation-H3K4me1_A00266_0289_2_S9_L002_I_peaks.narrowPeak
peaks/h3k4me1_hprc_peaks/AF30_Flu_2-674830_ChIPmentation-H3K4me1_A00266_0301_2_S7_L002_I_peaks.narrowPeak
peaks/h3k4me1_hprc_peaks/EU27_Flu_2-674815_ChIPmentation-
H3K4me1A00266_0301_1_S16_L001_I_peaks.narrowPeak
peaks/h3k4me1_hprc_peaks/EU05_Flu_2-674801_ChIPmentation-H3K4me1A00266_0301_1_S2_L001_I_peaks.narrowPeak
peaks/h3k4me1_hprc_peaks/AF18_Flu_2-674803_ChIPmentation-H3K4me1_A00266_0301_1_S4_L001_I_peaks.narrowPeak
peaks/h3k4me1_hprc_peaks/EU47_Flu_2-674834_ChIPmentation-
H3K4me1A00266_0301_2_S11_L002_I_peaks.narrowPeak
peaks/h3k4me1_hprc_peaks/AF34_Flu_2-669596_ChIPmentation-
H3K4me1_A00266_0289_2_S13_L002_I_peaks.narrowPeak
peaks/h3k4me1_hprc_peaks/EU09_Flu_2-669582_ChIPmentation-
H3K4me1A00266_0289_1_S23_L001_I_peaks.narrowPeak
peaks/h3k4me1_hprc_peaks/AF20_Flu_2-669580_ChIPmentation-
H3K4me1_A00266_0289_1_S21_L001_I_peaks.narrowPeak
peaks/h3k4me1_hprc_peaks/EU39_Flu_2-669598_ChIPmentation-
H3K4me1A00266_0289_2_S15_L002_I_peaks.narrowPeak
peaks/h3k4me1_hprc_peaks/EU03_Flu_2-674750_ChIPmentation-H3K4me1A00266_0301_1_S1_L001_I_peaks.narrowPeak
peaks/h3k4me1_hprc_peaks/EU15_Flu_2-669589_ChIPmentation-H3K4me1A00266_0289_2_S6_L002_I_peaks.narrowPeak
peaks/h3k4me1_hprc_peaks/AF12_Flu_2-669587_ChIPmentation-H3K4me1_A00266_0289_2_S4_L002_I_peaks.narrowPeak
peaks/h3k4me1_hprc_peaks/EU25_Flu_2-669590_ChIPmentation-H3K4me1A00266_0289_2_S7_L002_I_peaks.narrowPeak
peaks/h3k4me1_hprc_peaks/AF24_Flu_2-674804_ChIPmentation-H3K4me1_A00266_0301_1_S5_L001_I_peaks.narrowPeak
peaks/h3k4me1_hprc_peaks/AF36_Flu_2-669593_ChIPmentation-
H3K4me1_A00266_0289_2_S10_L002_I_peaks.narrowPeak
peaks/h3k4me1_hprc_peaks/AF04_Flu_2-674805_ChIPmentation-H3K4me1_A00266_0301_1_S6_L001_I_peaks.narrowPeak
peaks/h3k4me1_hprc_peaks/EU13_Flu_2-669595_ChIPmentation-
H3K4me1A00266_0289_2_S12_L002_I_peaks.narrowPeak
peaks/h3k4me1_hprc_peaks/EU07_Flu_2-674802_ChIPmentation-H3K4me1A00266_0301_1_S3_L001_I_peaks.narrowPeak
peaks/h3k4me1_hprc_peaks/AF38_Flu_2-674837_ChIPmentation-
H3K4me1_A00266_0301_2_S14_L002_I_peaks.narrowPeak
peaks/h3k4me1_hprc_peaks/AF06_Flu_2-674806_ChIPmentation-H3K4me1_A00266_0301_1_S7_L001_I_peaks.narrowPeak
peaks/h3k4me1_hprc_peaks/AF22_Flu_2-674827_ChIPmentation-H3K4me1_A00266_0301_2_S4_L002_I_peaks.narrowPeak
peaks/h3k4me1_hprc_peaks/EU41_Flu_2-674832_ChIPmentation-H3K4me1A00266_0301_2_S9_L002_I_peaks.narrowPeak
peaks/h3k4me1_hprc_peaks/EU29_Flu_2-674821_ChIPmentation-
H3K4me1A00266_0301_1_S22_L001_I_peaks.narrowPeak
peaks/h3k4me1_hprc_peaks/AF26_Flu_2-669591_ChIPmentation-H3K4me1_A00266_0289_2_S8_L002_I_peaks.narrowPeak
peaks/h3k4me1_hprc_peaks/EU19_Flu_2-669585_ChIPmentation-H3K4me1A00266_0289_2_S2_L002_I_peaks.narrowPeak
peaks/h3k4me1_hprc_peaks/AF16_Flu_2-669654_ChIPmentation-
H3K4me1_A00266_0289_2_S21_L002_I_peaks.narrowPeak
peaks/h3k4me1_hprc_peaks/AF08_Flu_2-669652_ChIPmentation-
H3K4me1_A00266_0289_2_S19_L002_I_peaks.narrowPeak
peaks/h3k4me1_hprc_peaks/EU33_Flu_2-674820_ChIPmentation-
H3K4me1A00266_0301_1_S21_L001_I_peaks.narrowPeak
peaks/h3k4me1_hprc_peaks/EU21_Flu_2-669653_ChIPmentation-
H3K4me1A00266_0289_2_S20_L002_I_peaks.narrowPeak
peaks/atac_overlaps/AF10_Flu_ATACSeq_1_2-259491_S15_L002_I_peaks.narrowPeak_intersect.bed
peaks/atac_overlaps/EU17_Flu_ATACSeq_1_2-308175_S2_L002_I_peaks.narrowPeak_ref-only.bed
peaks/atac_overlaps/EU07_Flu_ATACSeq_1_2-259468_S8_L001_I_peaks.narrowPeak_ref-only.bed

```
peaks/atac_overlaps/EU47_Flu_ATACSeq_1_2-308183_S10_L002_I_peaks.narrowPeak_pers-only.bed
peaks/atac_overlaps/EU37_Flu_ATACSeq_1_2-308172_S15_L001_I_peaks.narrowPeak_ref-only.bed
peaks/atac_overlaps/EU41_Flu_ATACSeq_1_2-308179_S6_L002_I_peaks.narrowPeak_ref-only.bed
peaks/atac_overlaps/EU47_Flu_ATACSeq_1_2-308183_S10_L002_I_peaks.narrowPeak_ref-only.bed
peaks/atac_overlaps/AF20_Flu_ATACSeq_1_2-308158_S1_L001_I_peaks.narrowPeak_pers-only.bed
peaks/atac_overlaps/AF14_Flu_ATACSeq_1_2-268556_S3_L001_I_peaks.narrowPeak_intersect.bed
peaks/atac_overlaps/EU19_Flu_ATACSeq_1_2-259485_S9_L002_I_peaks.narrowPeak_pers-only.bed
peaks/atac_overlaps/EU15_Flu_ATACSeq_1_2-259481_S5_L002_I_peaks.narrowPeak_ref-only.bed
peaks/atac_overlaps/EU25_Flu_ATACSeq_1_2-268559_S6_L001_I_peaks.narrowPeak_pers-only.bed
peaks/atac_overlaps/EU33_Flu_ATACSeq_1_2-308170_S13_L001_I_peaks.narrowPeak_pers-only.bed
peaks/atac_overlaps/AF04_Flu_ATACSeq_1_2-259466_S6_L001_I_peaks.narrowPeak_intersect.bed
peaks/atac_overlaps/AF22_Flu_ATACSeq_1_2-308160_S3_L001_I_peaks.narrowPeak_pers-only.bed
peaks/atac_overlaps/EU13_Flu_ATACSeq_1_2-259477_S1_L002_I_peaks.narrowPeak_ref-only.bed
peaks/atac_overlaps/AF34_Flu_ATACSeq_1_2-308174_S17_L001_I_peaks.narrowPeak_intersect.bed
peaks/atac_overlaps/EU17_Flu_ATACSeq_1_2-308175_S2_L002_I_peaks.narrowPeak_intersect.bed
peaks/atac_overlaps/EU13_Flu_ATACSeq_1_2-259477_S1_L002_I_peaks.narrowPeak_pers-only.bed
peaks/atac_overlaps/AF38_Flu_ATACSeq_1_2-308187_S14_L002_I_peaks.narrowPeak_intersect.bed
peaks/atac_overlaps/AF22_Flu_ATACSeq_1_2-308160_S3_L001_I_peaks.narrowPeak_ref-only.bed
peaks/atac_overlaps/AF28_Flu_ATACSeq_1_2-308166_S9_L001_I_peaks.narrowPeak_intersect.bed
peaks/atac_overlaps/EU03_Flu_ATACSeq_1_2-259462_S2_L001_I_peaks.narrowPeak_pers-only.bed
peaks/atac_overlaps/EU21_Flu_ATACSeq_1_2-259488_S12_L002_I_peaks.narrowPeak_ref-only.bed
peaks/atac_overlaps/EU03_Flu_ATACSeq_1_2-259462_S2_L001_I_peaks.narrowPeak_intersect.bed
peaks/atac_overlaps/AF28_Flu_ATACSeq_1_2-308166_S9_L001_I_peaks.narrowPeak_pers-only.bed
peaks/atac_overlaps/AF38_Flu_ATACSeq_1_2-308187_S14_L002_I_peaks.narrowPeak_pers-only.bed
peaks/atac_overlaps/EU25_Flu_ATACSeq_1_2-268559_S6_L001_I_peaks.narrowPeak_ref-only.bed
peaks/atac_overlaps/EU05_Flu_ATACSeq_1_2-259464_S4_L001_I_peaks.narrowPeak_ref-only.bed
peaks/atac_overlaps/EU13_Flu_ATACSeq_1_2-259477_S1_L002_I_peaks.narrowPeak_intersect.bed
peaks/atac_overlaps/EU17_Flu_ATACSeq_1_2-308175_S2_L002_I_peaks.narrowPeak_pers-only.bed
peaks/atac_overlaps/AF34_Flu_ATACSeq_1_2-308174_S17_L001_I_peaks.narrowPeak_pers-only.bed
peaks/atac_overlaps/AF26_Flu_ATACSeq_1_2-308164_S7_L001_I_peaks.narrowPeak_ref-only.bed
peaks/atac_overlaps/AF04_Flu_ATACSeq_1_2-259466_S6_L001_I_peaks.narrowPeak_pers-only.bed
peaks/atac_overlaps/AF22_Flu_ATACSeq_1_2-308160_S3_L001_I_peaks.narrowPeak_intersect.bed
peaks/atac_overlaps/AF30_Flu_ATACSeq_1_2-308168_S11_L001_I_peaks.narrowPeak_ref-only.bed
peaks/atac_overlaps/EU33_Flu_ATACSeq_1_2-308170_S13_L001_I_peaks.narrowPeak_intersect.bed
peaks/atac_overlaps/EU09_Flu_ATACSeq_1_2-259471_S11_L001_I_peaks.narrowPeak_ref-only.bed
peaks/atac_overlaps/EU29_Flu_ATACSeq_1_2-268563_S10_L001_I_peaks.narrowPeak_ref-only.bed
peaks/atac_overlaps/AF08_Flu_ATACSeq_1_2-259483_S7_L002_I_peaks.narrowPeak_ref-only.bed
peaks/atac_overlaps/EU25_Flu_ATACSeq_1_2-268559_S6_L001_I_peaks.narrowPeak_intersect.bed
peaks/atac_overlaps/EU19_Flu_ATACSeq_1_2-259485_S9_L002_I_peaks.narrowPeak_intersect.bed
peaks/atac_overlaps/AF14_Flu_ATACSeq_1_2-268556_S3_L001_I_peaks.narrowPeak_pers-only.bed
peaks/atac_overlaps/AF38_Flu_ATACSeq_1_2-308187_S14_L002_I_peaks.narrowPeak_ref-only.bed
peaks/atac_overlaps/AF20_Flu_ATACSeq_1_2-308158_S1_L001_I_peaks.narrowPeak_intersect.bed
peaks/atac_overlaps/EU19_Flu_ATACSeq_1_2-259485_S9_L002_I_peaks.narrowPeak_ref-only.bed
peaks/atac_overlaps/EU47_Flu_ATACSeq_1_2-308183_S10_L002_I_peaks.narrowPeak_intersect.bed
peaks/atac_overlaps/EU33_Flu_ATACSeq_1_2-308170_S13_L001_I_peaks.narrowPeak_ref-only.bed
peaks/atac_overlaps/AF10_Flu_ATACSeq_1_2-259491_S15_L002_I_peaks.narrowPeak_pers-only.bed
peaks/atac_overlaps/EU05_Flu_ATACSeq_1_2-259464_S4_L001_I_peaks.narrowPeak_intersect.bed
peaks/atac_overlaps/EU21_Flu_ATACSeq_1_2-259488_S12_L002_I_peaks.narrowPeak_intersect.bed
peaks/atac_overlaps/AF24_Flu_ATACSeq_1_2-308162_S5_L001_I_peaks.narrowPeak_intersect.bed
peaks/atac_overlaps/AF10_Flu_ATACSeq_1_2-259491_S15_L002_I_peaks.narrowPeak_ref-only.bed
peaks/atac_overlaps/EU41_Flu_ATACSeq_1_2-308179_S6_L002_I_peaks.narrowPeak_pers-only.bed
peaks/atac_overlaps/AF16_Flu_ATACSeq_1_2-268565_S12_L001_I_peaks.narrowPeak_pers-only.bed
peaks/atac_overlaps/EU37_Flu_ATACSeq_1_2-308172_S15_L001_I_peaks.narrowPeak_pers-only.bed
peaks/atac_overlaps/AF30_Flu_ATACSeq_1_2-308168_S11_L001_I_peaks.narrowPeak_intersect.bed
peaks/atac_overlaps/AF06_Flu_ATACSeq_1_2-259479_S3_L002_I_peaks.narrowPeak_intersect.bed
peaks/atac_overlaps/EU43_Flu_ATACSeq_1_2-308181_S8_L002_I_peaks.narrowPeak_ref-only.bed
peaks/atac_overlaps/AF18_Flu_ATACSeq_1_2-268567_S14_L001_I_peaks.narrowPeak_ref-only.bed
peaks/atac_overlaps/AF08_Flu_ATACSeq_1_2-259483_S7_L002_I_peaks.narrowPeak_intersect.bed
peaks/atac_overlaps/EU43_Flu_ATACSeq_1_2-308181_S8_L002_I_peaks.narrowPeak_intersect.bed
peaks/atac_overlaps/AF18_Flu_ATACSeq_1_2-268567_S14_L001_I_peaks.narrowPeak_intersect.bed
peaks/atac_overlaps/EU29_Flu_ATACSeq_1_2-268563_S10_L001_I_peaks.narrowPeak_intersect.bed
peaks/atac_overlaps/EU27_Flu_ATACSeq_1_2-268561_S8_L001_I_peaks.narrowPeak_pers-only.bed
peaks/atac_overlaps/AF26_Flu_ATACSeq_1_2-308164_S7_L001_I_peaks.narrowPeak_pers-only.bed
peaks/atac_overlaps/AF28_Flu_ATACSeq_1_2-308166_S9_L001_I_peaks.narrowPeak_ref-only.bed
peaks/atac_overlaps/AF12_Flu_ATACSeq_1_2-268554_S1_L001_I_peaks.narrowPeak_pers-only.bed
peaks/atac_overlaps/EU15_Flu_ATACSeq_1_2-259481_S5_L002_I_peaks.narrowPeak_pers-only.bed
peaks/atac_overlaps/EU07_Flu_ATACSeq_1_2-259468_S8_L001_I_peaks.narrowPeak_intersect.bed
peaks/atac_overlaps/EU39_Flu_ATACSeq_1_2-308177_S4_L002_I_peaks.narrowPeak_pers-only.bed
peaks/atac_overlaps/EU09_Flu_ATACSeq_1_2-259471_S11_L001_I_peaks.narrowPeak_pers-only.bed
peaks/atac_overlaps/AF34_Flu_ATACSeq_1_2-308174_S17_L001_I_peaks.narrowPeak_ref-only.bed
peaks/atac_overlaps/AF24_Flu_ATACSeq_1_2-308162_S5_L001_I_peaks.narrowPeak_ref-only.bed
peaks/atac_overlaps/EU09_Flu_ATACSeq_1_2-259471_S11_L001_I_peaks.narrowPeak_intersect.bed
peaks/atac_overlaps/EU03_Flu_ATACSeq_1_2-259462_S2_L001_I_peaks.narrowPeak_ref-only.bed
peaks/atac_overlaps/EU39_Flu_ATACSeq_1_2-308177_S4_L002_I_peaks.narrowPeak_intersect.bed
peaks/atac_overlaps/EU07_Flu_ATACSeq_1_2-259468_S8_L001_I_peaks.narrowPeak_pers-only.bed
peaks/atac_overlaps/EU15_Flu_ATACSeq_1_2-259481_S5_L002_I_peaks.narrowPeak_intersect.bed
```

```
peaks/atac_overlaps/AF20_Flu_ATACSeq_1_2-308158_S1_L001_I_peaks.narrowPeak_ref-only.bed
peaks/atac_overlaps/AF12_Flu_ATACSeq_1_2-268554_S1_L001_I_peaks.narrowPeak_intersect.bed
peaks/atac_overlaps/AF14_Flu_ATACSeq_1_2-268556_S3_L001_I_peaks.narrowPeak_ref-only.bed
peaks/atac_overlaps/AF26_Flu_ATACSeq_1_2-308164_S7_L001_I_peaks.narrowPeak_intersect.bed
peaks/atac_overlaps/EU27_Flu_ATACSeq_1_2-268561_S8_L001_I_peaks.narrowPeak_intersect.bed
peaks/atac_overlaps/EU29_Flu_ATACSeq_1_2-268563_S10_L001_I_peaks.narrowPeak_pers-only.bed
peaks/atac_overlaps/AF18_Flu_ATACSeq_1_2-268567_S14_L001_I_peaks.narrowPeak_pers-only.bed
peaks/atac_overlaps/AF16_Flu_ATACSeq_1_2-268565_S12_L001_I_peaks.narrowPeak_ref-only.bed
peaks/atac_overlaps/EU43_Flu_ATACSeq_1_2-308181_S8_L002_I_peaks.narrowPeak_pers-only.bed
peaks/atac_overlaps/AF06_Flu_ATACSeq_1_2-259479_S3_L002_I_peaks.narrowPeak_ref-only.bed
peaks/atac_overlaps/AF08_Flu_ATACSeq_1_2-259483_S7_L002_I_peaks.narrowPeak_pers-only.bed
peaks/atac_overlaps/AF06_Flu_ATACSeq_1_2-259479_S3_L002_I_peaks.narrowPeak_pers-only.bed
peaks/atac_overlaps/AF30_Flu_ATACSeq_1_2-308168_S11_L001_I_peaks.narrowPeak_pers-only.bed
peaks/atac_overlaps/EU37_Flu_ATACSeq_1_2-308172_S15_L001_I_peaks.narrowPeak_intersect.bed
peaks/atac_overlaps/EU27_Flu_ATACSeq_1_2-268561_S8_L001_I_peaks.narrowPeak_ref-only.bed
peaks/atac_overlaps/AF16_Flu_ATACSeq_1_2-268565_S12_L001_I_peaks.narrowPeak_intersect.bed
peaks/atac_overlaps/AF04_Flu_ATACSeq_1_2-259466_S6_L001_I_peaks.narrowPeak_ref-only.bed
peaks/atac_overlaps/EU41_Flu_ATACSeq_1_2-308179_S6_L002_I_peaks.narrowPeak_intersect.bed
peaks/atac_overlaps/AF24_Flu_ATACSeq_1_2-308162_S5_L001_I_peaks.narrowPeak_pers-only.bed
peaks/atac_overlaps/EU21_Flu_ATACSeq_1_2-259488_S12_L002_I_peaks.narrowPeak_pers-only.bed
peaks/atac_overlaps/EU05_Flu_ATACSeq_1_2-259464_S4_L001_I_peaks.narrowPeak_pers-only.bed
peaks/atac_overlaps/EU39_Flu_ATACSeq_1_2-308177_S4_L002_I_peaks.narrowPeak_ref-only.bed
peaks/atac_overlaps/AF12_Flu_ATACSeq_1_2-268554_S1_L001_I_peaks.narrowPeak_ref-only.bed
peaks/h3k4me1_ref_peaks/AF28_Flu_2-669592_ChIPmentation-H3K4me1_A00266_0289_2_S9_L002_I_peaks.narrowPeak
peaks/h3k4me1_ref_peaks/AF30_Flu_2-674830_ChIPmentation-H3K4me1_A00266_0301_2_S7_L002_I_peaks.narrowPeak
peaks/h3k4me1_ref_peaks/EU27_Flu_2-674815_ChIPmentation-H3K4me1A00266_0301_1_S16_L001_I_peaks.narrowPeak
peaks/h3k4me1_ref_peaks/EU05_Flu_2-674801_ChIPmentation-H3K4me1A00266_0301_1_S2_L001_I_peaks.narrowPeak
peaks/h3k4me1_ref_peaks/AF18_Flu_2-674803_ChIPmentation-H3K4me1_A00266_0301_1_S4_L001_I_peaks.narrowPeak
peaks/h3k4me1_ref_peaks/EU47_Flu_2-674834_ChIPmentation-H3K4me1A00266_0301_2_S11_L002_I_peaks.narrowPeak
peaks/h3k4me1_ref_peaks/AF34_Flu_2-669596_ChIPmentation-H3K4me1_A00266_0289_2_S13_L002_I_peaks.narrowPeak
peaks/h3k4me1_ref_peaks/EU09_Flu_2-669582_ChIPmentation-H3K4me1A00266_0289_1_S23_L001_I_peaks.narrowPeak
peaks/h3k4me1_ref_peaks/AF20_Flu_2-669580_ChIPmentation-H3K4me1_A00266_0289_1_S21_L001_I_peaks.narrowPeak
peaks/h3k4me1_ref_peaks/EU39_Flu_2-669598_ChIPmentation-H3K4me1A00266_0289_2_S15_L002_I_peaks.narrowPeak
peaks/h3k4me1_ref_peaks/EU03_Flu_2-674750_ChIPmentation-H3K4me1A00266_0301_1_S1_L001_I_peaks.narrowPeak
peaks/h3k4me1_ref_peaks/EU15_Flu_2-669589_ChIPmentation-H3K4me1A00266_0289_2_S6_L002_I_peaks.narrowPeak
peaks/h3k4me1_ref_peaks/AF12_Flu_2-669587_ChIPmentation-H3K4me1_A00266_0289_2_S4_L002_I_peaks.narrowPeak
peaks/h3k4me1_ref_peaks/EU25_Flu_2-669590_ChIPmentation-H3K4me1A00266_0289_2_S7_L002_I_peaks.narrowPeak
peaks/h3k4me1_ref_peaks/AF24_Flu_2-674804_ChIPmentation-H3K4me1_A00266_0301_1_S5_L001_I_peaks.narrowPeak
peaks/h3k4me1_ref_peaks/AF36_Flu_2-669593_ChIPmentation-H3K4me1_A00266_0289_2_S10_L002_I_peaks.narrowPeak
peaks/h3k4me1_ref_peaks/AF04_Flu_2-674805_ChIPmentation-H3K4me1_A00266_0301_1_S6_L001_I_peaks.narrowPeak
peaks/h3k4me1_ref_peaks/EU13_Flu_2-669595_ChIPmentation-H3K4me1A00266_0289_2_S12_L002_I_peaks.narrowPeak
peaks/h3k4me1_ref_peaks/EU07_Flu_2-674802_ChIPmentation-H3K4me1A00266_0301_1_S3_L001_I_peaks.narrowPeak
peaks/h3k4me1_ref_peaks/AF38_Flu_2-674837_ChIPmentation-H3K4me1_A00266_0301_2_S14_L002_I_peaks.narrowPeak
peaks/h3k4me1_ref_peaks/AF06_Flu_2-674806_ChIPmentation-H3K4me1_A00266_0301_1_S7_L001_I_peaks.narrowPeak
peaks/h3k4me1_ref_peaks/AF22_Flu_2-674827_ChIPmentation-H3K4me1_A00266_0301_2_S4_L002_I_peaks.narrowPeak
peaks/h3k4me1_ref_peaks/EU41_Flu_2-674832_ChIPmentation-H3K4me1A00266_0301_2_S9_L002_I_peaks.narrowPeak
peaks/h3k4me1_ref_peaks/EU29_Flu_2-674821_ChIPmentation-H3K4me1A00266_0301_1_S22_L001_I_peaks.narrowPeak
peaks/h3k4me1_ref_peaks/AF26_Flu_2-669591_ChIPmentation-H3K4me1_A00266_0289_2_S8_L002_I_peaks.narrowPeak
peaks/h3k4me1_ref_peaks/EU19_Flu_2-669585_ChIPmentation-H3K4me1A00266_0289_2_S2_L002_I_peaks.narrowPeak
peaks/h3k4me1_ref_peaks/AF16_Flu_2-669654_ChIPmentation-H3K4me1_A00266_0289_2_S21_L002_I_peaks.narrowPeak
peaks/h3k4me1_ref_peaks/AF08_Flu_2-669652_ChIPmentation-H3K4me1_A00266_0289_2_S19_L002_I_peaks.narrowPeak
peaks/h3k4me1_ref_peaks/EU33_Flu_2-674820_ChIPmentation-H3K4me1A00266_0301_1_S21_L001_I_peaks.narrowPeak
peaks/h3k4me1_ref_peaks/EU21_Flu_2-669653_ChIPmentation-H3K4me1A00266_0289_2_S20_L002_I_peaks.narrowPeak
indel_snarls/
indel_snarls/H3K4me1_indel_matched/
indel_snarls/H3K27ac_indel_matched/
indel_snarls/ATACseq_indel_matched/
indel_snarls/ATACseq_indel_matched/0000.vcf.gz
indel_snarls/ATACseq_indel_matched/0001.vcf.gz.tbi
indel_snarls/ATACseq_indel_matched/sites.txt
indel_snarls/ATACseq_indel_matched/0000.vcf.gz.tbi
indel_snarls/ATACseq_indel_matched/0001.vcf.gz
indel_snarls/ATACseq_indel_matched/README.txt
indel_snarls/H3K27ac_indel_matched/0000.vcf.gz
indel_snarls/H3K27ac_indel_matched/0001.vcf.gz.tbi
indel_snarls/H3K27ac_indel_matched/sites.txt
indel_snarls/H3K27ac_indel_matched/0000.vcf.gz.tbi
indel_snarls/H3K27ac_indel_matched/0001.vcf.gz
indel_snarls/H3K27ac_indel_matched/README.txt
indel_snarls/H3K4me1_indel_matched/0000.vcf.gz
indel_snarls/H3K4me1_indel_matched/0001.vcf.gz.tbi
indel_snarls/H3K4me1_indel_matched/sites.txt
indel_snarls/H3K4me1_indel_matched/0000.vcf.gz.tbi
indel_snarls/H3K4me1_indel_matched/0001.vcf.gz
indel_snarls/H3K4me1_indel_matched/README.txt
align.nf
```

call_peaks.nf
figures.R

Genome browser session (e.g. UCSC): No longer applicable

Methodology

Replicates	1 per sample
Sequencing depth	Sequencing depth H3K27ac: Average 257 million reads per sample<br>H3Kme1: Average 288 million reads per sample<br>ATAC-seq; Average of 279 million reads per sample
Antibodies	H3K27ac (Diagenode antibody, cat # C15410196) and H3K4me1 (Cell Signaling antibody, cat # CST5326). Sequencing data is produced by another study (https://www.biorxiv.org/content/10.1101/2021.09.29.462206v2)
Peak calling parameters	We aligned H3K4me1 and H3K27ac binding sequences obtained from monocyte-derived macrophages from 30 individuals (Groza et al., 2022) using vg map (Garrison et al., 2018) to the hg38 reference genome graph and to the HPRC genome graph. Then, we called peaks using Graph Peak Caller v1.2.3 (Grytten et al., 2019) on both sets of alignments for each of the 30 H3K4me1 and H3K27ac samples.
Data quality	5 million cells per sample, 100 bp paired-end, 150-500bp fragments
Software	vg map and Graph Peak Caller v1.2.3

Flow Cytometry

Plots

Confirm that:

☐ The axis labels state the marker and fluorochrome used (e.g. CD4-FITC).

☐ The axis scales are clearly visible. Include numbers along axes only for bottom left plot of group (a 'group' is an analysis of identical markers).

☐ All plots are contour plots with outliers or pseudocolor plots.

☐ A numerical value for number of cells or percentage (with statistics) is provided.

Methodology

Sample preparation	*Describe the sample preparation, detailing the biological source of the cells and any tissue processing steps used.*
Instrument	*Identify the instrument used for data collection, specifying make and model number.*
Software	*Describe the software used to collect and analyze the flow cytometry data. For custom code that has been deposited into a community repository, provide accession details.*
Cell population abundance	*Describe the abundance of the relevant cell populations within post-sort fractions, providing details on the purity of the samples and how it was determined.*
Gating strategy	*Describe the gating strategy used for all relevant experiments, specifying the preliminary FSC/SSC gates of the starting cell population, indicating where boundaries between "positive" and "negative" staining cell populations are defined.*

☐ Tick this box to confirm that a figure exemplifying the gating strategy is provided in the Supplementary Information.

Magnetic resonance imaging

Experimental design

Design type	*Indicate task or resting state; event-related or block design.*
Design specifications	*Specify the number of blocks, trials or experimental units per session and/or subject, and specify the length of each trial or block (if trials are blocked) and interval between trials.*
Behavioral performance measures	*State number and/or type of variables recorded (e.g. correct button press, response time) and what statistics were used to establish that the subjects were performing the task as expected (e.g. mean, range, and/or standard deviation across subjects).*

Acquisition

Imaging type(s) | *Specify: functional, structural, diffusion, perfusion.*

Field strength | *Specify in Tesla*

Sequence & imaging parameters | *Specify the pulse sequence type (gradient echo, spin echo, etc.), imaging type (EPI, spiral, etc.), field of view, matrix size, slice thickness, orientation and TE/TR/flip angle.*

Area of acquisition | *State whether a whole brain scan was used OR define the area of acquisition, describing how the region was determined.*

Diffusion MRI ☐ Used ☐ Not used

Preprocessing

Preprocessing software | *Provide detail on software version and revision number and on specific parameters (model/functions, brain extraction, segmentation, smoothing kernel size, etc.).*

Normalization | *If data were normalized/standardized, describe the approach(es): specify linear or non-linear and define image types used for transformation OR indicate that data were not normalized and explain rationale for lack of normalization.*

Normalization template | *Describe the template used for normalization/transformation, specifying subject space or group standardized space (e.g. original Talairach, MNI305, ICBM152) OR indicate that the data were not normalized.*

Noise and artifact removal | *Describe your procedure(s) for artifact and structured noise removal, specifying motion parameters, tissue signals and physiological signals (heart rate, respiration).*

Volume censoring | *Define your software and/or method and criteria for volume censoring, and state the extent of such censoring.*

Statistical modeling & inference

Model type and settings | *Specify type (mass univariate, multivariate, RSA, predictive, etc.) and describe essential details of the model at the first and second levels (e.g. fixed, random or mixed effects; drift or auto-correlation).*

Effect(s) tested | *Define precise effect in terms of the task or stimulus conditions instead of psychological concepts and indicate whether ANOVA or factorial designs were used.*

Specify type of analysis: ☐ Whole brain ☐ ROI-based ☐ Both

Statistic type for inference
(See Eklund et al. 2016) | *Specify voxel-wise or cluster-wise and report all relevant parameters for cluster-wise methods.*

Correction | *Describe the type of correction and how it is obtained for multiple comparisons (e.g. FWE, FDR, permutation or Monte Carlo).*

Models & analysis

n/a	Involved in the study
☐	☐ Functional and/or effective connectivity
☐	☐ Graph analysis
☐	☐ Multivariate modeling or predictive analysis

Functional and/or effective connectivity | *Report the measures of dependence used and the model details (e.g. Pearson correlation, partial correlation, mutual information).*

Graph analysis | *Report the dependent variable and connectivity measure, specifying weighted graph or binarized graph, subject- or group-level, and the global and/or node summaries used (e.g. clustering coefficient, efficiency, etc.).*

Multivariate modeling and predictive analysis | *Specify independent variables, features extraction and dimension reduction, model, training and evaluation metrics.*
